# Low Efficacy of Pegylated Interferon plus Ribavirin plus Nitazoxanide for HCV Genotype 4 and HIV Coinfection

**DOI:** 10.1371/journal.pone.0143492

**Published:** 2015-12-07

**Authors:** Juan Macías, Luis F. López-Cortés, Francisco Téllez, Eva Recio, Guillermo Ojeda-Burgos, MªJosé Ríos, Antonio Rivero-Juárez, Marcial Delgado, Rivas- Jeremías, Juan A. Pineda

**Affiliations:** 1 Unidad Clínica de Enfermedades Infecciosas y Microbiología, Hospital Universitario de Valme, Sevilla, Spain; 2 Unidad Clínica de Enfermedades Infecciosas, Microbiología y Medicina Preventiva, Hospital Universitario Virgen del Rocío, Instituto de Biomedicina de Sevilla (IBiS), Sevilla, Spain; 3 Unidad de Gestión Clínica de Enfermedades Infecciosas y Microbiología, Hospital de La Línea de la Concepción, AGS Campo de Gibraltar, Cádiz, Spain; 4 Unidad de Gestión Clínica de Enfermedades Infecciosas, Hospital Virgen de la Victoria, Complejo Hospitalario de Málaga, Málaga, Spain; 5 Unidad de Enfermedades Infecciosas, Hospital Universitario Virgen Macarena, Sevilla, Spain; 6 Unidad de Enfermedades Infecciosas, Hospital Universitario Reina Sofía, Instituto Maimónides de Investigación Biomédica de Córdoba (IMIBIC), Córdoba, Spain; 7 Servicio de Enfermedades Infecciosas, Hospital Regional de Málaga, Malaga, Spain; Taipei Veterans General Hosptial, TAIWAN

## Abstract

**Background:**

Nitazoxanide (NTZ) plus pegylated interferon and ribavirin (Peg-IFN/RBV) improved the sustained virological response (SVR) achieved with Peg-IFN/RBV in hepatitis C virus genotype 4 (HCV-4)-monoinfected patients. There are no data currently on the efficacy of Peg-IFN/RBV plus NTZ for human immunodeficiency virus (HIV)/HCV-4 coinfection. Therefore, the objectives of this clinical trial were to assess the efficacy and to evaluate the safety of Peg-IFN/RBV plus NTZ in HIV/HCV-4-coinfected patients.

**Patients and Methods:**

This was an open-label, single arm, multicenter phase II pilot clinical trial (NCT01529073) enrolling HIV-infected individuals with HCV-4 chronic infection, naïve to HCV therapy. Patients were treated with NTZ 500 mg bid for 4 weeks, followed by NTZ 500 mg bid plus Peg-IFN alpha-2b 1.5 μg/kg/week plus weight-adjusted RBV during 48 weeks. Analyses were done by intention-to-treat (ITT, missing = failure). A historical cohort of HIV/HCV-4-infected patients treated with Peg-IFN alpha-2b and RBV at the same area was used as control.

**Results:**

Two (9.5%) of 21 patients included in the trial compared with 5 (21.7%) of 23 patients included in the historical cohort achieved SVR (SVR risk difference, -12.2%; 95% confidence interval, -33.2% to 8.8%; p = 0.416). Virological failure was due to lack of response in 13 (62%) individuals recruited in the trial. Two (9.5%) patients included in the trial and two (9.5%) individuals from the historical cohort discontinued permanently due to adverse events.

**Conclusions:**

No increase in SVR was observed among HIV/HCV-4-coinfected patients receiving Peg-IFN/RBV plus NTZ compared with a historical cohort treated with Peg-IFN/RBV. Interruptions due to adverse events of Peg-IFN/RBV plus NTZ were similar to those of dual therapy.

**Trial Registration:**

ClinicalTrials.gov NCT01529073

## Introduction

Infection by hepatitis C virus genotype 4 (HCV-4) is more common in the Middle East and Northern Africa [1], where it may overlap with the HIV epidemic. It is also found in Southern Europe linked to injection drug use [2], so that HCV-4 is observed in approximately 15%-20% patients coinfected with HIV and HCV in that area [3, 4]. In addition, outbreaks of acute HCV-4 among men who have sex with men (MSM) have been recently reported [5]. In Northern European countries, such as The Netherlands, HCV-4 was imported initially from countries where HCV-4 is endemic, later there has been a local spread due to injection drug use and, more recently, an increasing epidemic among HIV-positive MSM [6]. Thus, HCV-4 infection is mainly found in resource-limited settings, but it is also extending to certain groups at increased risk of HIV in Western countries.

There is little information on the response to treatment for chronic hepatitis C in subjects coinfected by HIV and HCV-4. They seem to be difficult to cure patients, with sustained virological response (SVR) to the combination of pegylated interferon plus ribavirin (Peg-IFN/RBV) between 17% and 28% [3, 7–9]. On the contrary, some direct antiviral drugs against HCV (DAA) have shown high SVR frequencies for treatment-naïve HIV/HCV-4-coinfected patients [10, 11]. However, small groups of them were included in the largest trials assessing sofosbuvir (SOF) plus RBV [10] or SOF plus daclatasvir (DCV) [11] in HIV/HCV coinfection. In addition, very few patients with cirrhosis were included in those studies [10, 11].

Nitazoxanide (NTZ) was a drug originally developed as an anti-parasitic agent that eventually showed activity against HCV [12]. In a clinical trial, subjects not previously exposed to anti-HCV drugs and infected with HCV-4, NTZ combined with Peg-IFN/RBV, with a 12-week lead-in of NTZ alone, showed an SVR frequency significantly higher than Peg-IFN/RBV, 79% vs. 50%, respectively [13]. Subsequently, it was demonstrated that a lead-in with NTZ for 4 weeks was as effective as a lead-in for 12 weeks [14]. It is unknown whether the addition of NTZ to Peg-IFN/RBV may improve the poor SVR to Peg-IFN/RBV of HIV/HCV-4-coinfected patients. For this reason, this clinical trial was aimed at evaluating the SVR to Peg-IFN alfa-2b plus RBV and NTZ in patients coinfected with HIV and HCV-4 never exposed to therapy against HCV. SVR frequency was compared with that observed in a historical cohort of HIV/HCV-4-coinfected patients, naïve to anti-HCV therapy, treated with Peg-IFN alfa-2b plus RBV. In addition, the safety of Peg-IFN plus RBV plus NTZ in HIV/HCV-4-coinfected patients was analyzed.

## Patients and Methods

### Design

This was a phase II single arm clinical trial to evaluate the safety and the efficacy of Peg-IFN alfa-2b plus RBV and NTZ in patients with coinfection with HIV and HCV genotype 4. HIV-infected patients evaluated in the participating centers, between August 2012 and December 2013, were eligible for this trial if they fulfilled the following criteria: 1) Aged 18–65 years; 2) chronic HCV-4 coinfection, defined as HCV-4 infection for ≥6 months documented by prior medical history along with detectable plasma HCV RNA; 3) No prior HCV treatment experience; 4) Stable antiretroviral therapy 24 weeks before starting the study drugs, with undetectable plasma HIV RNA during that period of time. All fertile participants were required to be committed to use two forms of non-hormonal contraception during the study and up to 24 weeks after finishing treatment.

Patients were excluded if they had antiretroviral therapy including didanosine, stavudine, zidovudine or abacavir. The risk of mitochondrial toxicity is increased using stavudine or zidovudine together with RBV. An interaction between abacavir and RBV may reduce the likelihood of response to PegIFN/RBV. Patients with prior or current decompensated cirrhosis (i.e. ascites, hepatic encephalopathy, variceal hemorrhage, spontaneous bacterial peritonitis, hepatorenal syndrome, hepatocarcinoma) were excluded. Individuals with significant liver diseases other than chronic HCV infection, including hepatitis B virus infection, were also excluded. Hepatitis B virus infection was diagnosed by the presence of hepatitis B surface antigen in plasma. The use of methadone or other opiate replacement therapy was not considered as an exclusion criterion.

Patients were evaluated at clinic visits by physical examination, laboratory tests, and reports of clinical adverse events. Visits were scheduled every 4 weeks until 28 weeks of treatment and then every 8 weeks until the end of treatment. After finishing treatment, patients were followed every 12 weeks.

### Treatment regimen

Patients fulfilling all the inclusion criteria, and none of the exclusion criteria, were recruited and assigned to receive NTZ 500 mg every 12 hours for 4 weeks followed by NTZ 500 mg every 12 hours plus Peg-IFN alfa-2b 1.5 μg/kg/week and weight-adjusted RBV (800 mg/day if <60 kg, 1000 mg/day if 60–75 kg, 1200 mg/day if ≥75 kg) for 48 weeks.

Treatment stopping rules were applied at week 12 and 24. All drugs were discontinued if HCV-RNA decreased <2 log_10_ from baseline values at week 12 or if HCV-RNA was detectable at week 24. Null response was defined as a <2 log_10_ HCV RNA decrease at week 12. Partial response was considered as a ≥2 log_10_ HCV RNA decrease at week 12 with detectable HCV RNA at week 24. End of treatment response (EOT) was defined as plasma HCV RNA <10 IU/mL at end of treatment, provided that plasma HCV RNA persisted undetectable at least since week 24 of treatment through the end of the course of therapy. SVR was defined as HCV RNA <10 IU/mL 24 weeks after having achieved EOT. Relapse was defined as the detection of HCV RNA 24 weeks after completing the study medication having achieved plasma HCV RNA <10 IU/mL since at least week 24 of therapy through the end of treatment. Individuals experiencing detectable plasma RNA HCV after reaching undetectable levels while on treatment were considered as patients with breakthrough viremia.

No dose adjustment was allowed for NTZ. Dose reductions of Peg-IFN alfa-2b and RBV for hematological adverse events were permitted following a predefined protocol. The initial dose reduction for Peg-IFN alfa-2b was 1 μg/kg/week. If a further dose reduction was necessary, Peg-IFN alfa-2b was decreased to 0.5 μg/kg/week. RBV dose reductions were performed by 200 mg/day decrements. Peg-IFN dose reduction was recommended for patients with platelet counts <30.000 cells/mL. Individuals with hemoglobin levels 8–10 g/dL were managed with erythropoietin primarily and, only if hemoglobin levels did not increase >10 g/dL, with RBV dose reduction. For patients with hemoglobin <8 g/dL, temporary discontinuation of RBV or RBV dose reduction, both with or without blood transfusion, were permitted. No dose adjustment was recommended for neutropenia.

### Historical cohort

A prospective cohort of 843 HIV/HCV-coinfected patients, naive for anti-HCV therapy, who started Peg-IFN/RBV was followed in 10 Spanish centers from December 2001 to December 2011. Among 126 patients with HIV/HCV-4 coinfection, 23 individuals who received Peg-IFNa-2b 1.5 μg/kg every week plus RBV 800–1200 mg daily with a planned duration of 48 weeks were selected as historical control.

### Laboratory methods

Plasma HCV-RNA load was measured by quantitative real time PCR assays (Cobas TaqMan; Roche Diagnostic Systems Inc., Pleasanton, CA, USA) with a lower limit of detection of 10 IU/mL. HCV genotype was determined using a RT-PCR hybridization assay (Versant HCV Genotype 2.0 LIPA; Siemens, Tarrytown, NY, USA). Variations in rs12979860 were genotyped. Genotyping was carried out using a custom Taqman assay (Applied Biosystems, Foster City, California, USA) on DNA isolated from whole blood samples, following the manufacturer’s instructions, on a Stratagene MX3005 thermocycler using MXpro software (Stratagene, La Jolla, California, USA).

### Evaluation of fibrosis

Liver fibrosis was assessed by means of transient elastometry (FibroScan^™^, Paris, France) in all patients included in the clinical trial. For those recruited into the historical cohort, liver fibrosis was primarily assessed by liver biopsy until transient elastometry became available in the cohort after 2005. Cirrhosis was defined as liver stiffness ≥14.6 kPa or liver fibrosis stage F4 in liver biopsy.

### Statistical analysis

The primary outcome measure was SVR. The secondary outcome measure was the proportion of patients with moderate or severe adverse events according to the World Health Organization classification [15]. For the efficacy end point, the analysis included all patients that were recruited. Patients for whom data were missing, who discontinued therapy due to any reason or who dropped out were classified as not having had SVR.

Sample size estimations were based on previous data on the SVR to Peg-IFN/RBV plus NTZ observed in HCV-monoinfected patients [13, 14]. Approximately 100 patients followed in the participating centers would have fulfilled the inclusion criteria for this study. To reach an accuracy level of 10% for a two-sided 95% confidence interval, assuming a frequency of SVR of 50% and a population size of 100, it was necessary to recruit 49 patients. An interim analysis was planned after recruiting half of the estimated sample size. The expected proportion of relapse was 5%. Thus, 55% [95% confidence interval (95%CI): 40%-69%] patients should have continued therapy until the EOT. The frequency of virological failure due to lack of response at weeks 12 and 24 was higher than expected. Due to this, the recruitment was stopped earlier, before reaching the planned sample size.

Categorical variables are presented as proportions (95% confidence intervals, 95%CI) and continuous variables as median (Q1-Q3). For comparisons between groups, Mann-Whitney-U test and Fisher exact test were used, as appropriate. The statistical analysis was carried out using the SPSS statistical software package release 22.0 (IBM SPSS Inc, Chicago, IL, USA) and the Stata 12.0 package (StataCorp, College Station, TX, USA).

### Ethics Statement

The study was designed and conducted following the Helsinki declaration. This study was approved by the Andalusian Regional Ethics Committee (Autonomous Andalusian Committee for Clinical Trials). All patients provided written informed consent to participate. The retrospective historical cohort study was approved by the Ethics Committee of Hospital Universitario de Valme. For patients included in the historical cohort, clinical records and patient information were anonymized and de-identified prior to inclusion in database and analysis.

## Results

### Characteristics of the patients

**Fig 1:**
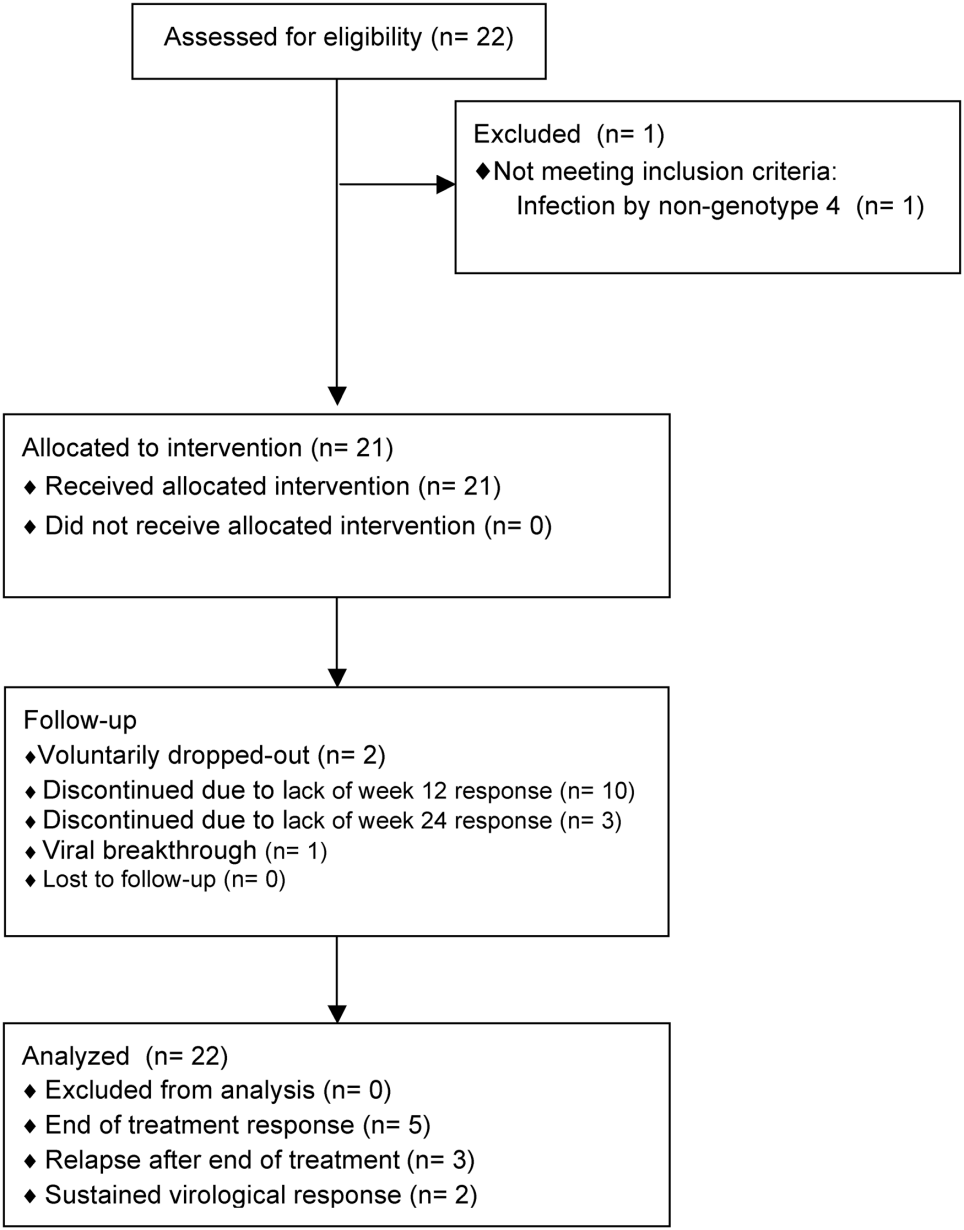
CONSORT 2010 flow diagram.

A total of 22 patients received treatment with NTZ plus Peg-IFN/RBV at 10 centers in Spain. One patient was found to be infected by genotype 1a by sequencing in a subsequent study and, thus, was excluded from this analysis (Fig 1). The main baseline features of the 21 patients included in the trial and of those recruited in the historical cohort are summarized in Table 1. All individuals were on ART and showed undetectable plasma HIV-RNA. Fourteen (67%) patients had advanced fibrosis, i.e. liver stiffness ≥9.5 kPa, and nearly half of the patients showed cirrhosis. Most individuals had baseline plasma HCV-RNA ≥800000 IU/mL and non-CC IL28B genotype (Table 1).

**Table 1:** Baseline characteristics of the patients included in the trial and of a historical cohort.

Characteristic	Study group (N = 21)	Historical cohort (N = 23)	p
Age, years	47 (40–57)	50 (47–51)	0.458
Male gender, n (%)	18 (86)	16 (70)	0.287
Previous use of injecting drugs, n (%)	21 (100)	22 (96)	1
CD4 cell counts, cells/mL	511 (213–1159)	610 (422–776)	0.297
Undetectable plasma HIV RNA, n (%)	21 (100)	19 (81)	0.107
Antiretroviral therapy, n (%)	21 (100)	19 (81)	0.233
Clinical AIDS, n (%)	11 (52)	5 (22)	0.059
HCV RNA, log_10_IU/mL	6.3 (5.3–7.7)	5.6 (5.2–5.8)	0.082
HCV RNA >800.000, n (%)	14 (67)	4 (17)	0.002
IL28B non-CC*, n (%)	16 (76)	9 (90)	0.643
Serum ALT, IU/L	73 (18–177)	56 (42–147)	0.003
Platelet count, 10^3^ cells/mL	165 (70–312)	193 (122–243)	0.077
Cirrhosis^†^	10 (48)	3 (15)	0.046

* Historical cohort: available in 10 patients.

^†^Historical cohort: evaluated by transient elastography or liver biopsy in 19 patients.

### Virological response

**Fig 2:**
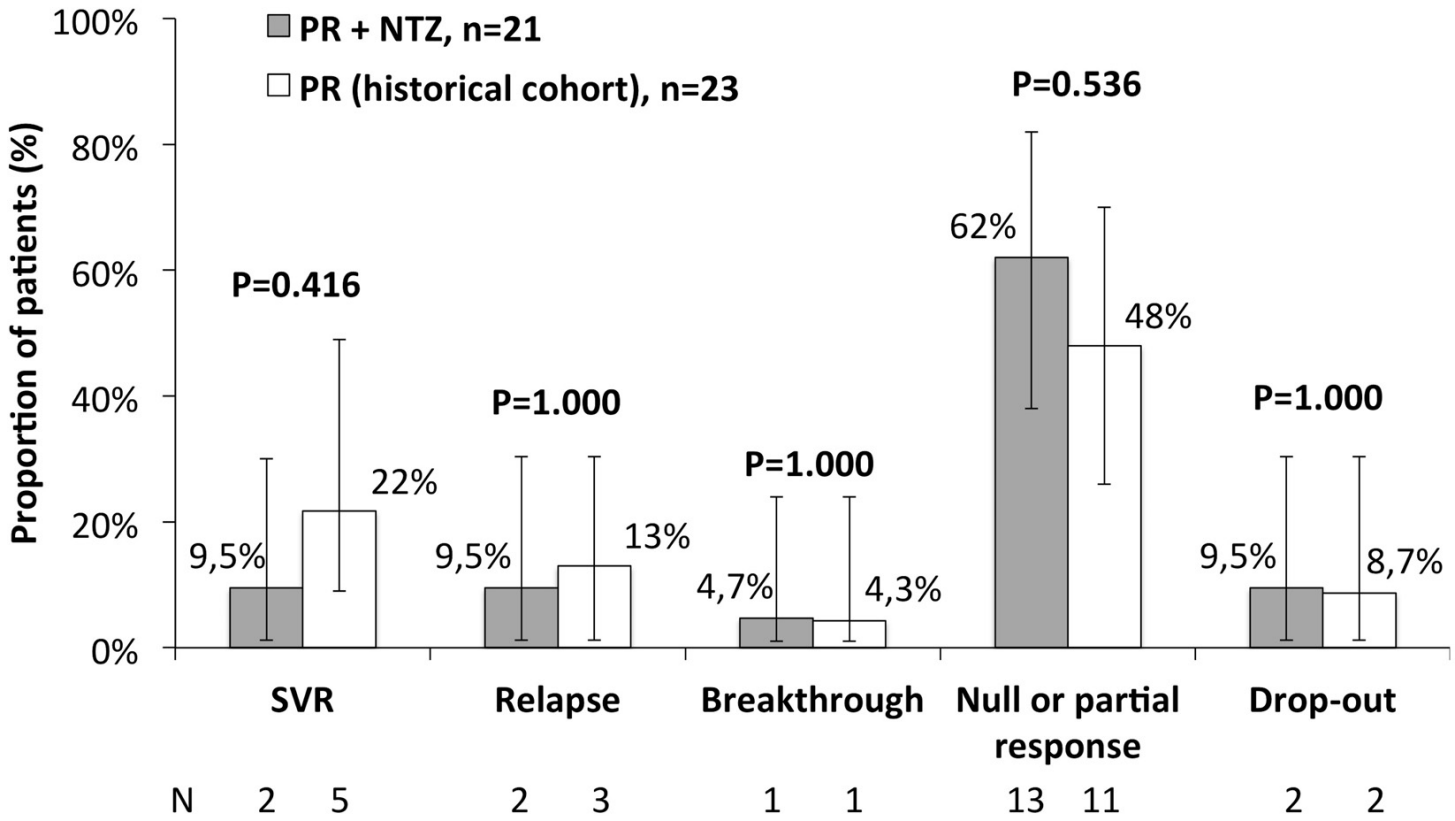
Response to nitazoxanide plus pegylated interferon alpha 2b and ribavirin compared to pegylated interferon alpha 2b and ribavirin (historical control). Null response: <2 log_10_ HCV RNA decrease at week 12. Partial response: ≥2 log_10_ HCV RNA decrease at week 12 with detectable HCV RNA at week 24.

Two (9.5%) of 21 patients included in the trial compared with 5 (21.7%) of 23 patients included in the historical cohort achieved SVR (SVR risk difference, -12.2%; 95% confidence interval, -33.2% to 8.8%; p = 0.416). The median (Q1-Q3) plasma HCV-RNA at baseline, i.e at starting the lead-in phase with NTZ, was 6.34 (5.31–7.66) log_10_ IU/mL and at week 4 of the lead-in phase with NTZ was 6.59 (5.88–7.87) log_10_ IU/mL (p = 0.712). After starting Peg-IFN/RBV plus NTZ, none of the patients reached undetectable plasma HCV-RNA at week 4. At week 12, 10 (47.6%) individuals discontinued therapy because of lack of response. At week 24, three (14.3%) subjects did not reach undetectable plasma HCV-RNA. One (4.7%) patient showed viral breakthrough at week 28. Thus, virological failure was due to lack of response in 13 (62%) individuals. Two (9.5%) individuals experienced viral relapse after the end of treatment. Two (9.5%) individuals voluntarily dropped out. Virological response in the trial compared with the historical cohort is summarized in Fig 2.

### Safety

The most common adverse events were fatigue, anorexia and insomnia (Table 2). Other less frequent side effects were depression, myalgia, headache, itching, diarrhea, rash, irritability, local reaction at the site of injection. One patient suffered bacterial pneumonia. Two (9.5%) patients discontinued permanently due to adverse events, one of them because of symptomatic anemia and the other one due to severe rash. Interruption due to adverse events in the historical cohort was reported in 2 (8.7%) patients. The most common grade 3 or 4 laboratory abnormalities were anemia, neutropenia and thrombocytopenia. RBV dose was not decreased in any patient and erythropoietin was used in 2 (9.5%) individuals because of anemia. The dose of Peg-IFN was not reduced in any patient with neutropenia.

**Table 2:** Adverse effects experienced by the study patients.

Event, n (%)	Study group (n = 21)
Any adverse effect	16 (76)
Treatment-related adverse event*	14 (67)
Serious adverse event^†^	3 (14)
Treatment-related serious adverse event	2 (9.5)
Discontinuation due to adverse events	2 (9.5)
Adverse events observed in ≥25%	
Fatigue	9 (41)
Pyrexia	9 (41)
Anorexia	9 (41)
Myalgia	8 (38)
Headache	8 (38)
Nausea	7 (33)
Insomnia	6 (29)
Laboratory adverse events	
Hemoglobin level ≤10 g/dL	4 (19)
Hemoglobin level ≤8.5 g/dL	1 (4.8)
Platelet count <50.000 cells/mL	2 (9.5)
Neutrophil count <1000 cells/mL	8 (38)
Neutrophil count <500 cells/mL	0

*Events judged to be possibly or probably related to the study treatment;

^†^Serious adverse events were defined as fatal or life-threatening events and as those that required or prolonged hospitalization, resulted in persistent or clinically significant disability or congenital anomaly, or required a medical or surgical intervention to preclude permanent impairment of body function or structure.

## Discussion

In this pilot clinical trial, no increase in SVR was observed among HIV/HCV-4-coinfected patients receiving Peg-IFN/RBV plus NTZ compared with a historical cohort treated with Peg-IFN/RBV. Interruptions due to adverse events of Peg-IFN/RBV plus NTZ were similar to those of Peg-IFN/RBV.

This is the first clinical trial assessing Peg-IFN/RBV plus NTZ among HIV/HCV-4-coinfected patients. This trial did not confirm the increased SVR of Peg-IFN/RBV plus NTZ found in HCV-4-monoinfected patients. In previous trials in Egyptian HCV-4-infected patients, the SVR frequency of those receiving Peg-IFN/RBV plus NTZ was close to 80%, i.e. 30% greater than that of individuals assigned to Peg-IFN/RBV [13, 14]. On the contrary, we observed a far lower SVR to Peg-IFN/RBV plus NTZ for HIV/HCV-4 coinfection, without differences with a historical control receiving Peg-IFN/RBV. There is no clear explanation for these contrasting results. One possible reason is that those trials were designed before the effect of IL28B on response to therapy was described. Among patients carrying HCV-4 with or without HIV coinfection, IL28B variations exert a dramatic influence on SVR [8, 9, 15], even stronger than in HCV-1 infection. Differences in IL28B in those initial trials might partly explain the contrasting results between studies. In addition, individuals of Egyptian ancestry seem to achieve better frequency of SVR to therapy against HCV-4 [13] than subjects of other ancestries [15, 16]. Furthermore, HIV infection might also be another reason for the lack of effect of the addition of NTZ to Peg/RBV on SVR.

Our results are in agreement with those reported in the ACTG A5269 [17]. That was a pilot clinical trial to assess the efficacy of Peg-IFN alpha 2a and RBV plus NTZ for HIV and genotype 1 (HCV-1)-coinfected treatment-naïve patients. The addition of NTZ to Peg-IFN alpha 2a plus RBV did not improve SVR among HIV/HCV-1-coinfected subjects naïve to HCV therapy in comparison to historical control data [17]. However, the frequency of virological response at week 12 of Peg-IFN alpha 2a and RBV plus NTZ was higher than those of HIV/HCV-1-coinfected naïve patients treated with Peg-IFN alpha 2a and RBV in the ACTG A5178, a previously completed trial used as historical control [17]. This initial advantage for NTZ plus Peg-IFN/RBV was not translated into a higher HCV clearance. In our study, the main reason for lack of SVR was on treatment virological failure. Indeed, nearly half of the patients showed null response at week 12. Thus, we did not find any benefit throughout treatment from the addition of NTZ to Peg-IFN/RBV among HIV/HCV-4-coinfected patients. This disparity between these trials might be due to the different HCV genotypes tested, HCV-1 vs. HCV-4. In addition, it is also possible that the use of Peg-IFN alpha 2a in the ACTG A5269 and of Peg-IFN alpha 2b in the present trial might have influenced those differences. In this regard, treatment of HCV-4 infection with Peg-IFN alpha 2a achieves a higher proportion of response at week 12 than Peg-IFN alpha 2b with similar final SVR frequency [18].

HCV-4 is not infrequently found in HIV/HCV coinfection [3, 4, 7–9]. However, very few data is available on the treatment of HCV-4 in HIV-coinfected patients. Several clinical trials have explored the efficacy of Peg-IFN/RBV plus a DAA in HCV-4 monoinfection [19–21]. Regarding IFN-free regimens, there is data supporting the use of ritonavir-boosted paritaprevir plus ombitasvir plus RBV for HCV-4-monoinfected patients [22]. In a small group of HIV/HCV-4-coinfected patients, promising responses were achieved with SOF plus RBV for 24 weeks [23]. There is anecdotal information on SOF plus DCV in one naïve and two treatment-experienced HIV/HCV-4-coinfected individuals [11]. Theoretically, combinations including SOF plus SMV or DCV plus SMV should be highly active against HCV-4, but currently there is no data available for those regimens. Thus, unfortunately, it is presently unclear what the best treatment options for HCV-4 in HIV coinfection are.

Some limitations of this study are due to the design as pilot clinical trial. There was no randomized comparison with a control group, because this was an exploratory trial. The sample size was small and calculated using HCV-4 monoinfection data [13, 14]. The difference in SVR between groups of HCV-4-monoinfected patients receiving Peg-IFN/RBV with or without NTZ was assumed as potentially achievable by HIV/HCV-4-coinfected patients. The final sample size of the study was even smaller due to an early termination warranted after an efficacy interim analysis. However, given the trends observed, it is highly unlikely that a larger sample size would have changed the conclusions reached herein. Another limitation could be related with the differences in baseline variables between the study group and the historical cohort. Particularly, variables that may potentially adversely influence SVR were more frequent in the study group, such as the proportion of patients with high plasma HCV RNA levels or the frequency of cirrhosis. However, we had previously shown that the only factor predictive of SVR to Peg-IFN/RBV among HIV/HCV-4-coinfected patients is IL28B (ref. 8, Mira et al. *AIDS* 2012,**26**:1721–1724). There were no significant differences in the proportion of IL28B CC variation between the NTZ treatment group and the historical cohort.

It has been suggested that combinations of NTZ or other thiazolides with DAA for treating chronic hepatitis C may be a path for future development of that drug [24]. However, we failed to find any data supporting that NTZ should be considered a possible agent to be included in drug combinations to treat HCV-4 in HIV coinfection. HCV treatment is a rapidly evolving field with very few data supporting some potential IFN-free treatment options for HCV-4 infection in patients living with HIV. Studies aimed at assessing combinations against HCV-4 are needed before any assumption of antiviral activity is made in the specific setting of HIV infection.

## Supporting Information

S1 Protocol Original Study Protocol (DOCX)

S2 Protocol English translation of the Study Protocol. (DOCX)

S1 TREND Checklist TREND checklist. (PDF)
