# Supplementary material for: Low Efficacy of Pegylated Interferon plus Ribavirin plus Nitazoxanide for HCV Genotype 4 and HIV Coinfection
Source: PLoS One. 2015 Dec 7;10(12):e0143492. doi: 10.1371/journal.pone.0143492 (PMC4671604; doi:10.1371/journal.pone.0143492)
Supplement: S1 Protocol — (DOCX) [file pone.0143492.s001.docx]

**ENSAYO CLÍNICO PILOTO, FASE II PARA EVALUAR LA ACTIVIDAD ANTIVÍRICA DE LA COMBINACIÓN DE INTERFERÓN PEGILADO MÁS RIBAVIRINA MÁS NITAZOXANIDA EN INDIVIDUOS CON HEPATITIS CRÓNICA DEBIDA A GENOTIPO 4 DEL VHC Y COINFECTADOS POR VIH**

*Código del protocolo: NTZSPA001*

*Código EudraCT : 2010-024336-42*

*Código ClinTrials.gov: NCT01529073*

**PROMOTOR INVESTIGADORES COORDINADOR PRINCIPALES**

Dr. Juan Macías Sánchez Dr. Ramón Morillo Verdugo

UGC de Enfermedades Infecciosas UGC de Farmacia

Hospital Universitario de Valme Hospital Universitario de Valme

Sevilla  Sevilla

Dr. Juan Macías Sánchez

UGC de Enfermedades Infecciosas

Hospital Universitario de Valme

Sevilla

#

# TÍTULO Y VERSIÓN DEL PROTOCOLO

ENSAYO CLÍNICO PILOTO, FASE II PARA EVALUAR LA ACTIVIDAD ANTIVÍRICA DE LA COMBINACIÓN DE INTERFERÓN PEGILADO MÁS RIBAVIRINA MÁS NITAZOXANIDA EN INDIVIDUOS CON HEPATITIS CRÓNICA DEBIDA A GENOTIPO 4 DEL VHC Y COINFECTADOS POR VIH

Título corto: PROTOCOLO ESTUDIO NITAZOXANIDA EN COINFECTADOS POR VIH Y VHC-4

Versión 1ª, revisión 3ª: 08/06/2011

# RESPONSABLES DEL ESTUDIO

Juan Macías Sánchez

UGC de Enfermedades Infecciosas

Hospital Universitario de Valme

Avda. de Bellavista s/n

41014 – Sevilla

Telf.: 955015757

Fax: 955015461

Ramón Morillo Verdugo

UGC de Farmacia

Avda. de Bellavista s/n

41014 – Sevilla

Telf.: 955015467

Fax: 955015461

# PROMOTOR

Juan Macías Sánchez

UGC de Enfermedades Infecciosas

Hospital Universitario de Valme

Avda. de Bellavista s/n

41014 – Sevilla

Telf.: 955015757

Fax: 955015461

# RESUMEN

## Identificación del promotor y dirección

Juan Macías Sánchez

UGC de Enfermedades Infecciosas

Hospital Universitario de Valme

Avda. de Bellavista s/n

41014 – Sevilla

Telf.: 955015757

Fax: 955015461

## Título del estudio

ENSAYO CLÍNICO PILOTO, FASE II PARA EVALUAR LA ACTIVIDAD ANTIVÍRICA DE LA COMBINACIÓN DE INTERFERÓN PEGILADO MÁS RIBAVIRINA MÁS NITAZOXANIDA EN INDIVIDUOS CON HEPATITIS CRÓNICA DEBIDA A GENOTIPO 4 DEL VHC Y COINFECTADOS POR VIH

## Código del protocolo

*Código del protocolo: NTZSPA001*

*Código EudraCT : 2010-024336-42*

## Investigador coordinador, monitor y dirección

Juan Macías Sánchez

UGC de Enfermedades Infecciosas

Hospital Universitario de Valme

Avda. de Bellavista s/n

41014 – Sevilla

Telf.: 955015757

Fax: 955015461

Ramón Morillo Verdugo

UGC de Farmacia

Avda. de Bellavista s/n

41014 – Sevilla

Telf.: 955015467

Fax: 955015461

## Tipo de centros donde se prevé realizar el estudio

Nueve centros hospitalarios españoles donde se realiza seguimiento de pacientes coinfectados por VIH/VHC.

## CEIC que lo evalúa

El estudio será sometido a la evaluación del Comité Autonómico de Ensayos Clínicos de la Comunidad Autónoma de Andalucía y de Madrid.

## Objetivo principal

Evaluar la tasa de RVS del tratamiento con Peg-IFN alfa-2b más RBV más NTZ en pacientes coinfectados por VIH y por el genotipo 4 del VHC, tanto no expuestos nunca a tratamiento como con fracaso a un tratamiento estándar con Peg-IFN más RBV previo, y compararla con la tasa de RVS obtenida en estos pacientes con Peg-IFN más RBV en una cohorte histórica.

## Diseño

Ensayo clínico piloto no controlado para evaluar eficacia y seguridad (fase II).

## Enfermedad o trastorno en estudio

Coinfección por VIH y genotipo 4 del VHC.

## Datos de los medicamentos objeto de estudio

Nitazoxanida 500 mg cada 12 horas durante 4 semanas, seguido por nitazoxanida 500 mg cada 12 horas más interferón pegilado alfa-2b 1.5 μg/Kg/semana más ribavirina ajustada según peso durante 48 semanas.

## Población en estudio y número total de sujetos

Pacientes infectados por el VIH-1 con hepatitis crónica por el genotipo 4 del VHC que cumplan los criterios de selección.

Número de pacientes a incluir en el estudio: 45.

## Calendario

Se prevé el siguiente calendario (en total el estudio durará 12 meses desde la inclusión del primer al último paciente más 18 meses de seguimiento). Los tiempos descritos en este apartado pueden verse modificados por los plazos del proceso administrativo de puesta en marcha del estudio:

- Inicio del estudio: 01/03/2012
- Periodo de inclusión: 01/03/2012-30/06/2012
- Periodo de seguimiento: 01/03/2012-31/12/2013.
- Recogida de datos: 01/03/2012-31/12/2013.
- Análisis estadístico final: 01/01/2014-30/01/2014.

## Fuente de financiación

El promotor, Dr. Juan Macías, garantiza la no interferencia en los procesos de selección de los casos, análisis de la información y/o presentación de resultados, o cualquier otro proceso que pueda incidir en los resultados del estudio.

La financiación de este estudio procederá de la convocatoria 2010 de Ayudas para el Fomento de la Investigación Clínica Independiente del Ministerio de Sanidad, Política Social e Igualdad (expediente EC10-187). La financiación será en todo caso independiente de los resultados del estudio.

# INDICE

1 TÍTULO Y VERSIÓN DEL PROTOCOLO 2

2 RESPONSABLES DEL ESTUDIO 2

3 PROMOTOR 2

4 RESUMEN 3

4.1 Identificación del promotor y dirección 3

4.2 Título del estudio 3

4.3 Código del protocolo 3

4.4 Investigador coordinador, monitor y dirección 3

4.5 Tipo de centros donde se prevé realizar el estudio 4

4.6 CEIC que lo evalúa 4

4.7 Objetivo principal 4

4.8 Diseño 4

4.9 Enfermedad o trastorno en estudio 4

4.10 Datos de los medicamentos objeto de estudio 4

4.11 Población en estudio y número total de sujetos 5

4.12 Calendario 5

4.13 Fuente de financiación 5

5 INDICE 6

6 PLAN DE TRABAJO 9

7 OBJETIVOS 11

7.1 Objetivo principal 11

7.2 Objetivos secundarios 11

8 REVISIÓN DE LA LITERATURA 11

8.1 Antecedentes 11

8.2 Justificación del estudio 14

9 METODOS 15

9.1 Diseño del estudio 15

9.2 Población de estudio 15

9.2.1 Criterios de selección 15

9.2.2 Criterios de exclusión: 16

9.2.3 Criterios de retirada: 17

9.3 Descripción del tratamiento y exposición 18

9.4 Fuente de información y ámbito 18

9.5 Variables del estudio 18

9.5.1 Variable principal 18

9.5.2 Variables secundarias 18

9.5.3 Variables explicativas o covariables 19

9.6 Justificación del tamaño muestral 19

9.7 Métodos para la obtención de datos 20

9.8 Manejo de los datos 20

9.9 Análisis de los datos 20

9.9.1 Aspectos específicos del análisis estadístico 21

10 MEDICACIÓN DEL ESTUDIO 23

10.1 Fármacos en investigación 23

10.2 Dispensación de los fármacos del estudio 23

10.3 Recepción, almacenamiento y control de los fármacos del estudio 24

10.4 Trazabilidad: 25

10.4.1 Trazabilidad medicación dispensada 25

10.5 Ajuste de la dosis y suspensión del tratamiento 27

10.5.1 Nitazoxanida 27

10.5.2 Tratamiento de referencia 27

10.5.3 Tratamientos concomitantes no permitidos 30

10.5.4 Terminación del estudio y tratamiento post-estudio 32

11 SEGURIDAD 34

11.1 Exploración física 34

11.2 Constantes vitales 34

11.3 Estatura y peso corporal 35

11.4 Evaluaciones de laboratorio 35

11.5 Pruebas de laboratorio 35

11.6 Embarazo y evaluación de la fertilidad 36

11.7 Otras pruebas para evaluar la seguridad 38

12 ACONTECIMIENTOS ADVERSOS 40

12.1 Intensidad 41

12.2 Relación con el o los fármacos en investigación 41

12.3 Reacción adversa 43

12.4 Notificación de los acontecimientos adversos graves 44

12.5 Embarazos 46

12.6 Comité para la Vigilancia de Datos y Seguridad 47

13 ASPECTOS ÉTICOS / PROTECCIÓN DE LOS SUJETOS PARTICIPANTES 49

13.1 Evaluación beneficio-riesgo 49

13.2 Hoja de información y formulario de consentimiento 49

13.3 Confidencialidad de los datos 50

13.4 Responsabilidades del investigador y del CEIC 51

14 PLANES DE DIFUSIÓN DE LOS RESULTADOS 53

15 FINANCIACIÓN 55

16 MODIFICACIONES DEL PROTOCOLO 56

16.1 Adherencia al protocolo 56

17 CONSIDERACIONES PRÁCTICAS 56

a. Informes de seguimiento y final 56

b. Difusión de los resultados 57

c. Responsabilidades del promotor 57

d. Responsabilidades del investigador 58

e. Responsabilidades del investigador coordinador 59

Anexo 1. CUADERNO DE RECOGIDA DE DATOS 60

Anexo 2. REGISTRO DE DISPENSACION DE PACIENTES 73

Anexo A3. NOTIFICACIÓN DE RAGI A NITAZOXANIDA. 74

Anexo 4. NOTIFICACIÓN DE RAGI A INTERFERÓN PEGILADO ALFA 2-B Ó RIBAVIRINA. 75

Anexo 5. FORMULARIO DE NOTIFICACIÓN DE REACCIÓN ADVERSA GRAVE E INESPERADA. 76

Anexo 6. COMPROMISO DEL INVESTIGADOR COORDINADOR 78

Anexo 7. COMPROMISO DEL INVESTIGADOR 79

Anexo 8. CONFORMIDAD DEL CEIC 80

Anexo 9. FICHA TÉCNICA DEL MEDICAMENTO INVESTIGADO 83

Anexo 10. HOJA DE INFORMACIÓN AL PACIENTE 84

Anexo 11. FORMULARIO DE CONSENTIMIENTO INFORMADO 86

Anexo 13. MEMORIA ECONOMICA 88

Anexo 14. BIBLIOGRAFÍA 89

# PLAN DE TRABAJO

Una vez obtenida la aprobación del estudio por el CEIC y de las Autoridades Sanitarias de las Comunidades Autónomas participantes, se procederá al inicio del mismo en los centros participantes, tras informar a las gerencias de los mismos de la realización del estudio. Los investigadores participantes serán los únicos responsables de la inclusión de los pacientes en el estudio tras confirmar que cumplen los criterios de selección establecidos en el mismo. A cada paciente que se le invite a participar en el estudio se le informará acerca del mismo tanto de forma verbal como por escrito, proporcionándole un documento denominado “Hoja de información al paciente” (Anexo 7). No se incluirá a ningún paciente en el estudio hasta que éste haya sido debidamente informado por el investigador y haya otorgado libremente su consentimiento a participar en el mismo, bien por escrito o de forma oral ante testigos independientes del equipo investigador. Tras la aceptación por parte del paciente, se procederá al inicio de la recogida de los datos mediante la cumplimentación de una base de datos electrónica que será proporcionada por el promotor, destinada a registrar la información disponible en la historia clínica del paciente así como los datos facilitados por él mismo. La inclusión de enfermos se terminará cuando se alcance el tamaño muestral previsto. Se describen a continuación las visitas previstas y las variables a recoger en cada una de ellas:

| **Evaluación** | **Semanas (Visitas)** | | | | | | | | | | | | | |
| --- | --- | --- | --- | --- | --- | --- | --- | --- | --- | --- | --- | --- | --- | --- |
|  | **Cribado**  **(V1)** | **0 (V2)** | **4 (V3)** | **8 (V4)** | **12 (V5)** | **16 (V6)** | **20 (V7)** | **24 (V8)** | **28 (V9)** | **36 (V10)** | **44 (V11)** | **52 (V12)** | **64 (V13)** | **76 (V14)** |
| Procedimientos del estudio |  |  |  |  |  |  |  |  |  |  |  |  |  |  |
| Consentimiento informado |  | x |  |  |  |  |  |  |  |  |  |  |  |  |
| Historia médica y examen físico | x | x | x |  | x |  |  | x |  | x |  | x |  | × |
| Signos vitales | x | x | x | x | x | x | x | x | x | x | × | x | × | × |
| Intervención |  |  |  |  |  |  |  |  |  |  |  |  |  |  |
| NTZ monoterapia |  | x | x |  |  |  |  |  |  |  |  |  |  |  |
| NTZ+PegIFN+RBV |  |  | x | x | x | x | x | x | x | x | x | x |  |  |
| Evaluaciones analíticas |  |  |  |  |  |  |  |  |  |  |  |  |  |  |
| Test de embarazo (orina o suero) | x | x | x | x | x | x | x | x | x | x | x | x | x | x |
| ARN de VIH plasmático | x | x |  |  | x |  |  | x |  | x |  | x |  | x |
| Recuento de células CD4+ y CD8+ | x | x |  |  | x |  |  | x |  | x |  | x |  | × |
| Hematología | x | x | x | x | x | x | x | x | x | x | × | × | × | × |
| Bioquímica | x | x | x | x | x | x | x | x | x | x | × | × | × | × |
| Perfil lipídico |  | x | x |  | x |  |  | x |  | x |  | x |  | x |
| Análisis de orina | x | x | x | x | x | x | x | x | x | x | × | × | × | × |
| Test de función tiroidea | x | x | x | x | x | x | x | x | x | x | x | x | x | × |
| HOMA |  | x |  |  |  |  |  |  |  |  |  | x |  | x |
| Polimorfismos de IL28B |  | x |  |  |  |  |  |  |  |  |  |  |  |  |
| Pruebas de imagen y otras |  |  |  |  |  |  |  |  |  |  |  |  |  |  |
| Fibrosis hepática por medio de elastometría transitoria |  | x |  |  |  |  |  |  |  |  |  |  |  | x |
| ECG |  | x |  |  | x |  |  | x |  |  |  | x |  | x |
| Medidas de eficacia |  |  |  |  |  |  |  |  |  |  |  |  |  |  |
| ARN de VHC plasmático | x | x | x | x | x | x | x | x | x | x | x | x | x | × |
| Evaluación cumplimentación tratamiento |  |  | x | x | x | x | x | x | x | x | × | × |  |  |
| Medidas de seguridad |  |  |  |  |  |  |  |  |  |  |  |  |  |  |
| Efectos adversos |  |  | x | x | x | x | x | x | x | x | × | × |  |  |
| Medicación concomitante | x | x | x | x | x | x | x | x | x | x | × | × | × | × |

# OBJETIVOS

## Objetivo principal

Evaluar la tasa de RVS del tratamiento con Peg-IFN alfa-2b más RBV más NTZ en pacientes coinfectados por VIH y por el genotipo 4 del VHC, tanto no expuestos nunca a tratamiento como con fracaso a un tratamiento estándar con Peg-IFN más RBV previo, y compararla con la tasa de RVS obtenida en estos pacientes con Peg-IFN más RBV en una cohorte histórica.

## Objetivos secundarios

- Evaluar la actividad virológica en la semana 4 y 12 de Peg-IFN alfa-2b más RBV más NTZ en pacientes coinfectados por VIH y por el genotipo 4 del VHC, tanto en no expuestos a terapia anteriormente y como en aquellos sin RVS a un tratamiento previo con Peg-IFN más RBV.
- Analizar la seguridad de la combinación Peg-IFN alfa-2b más RBV más NTZ en pacientes coinfectados por VIH y por el genotipo 4 del VHC, en no expuestos a terapia anteriormente y en sujetos sin RVS a un tratamiento previo con Peg-IFN más RBV.

# REVISIÓN DE LA LITERATURA

## Antecedentes

La cirrosis hepática es actualmente una de las causas principales de morbilidad y mortalidad en los pacientes coinfectados por el virus de la inmunodeficiencia humana (VIH) y el virus de la hepatitis C (VHC) en España (1). La exposición a tratamientos antirretrovirales eficaces (TAR) modifica favorablemente el curso de la hepatitis crónica C en el contexto de la infección por VIH (2). Sin embargo, después de iniciar el TAR, la historia natural acelerada de la hepatitis crónica C en la infección por VIH posiblemente no retorna al curso relativamente indolente del paciente monoinfectado por VHC. De hecho, la proporción de pacientes coinfectados que progresa en espacios breves de tiempo en los estudios basados en dos biopsias hepáticas es muy elevada, hasta una cuarta parte presenta aumentos en el estadio de fibrosis (3). Esta progresión rápida se observa en poblaciones de sujetos mayoritariamente expuestos a TAR eficaz (3). Por ello, aunque el TAR puede enlentecer la progresión de la hepatopatía por VHC incluso en situación de enfermedad hepática terminal (4), es necesario abordar el tratamiento de la infección por VHC sin retraso en la mayoría de los pacientes coinfectados por VIH/VHC.

La medida más eficaz para evitar la progresión de la hepatitis crónica C en los sujetos coinfectados por VIH es el tratamiento del VHC (1,5). Los individuos coinfectados que alcanzan una respuesta viral sostenida (RVS) al tratamiento combinado con interferón pegilado (Peg-IFN) más ribavirina (RBV) presentan una probabilidad menor de descompensaciones hepáticas y de muerte por hepatopatía que aquellos que no consiguen una RVS (5). Sin embargo, la proporción de pacientes coinfectados por VIH/VHC que logran una RVS con el tratamiento anti-VHC es baja en comparación con los sujetos monoinfectados por VHC (6-10). En ensayos clínicos, entre 27% y 50% de los coinfectados presentan RVS (6-8). En condiciones de la vida real, en estudios de cohortes de coinfectados por VIH/VHC, se ha observado RVS globalmente entre 31% y 37% de los pacientes (9,10). Es posible mejorar la tasa de RVS en los sujetos coinfectados expuestos a TAR si se seleccionan como núcleos(t)idos tenofovir y emtricitabina o lamivudina (9,11). Sin embargo, incluso en esta situación óptima, no presentan RVS un 55% de los pacientes (11).

El VHC es un virus ARN caracterizado por una importante heterogeneidad genética. Existen, al menos, seis genotipos principales del VHC. Cada genotipo difiere de los otros en un 30-35% de su secuencia de nucleótidos. Además, la distribución geográfica y la respuesta al tratamiento de cada genotipo es única. Los genotipos 1, 2 y 3 son comunes en los EE.UU. y Europa y, por ello, han sido objeto de interés e investigación. El genotipo 4 del VHC (VHC-4) es uno de los poco estudiados, a pesar de causar el 20% de los 170 millones de casos de hepatitis crónica C mundialmente. Es común sobre todo en Oriente Medio y África, pero en los últimos 20 años se ha extendido a áreas como Grecia, Italia, Francia y España. En el sur de Europa se ha vinculado al uso de drogas parenterales, por lo que en pacientes coinfectados por VHC de nuestra área no es un genotipo raro. Alrededor del 15% de los sujetos coinfectados por VIH/VHC en España son portadores de un VHC-4 (12). Disponemos de escasa información sobre la respuesta al tratamiento con Peg-IFN más RBV de los sujetos coinfectados por VIH y VHC-4. En nuestra área, se comportan como pacientes difíciles de tratar, con tasas de RVS a la combinación de Peg-IFN más RBV entre 17% y 28% (13, 14).

La nitazoxanida (NTZ) es un fármaco inicialmente desarrollado como antiparasitario que ha mostrado actividad anti-VHC (15). En modelos de replicones del VHC, se demostró que la actividad inhibitoria de la replicación del VHC dependía de la activación de proteínas celulares implicadas en la respuesta mediada por interferón (15). En un ensayo clínico en sujetos no expuestos anteriormente a fármacos anti-VHC e infectados por VHC-4, el brazo que incluyó un pretratamiento durante 12 semanas con NTZ seguido por un ciclo de tratamiento con NTZ más Peg-IFN más RBV mostró tasas de RVS significativamente mayores que el brazo que recibió tratamiento estándar, 79% vs. 50%, respectivamente (16). Posteriormente, se ha probado que un pretratamiento con monoterapia con NTZ durante 4 semanas es igualmente efectivo que el pretratamiento durante 12 semanas (17). La experiencia en sujetos infectados por VHC-4 y que fracasaron, incluyendo ausencia de respuesta y recidiva, al Peg-IFN más RBV es limitada (18). Los pacientes infectados por VHC-4, sin RVS a un primer tratamiento con Peg-IFN más RBV, que recibieron NTZ más el estándar de tratamiento presentaron tasas de RVS superiores a las de los sujetos retratados con Peg-IFN más RBV, 25% vs. 8%, respectivamente (18).

A corto plazo, un volumen muy importante de pacientes coinfectados por VIH/VHC sin RVS a Peg-IFN más RBV podría presentar descompensaciones de su hepatopatía, situación en la que las opciones terapéuticas son muy limitadas. Por otra parte, actualmente los pacientes infectados por VHC-4 sin RVS al Peg-IFN más RBV no tienen posibilidades reales de ser retratados con las nuevas moléculas directamente activas frente al VHC (DAV) que se están desarrollando. Las DAV se han evaluado en modelos de laboratorio y en estudios clínicos diseñados para inhibir aislados del genotipo 1 (19). Por ello, las DAV dirigidas hacia regiones más variables del VHC tienen más posibilidades de no ser eficaces en genotipos distintos del 1. Entre ellos se encuentran los inhibidores de la proteasa NS3 del VHC, las DAV más avanzadas en su desarrollo. Concretamente, telaprevir presenta una actividad antiviral prácticamente ausente frente a los genotipos 3 (20) y 4 (20). Boceprevir presenta una actividad antiviral muy reducida frente a los genotipos 2 y 3 (19). Es esperable un comportamiento similar para los inhibidores no-análogos de los nucleósidos de la polimerasa NS5B (19). Por estos motivos, la única alternativa terapéutica que permitiría retratar a tiempo a los pacientes infectados por VHC-4 con un fracaso a un primer tratamiento con Peg-IFN más RBV, especialmente a los coinfectados con fibrosis avanzada, pasaría por el uso de NTZ.

## Justificación del estudio

Se desconoce si la adición de NTZ al tratamiento estándar actual podría mejorar las pobres tasas de RVS de los sujetos coinfectados por VIH y VHC-4, y si, además, puede incrementar eficacia del retratamiento de pacientes que no han presentado RVS con terapia estándar. Además, no existe información sobre la seguridad de la adición de NTZ al tratamiento estándar con Peg-IFN más RBV en pacientes infectados por VIH. Estos pacientes sufren con mayor frecuencia efectos adversos derivados del Peg-IFN más RBV, en parte porque la mayoría de ellos está con TAR. El TAR puede influenciar la frecuencia y gravedad de los efectos adversos del tratamiento frente al VHC. Por estas razones, se deberían planear estudios que examinen la eficacia y seguridad de Peg-IFN más RBV más NTZ en pacientes coinfectados por VIH/VHC-4.

# METODOS

## Diseño del estudio

Ensayo clínico piloto no controlado para evaluar eficacia y seguridad (fase II).

Brazos del estudio:

Brazo experimental: NTZ 500 mg cada 12 horas durante 4 semanas, seguido por NTZ 500 mg cada 12 horas más Peg-IFN alfa-2b 1.5 μg/Kg/semana más RBV ajustada según peso durante 48 semanas.

Ámbito del estudio:

Unidades de Enfermedades Infecciosas de 9 hospitales pertenecientes al Sistema Sanitario Público, ocho andaluces y uno madrileño.

## Población de estudio

### ***Criterios de selección***

- **Criterios de inclusión:**

1. Infección por VIH.
2. Infección por el genotipo 4 del VHC.
3. No tratamiento previo con ningún tipo de interferón o ausencia de respuesta a un primer tratamiento previo con Peg-IFN más RBV. La falta de respuesta incluirá tanto a los sujetos no respondedores, como a los que mostraron recidivas.
4. TAR estable 24 semanas antes de comenzar el estudio, con ARN plasmático del VIH indetectable durante ese periodo de tiempo.
5. Compromiso de usar dos métodos anticonceptivos no hormonales durante el estudio y hasta 24 semanas después del mismo.
6. Aceptación de dar el consentimiento informado por escrito para participar en el ensayo (Anexo 1).

### ***Criterios de exclusión:***

1. Inclusión de didanosina, estavudina, zidovudina o abacavir en el TAR.
2. Cirrosis descompensada.
3. Presencia de otras enfermedades hepáticas significativas, incluidas hepatitis crónica o aguda por virus de la hepatitis B, hepatitis aguda por virus de la hepatitis A, hemocromatosis o déficit de alfa-1 antitripsina.
4. Embarazo o lactancia.
5. Hombres con planes de embarazo con sus parejas durante el estudio y hasta 24 semanas después de terminado el tratamiento.
6. Depresión activa o mal controlada, otras enfermedades psiquiátricas, o cualquier enfermedad a lo largo del año previo que pueda, en opinión del investigador, impedir la participación en el estudio.
7. Intento de suicido previo.
8. Enfermedad tiroidea activa o mal controlada con tratamiento.
9. Enfermedades autoinmunes previas como enfermedad inflamatoria intestinal, psosiasis grave, o artritis reumatoide, que pudieran exacerbarse con el interferón.
10. Tratamiento quimioterápico o inmunomodulatorio 24 semanas antes de comenzar el estudio.
11. Enfermedad grave, incluyendo cáncer o enfermedad coronaria no estable, 24 semanas antes de comenzar el estudio.
12. Cualquier enfermedad o problema crónico que, en opinión del investigador, puede impedir completar el estudio.
13. Presencia de enfermedades oportunistas agudas o activas 48 semanas antes de comenzar el estudio.
14. Evidencia de hepatocarcinoma o niveles de alfa-fetoproteina ≥ 50 ng/ml, a no ser que una técnica de imagen muestre que no hay evidencia de tumor hepático, todo ello obtenido 24 semanas antes de comenzar el estudio.
15. Hemoglobinopatía u otra enfermedad que pueda facilitar la hemólisis.
16. Transplante de órgano sólido o médula ósea.
17. Hipersensibilidad conocida a alguno de los fármacos objeto del estudio.
18. Consumo activo de drogas o alcohol que en opinión del investigador pudiera interferir con la participación en el estudio. El uso de metadona u otra terapia sustitutiva de los opiáceos no se considerará un criterio de exclusión.
19. Efectos adversos graves con el tratamiento con Peg-IFN más RBV previo en los sujetos con fracaso a dicho tratamiento.

### ***Criterios de retirada:***

1. Imposibilidad para seguir las visitas programadas en el ensayo.
2. Constatación de cumplimentación inferior al 70% de las dosis previstas del tratamiento del ensayo.
3. Decisión del investigador o del paciente.

## Descripción del tratamiento y exposición

Todos los pacientes incluidos en este ensayo clínico recibirán nitazoxanida 500 mg cada 12 horas durante 4 semanas, seguido por nitazoxanida 500 mg cada 12 horas más interferón pegilado alfa-2b 1.5 μg/Kg/semana más ribavirina ajustada según peso durante 48 semanas. Se aplicarán las reglas de interrupción del tratamiento frente al VHC estándar. Se seguirá a los pacientes hasta que alcancen 72 semanas de seguimiento desde que iniciaron la medicación del ensayo.

## Fuente de información y ámbito

La fuente de información será en todos los casos la historia clínica y el propio paciente. La información será recogida de forma prospectiva en los hospitales participantes mediante la inclusión de los datos en el CRD del estudio.

El ámbito del estudio es hospitalario, y participarán los Servicios de Enfermedades Infecciosas y Medicina Interna de 9 hospitales de las CC.AA de Andalucía y Madrid.

## Variables del estudio

###

### ***Variable principal***

La consecución de RVS, definida como la proporción de pacientes que presenta ARN del VHC ≤10 UI/ml 24 semanas después de haber terminado el tratamiento programado.

### ***Variables secundarias***

1. La frecuencia de sujetos con ARN del VHC ≤10 UI/ml 12 semanas después de haber terminado el tratamiento programado.
2. La proporción de pacientes con ARN del VHC ≤10 UI/ml en las semanas 4 y 12 después del inicio de PegIFN más RBV.
3. La frecuencia de efectos adversos graves, grado 3-4 según la clasificación de la OMS.

### ***Variables explicativas o covariables***

Se incluirán en este estudio las siguientes variables potencialmente asociadas con la respuesta al tratamiento frente al VHC: Género, edad, vía de adquisición de la infección por VHC, índice de masa corporal, recuento de células CD4, carga viral VIH, TAR, presencia de sida previo, resistencia insulínica determinada por HOMA, colesterol LDL, carga viral del VHC, fibrosis hepática, genotipo de la interleuquina 28B (IL28B) y adherencia al tratamiento. En los sujetos NR se considerará, además, si presentaron una respuesta nula, es decir la caída de ARN del VHC en la semana 12 fue <2 log_10_, o si habían recidivado después de conseguir una respuesta al final del primer tratamiento. Para el análisis de efectos adversos se recogerá la presencia de síntomas generales, enfermedades psiquiátricas, alteraciones cutáneas, trastornos visuales, toxicidad cardíaca, descompensaciones de la cirrosis, signos de toxicidad mitocondrial, enfermedades autoinmunes, trastornos tiroideos, síntomas digestivos, peso, niveles de hemoglobina, leucocitos, plaquetas, ALT, AST, GGT, fosfatasa alcalina, bilirrubina y creatinina.

## Justificación del tamaño muestral

En el caso de los individuos sin tratamiento con interferón previo, se podría alcanzar una tasa de RVS aproximadamente un 30% mayor de lo observado con tratamiento estándar, suponiendo un comportamiento similar al de los monoinfectados (referencia 16). Se incluirían 49 pacientes sin tratamiento previo con los que se alcanzaría una precisión del 10%, con un intervalo de confianza asintótico normal al 95% bilateral, asumiendo que la proporción de sujetos con RVS es del 50%, y que dispondríamos a alrededor de 100 candidatos en los centros participantes. Al alcanzar la mitad del tamaño muestral estimado para los pacientes no pretratados se llevará a cabo un análisis intermedio. La tasa de recidivas se estima que será del 5%, de manera que, al menos, un 55% (intervalo de confianza 95%: 40%-69%) pacientes deberían alcanzar la respuesta al final de tratamiento. Si en al análisis intermedio no se observa ningún incremento de la RVS con el tratamiento con nitazoxanida más PegIFN y RBV sobre los datos de la cohorte HEPAVIR, se podría proponer interrumpir el ensayo.

En el caso de los sujetos sin RVS previa, se podría estimar una tasa de RVS del 25% a partir de los datos en monoinfectados (referencia 18). Se incluirían 15 pacientes sin RVS previa con los que se conseguiría una precisión del 20%, con un intervalo de confianza asintótico normal al 95% bilateral, asumiendo que la proporción de individuos con RVS será del 20%. El cálculo de los tamaños muestrales se ha realizado por medio del paquete informático Ene 3.0 (e-Biometria, Madrid, España).

## Métodos para la obtención de datos

## Manejo de los datos

Con el fin de garantizar la confidencialidad de los datos del estudio, sólo tendrán acceso a los mismos, el investigador y su equipo de colaboradores, el promotor o la persona que éste designe, el CEIC, las autoridades sanitarias pertinentes y los responsables del análisis de los mismos.

El contenido de los cuadernos de recogida de datos, así como los documentos generados durante el estudio y la base de datos, serán protegidos de usos no permitidos por personas ajenas a la investigación y, por tanto, serán considerados estrictamente confidenciales y no serán revelados a terceros.

El tratamiento de los datos de carácter personal requeridos en este estudio se rige por la Ley Orgánica 15/1999 de 13 de diciembre de Protección de Datos de Carácter Personal.

## Análisis de los datos

Una vez finalizado el estudio, después del registro de los datos de la última visita del último paciente incluido en el mismo, se procederá al cierre de la base de datos, y ésta será transferida al promotor, responsable del análisis estadístico.

La propuesta de métodos de análisis estadístico que se muestra a continuación, constituye una síntesis de los métodos a emplear sobre los datos recogidos, para dar respuesta a los objetivos del estudio.

Se analizarán los datos de la población de pacientes que cumplan los criterios de selección del estudio.

Se hará una descriptiva general de las variables incluidas en el estudio. Se presentarán las distribuciones de frecuencias absolutas y relativas de las variables cualitativas, así como las medidas de tendencia central y dispersión (media desviación típica, mediana, mínimo y máximo) de las variables cuantitativas. Se presentarán los intervalos de confianza al 95% para las principales variables cuantitativas de resultados asociadas al objetivo principal y las principales variables secundarias.

No se imputarán los datos en ausencia y se dejarán como perdidos. De resultar de interés algún subgrupo en particular de pacientes, podrán realizarse los sub-análisis para estos grupos en correspondencia con los análisis planteados para la población general.

### ***Aspectos específicos del análisis estadístico***

Se compararán las tasas de RVS obtenidas por los sujetos sin tratamiento previo incluidos en el ensayo con aquellas observadas en sujetos con genotipo 4 incluidos en la cohorte HEPAVIR de la SAEI, una cohorte de pacientes coinfectados cuyo primer tratamiento frente al VHC es Peg-IFN más RBV. Para esta comparación se considerarán solamente los pacientes incluidos en la cohorte HEPAVIR de la SAEI entre enero de 2007 y enero de 2010. Las tasas de RVS de los sujetos sin respuesta a un tratamiento con Peg-IFN más RBV incluidos en el ensayo se compararán con las de los pacientes sin respuesta a esa combinación incluidos en estudios previos (Crespo M, et al. J Antimicrob Chemother 2008; 62:793-796; Labarga et al. J Acquir Immune Defic Syndr 2010; 53: 364–368). La comparación de las tasas de RVS se realizará por medio del test de la Chi-cuadrado. Las tasas de RVS de los sujetos incluidos en el ensayo se determinarán por protocolo y por intención de tratar. Se llevará a cabo un análisis descriptivo para detectar posibles asociaciones con la RVS, tanto en los sujetos no tratados anteriormente como en los NR. Si se considera oportuno, se examinarán las asociaciones independientes por medio de modelos de regresión logística. El análisis estadístico se realizará por medio de los paquetes SPSS 15 (SPSS Inc., Chicago, IL, EE.UU.) y STATA SE 9 (StataCorp, College Station, TX, EE.UU.).

# MEDICACIÓN DEL ESTUDIO

## Fármacos en investigación

El fármaco en investigación, nitazoxanida 500 mg, será entregado a los centros participantes en el estudio en envases de 60 comprimidos. El etiquetado será individual para cada paciente solicitado. La etiqueta será facilitada por Distefar del Sur, con las características solicitadas por el promotor.

La RBV y el Peg-IFN alfa-2b serán adquiridos localmente (envases comerciales). La RBV se entregará en frascos de 168 comprimidos de 200 mg. Peg-IFN alfa-2b se entregará plumas precargadas que contendrán 50, 80, 100, 120 ó 150 µg de Peg-IFN alfa-2b en 0.5 ml para inyección s.c.

## Dispensación de los fármacos del estudio

El encargado del Servicio de Farmacia del centro participante identificará el fármaco del estudio que debe dispensarse a cada paciente. Inmediatamente antes de dispensar los fármacos del estudio al paciente, el personal de investigación documentará el número específico del paciente en la parte despegable de la etiqueta del estudio. Luego, se retirará la parte despegable y se pegará en el CRD.

El investigador explicará cuidadosamente al paciente las instrucciones para la correcta administración de nitazoxanida, el Peg-IFN alfa-2b y la RBV. Se le explicará también que deberá cumplimentar un diario en casa para registrar las dosis diarias (nitazoxanida y RBV) y semanales (Peg-IFN alfa-2b) que se administra. Las instrucciones para el almacenamiento de los fármacos del estudio figurarán en el etiquetado. Hasta su dispensación, los fármacos quedarán almacenados en el centro participante, siempre con el empaquetado original y en un lugar seguro.

## Recepción, almacenamiento y control de los fármacos del estudio

Los fármacos del estudio serán recepcionados en el centro participante por la persona designada para ello. En todo momento, los paquetes serán manipulados y almacenados de forma adecuada y segura. El almacenamiento se realizará en un lugar seguro al cual sólo puedan acceder el investigador y los colaboradores por él designados. A la recepción, todos los fármacos del estudio deben ser almacenados según las instrucciones que figuran en el etiquetado. Los suministros de cualquier material clínico se harán sólo en función de lo indicado en el protocolo.

El farmacéutico del centro dispensará los fármaco en investigación para cada paciente en función de la evolución del tratamiento. Al farmacéutico del centro participante se le entregará un manual de instrucciones con información detallada sobre cómo dispensar la medicación. A los pacientes se les entregará suficiente cantidad del fármaco en investigación y del material necesario para la autoadministración con el fin de que puedan administrarse todas las dosis programadas entre una visita y otra. Además del suministro de medicación, se entregará a cada paciente un manual de instrucciones con información detallada. El etiquetado de la medicación estará en castellano y cumplirá con la normativa actual vigente. En el etiquetado se incluirán las instrucciones de almacenamiento. Se pedirá al paciente que devuelva en cada visita la medicación que no haya utilizado, así como los recipientes y envases de la medicación que haya utilizado. En cada nueva visita se dispensará al paciente la medicación y el material que necesite.

El investigador debe mantener un registro preciso de la medicación recibida en el centro participante y dispensada a los pacientes, utilizando para ello un libro de contabilidad de la medicación. El control y seguimiento de la contabilidad de la medicación serán realizados por el monitor de campo durante las visitas que realice al centro y al final de estudio. Se pedirá al paciente que devuelva la medicación y materiales, usados o no, al terminar el estudio o en caso de retirada prematura.

Al terminar el estudio y, si es el caso, durante el desarrollo del mismo, el investigador devolverá al promotor toda la medicación no utilizada, así como los recipientes y envases de la medicación que se haya utilizado, los paquetes, las etiquetas y una copia del libro de contabilidad de la medicación.

## Trazabilidad:

La trazabilidad sanitaria del medicamento, suministrado para el ensayo, será garantizada con el albarán de entrega donde aparecerá la descripción del producto, número de unidades, lote y fecha de caducidad que se emitirá por triplicado con destino a la Farmacia Hospitalaria. Dos de los ejemplares serán sellados a la entrega por la farmacia, para la conformidad de la entrega y posterior custodia por el Director Técnico de Distefar Sur, durante un periodo de quince años como establece la ley del medicamento.

### *Trazabilidad medicación dispensada*

Para garantizar la trazabilidad de los fármacos del estudio existirá el anexo “Registro de dispensación de pacientes” (Anexo 2) de obligado cumplimiento en cada dispensación y que incluirá los apartados (identificación del paciente, identificación del medicamento, cantidades dispensadas, nº lote dispensado en cada dispensación y fecha caducidad, fecha devolución y cantidad sobrante). De esta manera se conocerá expresamente a qué paciente se le ha dispensado un determinado lote y en qué fecha se realizó esta dispensación.

Este registro, será complementario al que habitualmente se realice en cada Servicio de Farmacia para muestras de ensayos clínicos de acuerdo al R.D 223/2004.

Se pedirá al paciente que devuelva en cada visita la medicación que no haya utilizado, así como los recipientes y envases de la medicación que haya utilizado. En cada nueva visita se dispensará al paciente la medicación y el material que necesite.

## Ajuste de la dosis y suspensión del tratamiento

### Nitazoxanida

No se realizarán ajustes individuales de la dosis del fármaco en investigación, de acuerdo con los datos recogidos en la ficha técnica actualmente disponible del producto.

### Tratamiento de referencia

En los pacientes tratados con Peg-IFN alfa-2b se observa con frecuencia fiebre, depresión y disminución de ciertos parámetros hematológicos (principalmente leucocitos y plaquetas). Es habitual en la práctica clínica ajustar la dosis de Peg-IFN alfa-2b y de RBV en función de los resultados del hemograma completo (HC) y del recuento de plaquetas. Puede verse un resumen de las directrices para la modificación de la dosis del tratamiento de referencia en el la tabla 1. Si un paciente presenta un recuento de plaquetas <50.000/mm^3^, hemoglobina < 8,5 g/dl o un recuento de neutrófilos < 750 mm^3^, el investigador debe notificarlo al promotor. El promotor, a su vez, dará las orientaciones oportunas sobre los ajustes de dosis en caso de otra toxicidad de grado 3 o 4 y sobre la posible suspensión del tratamiento en un paciente concreto.

**Resumen de las directrices para la modificación de la dosis del tratamiento de referencia:**

A continuación, se resumen las recomendaciones que aparecen en el prospecto que figura en el envase del Peg-IFN alfa-2b:

El tratamiento con Peg-IFN alfa-2b está asociado con una disminución de los recuentos de leucocitos, neutrofilos, linfocitos y plaquetas, que generalmente aparece durante las 2 primeras semanas de tratamiento. Cuando es necesario realizar una modificación de la dosis por reacciones adversas de moderadas a graves (clínicas y/o analíticas), la reducción de la dosis inicial se hará a 1 µg/Kg/semana. Si es necesaria una segunda reducción ésta se hará a 0.5 µg/Kg/semana. Una vez que han mejorado las reacciones adversas, se puede volver a aumentar la dosis. Las directrices para la modificación de la dosis del Peg-IFN alfa-2b serán las que se recogen en la ficha técnica del fármaco y en Documento de consenso de la sobre el manejo de la infección por VHC en pacientes infectados por VIH grupo HEPAVIR de la sociedad andaluza de enfermedades infecciosas (SAEI) (Tabla 1). La toxicidad principal asociada con la RBV es la anemia hemolítica. Si es necesario reducir las dosis de RBV, las disminuciones se harán de 200 mg en 200 mg diarios. Las directrices sobre el ajuste de dosis de RBV proceden de su ficha técnica y del Documento de consenso de la sobre el manejo de la infección por VHC en pacientes infectados por VIH grupo HEPAVIR de la SAEI (Tabla 1).

**Tabla 1.** Directrices para el manejo de la toxicidad hematológica asociada al tratamiento con Peg-IFN alfa-2b más RBV.

| **Alteración hematológica** | **Nivel** | **Recomendación** |
| --- | --- | --- |
| Neutropenia |  |  |
|  | >500 cel/ml | Seguimiento analítico |
|  | <500 cel/ml | Iniciar G-CSF 300 mg 2 veces/semana  Reducción de la dosis de Peg-IFN si G-CSF no eficaz |
| Anemia |  |  |
|  | >10 g/dl | EPO 40000 UI/semana y/o trasfusión de concentrados de hematíes si:   - Descenso >4 g/dl en primeras 4 semanas ó - Descompensación de enf. crónica previa |
|  | 8-10 g/dl | 1. EPO 40.000 UI/semana. 2. EPO 60000 UI/semana si tras 2 semanas con EPO no aumento de Hb ≥1 g/dl. 3. Reducción de RBV en 200 mg/día (valoración en 1 semana) si con EPO no Hb ≥10 g/dl. |
|  | <8 g/dl | Según de síntomas:   1. Reducir la dosis de RBV gradualmente. 2. Suspensión de RBV temporal o definitiva. 3. Transfusión de concentrados de hematíes. |
| Trombopenia |  |  |
|  | <30000/ml | Reducir dosis Peg-IFN* |
|  | <20000/ml | Suspender Peg-IFN* |

*Se considerará el nivel de plaquetas al inicio del tratamiento, el ritmo de descenso y las manifestaciones clínicas.

**Medicación de rescate**

No se permite el uso de medicación de rescate durante el estudio.

**Otros tratamientos concomitantes**

La administración de medicamentos concomitantes no permitidos puede hacer que el paciente tenga que ser excluido del estudio. Las decisiones sobre la exclusión de pacientes que requieren un tratamiento concomitante se tomarán con la participación del promotor del estudio y siempre en función e las circunstancias que concurran en cada caso.

No se permiten los medicamentos específicos contra el VHC dentro de los 3 meses anteriores a la administración de la primera dosis de los fármacos del estudio.

Se deberá llegar a un acuerdo entre investigador y promotor, antes de la inclusión del paciente en el estudio, sobre el uso de medicamentos no incluidos en la tabla 2, siempre y cuando quepa esperar que no van influir en los resultados del estudio.

Todas los medicamentos, tanto prescritos como comprados sin receta médica, incluyendo los complementos vitamínicos, que el paciente haya tomado dentro de los 28 días anteriores al comienzo del estudio o que tome durante el mismo, deberán registrarse en la página de medicamentos / tratamientos no farmacológicos concomitantes del CRD. Se debe preguntar a los pacientes específicamente por el uso de productos de fitoterapia y complementos vitamínicos. Se permite tomar medicamentos para la fiebre o para el dolor muscular (p. ej.,paracematol) durante el tratamiento con Peg-IFN, pero la dosis no debe exceder de 3 gr/día y debe documentarse.

### Tratamientos concomitantes no permitidos

Con respecto a los medicamentos que, según la información que figura en ficha técnica, están contraindicados cuando se administran junto a la ciclosporina A o interfieren con la absorción, distribución, metabolismo o excreción de la ciclosporina A, el investigador deberá consultar con el promotor del estudio. Se requiere la autorización del promotor antes de que el paciente sea admitido en el estudio.

El investigador explicará a los pacientes que deben notificar al centro participante cualquier nuevo medicamento que empiecen a tomar una vez comenzado el estudio. Todos los medicamentos y tratamientos no farmacológicos relevantes (incluyendo fisioterapia y transfusiones de sangre) que se inicien después de la administración de la primera dosis de los fármacos del estudio deben registrarse en el apartado de medicamentos / tratamiento no farmacológico concomitante del CRD.

Los medicamentos deben consignarse en el CRD por su nombre comercial, dosis, frecuencia y vía de administración. También se registrarán las fechas de inicio y finalización del tratamiento y la razón por la que el paciente lo tomó.

**Tabla 2.** Tratamientos concomitantes no permitidos

| **Medicamento** | **Duración** |
| --- | --- |
| Antivirales  (no incluye tratamiento frente al VIH) | Desde 3 meses antes de la primera dosis de la medicación del estudio y hasta la visita final |
| Inmunosupresores | Desde 3 meses antes de la primera dosis de la medicación del estudio y hasta la visita final |
| Fitoterapia  (pej Hierba de San Juan...) | Desde 3 meses antes de la primera dosis de la medicación del estudio y hasta la visita final |

**-Suspensión del tratamiento con los fármacos del estudio y abandono del estudio antes de su finalización**

Por suspensión del tratamiento con los fármacos del estudio se entiende la interrupción de carácter permanente de nitazoxanida e Peg-IFN alfa-2b y RBV. El tratamiento con los fármacos del estudio debe suspenderse en un paciente concreto cuando el investigador determine que continuar con el tratamiento conllevaría un riesgo significativo para la seguridad del paciente.

En el caso de que se produzca un AAG, el promotor valorará la seguridad e, inmediatamente, orientará al investigador sobre la suspensión del tratamiento y la retirada prematura del estudio. Las siguientes circunstancias pueden requerir la suspensión del tratamiento:

- Solicitud de suspensión realizada por el promotor después de revisar los datos de seguridad en un grupo de tratamiento.
- Embarazo
- Aparición de toxicidad inaceptable a pesar de haberse reducido la dosis y/o suspendido el tratamiento provisionalmente.
- Utilización de los siguientes tratamientos/medicamentos prohibidos:
  - Tratamiento crónico con corticoesteroides sistémicos (equivalente de prednisona de > 10 mg/día durante más de 2 semanas).
  - Otros fármacos en investigación o ya comercializados para tratar la infección producida por el VHC (p. ej., Peg-IFN alfa-2a, IFN/RBV)
  - Cualquier otro fármaco en investigación distinto al del estudio.
- Retirada del consentimiento informado.
- Cumplimiento inferior al 70% de las dosis del tratamiento en cualquiera de las visitas de seguimiento.
- Cualquier otra violación del protocolo que dé lugar a un riesgo importante para la seguridad del paciente.

Además de estas normas y requerimientos para la suspensión del tratamiento, el investigador puede suspender el tratamiento del estudio en un paciente concreto, si, después de considerar los “pros” y los “contras”, llega a la conclusión de que continuar con el tratamiento sería perjudicial para el bienestar del paciente.

Se deberá cumplimentar el apartado de terminación de la fase de tratamiento del CRD, haciendo constar la fecha y la razón principal por la que se suspende el tratamiento.

Los pacientes en los que se suspende el tratamiento NO deben ser considerados pacientes que se retiran del estudio antes de su terminación. Si un paciente se retira del estudio, debe realizarse la evaluación final del estudio. En el caso de los pacientes que se pierden para el seguimiento (es decir, aquellos cuya situación no está clara porque no acuden a las visitas del estudio, pero tampoco han comunicado al personal investigador su deseo de retirarse del estudio), el investigador debe hacer todo lo que esté en su mano para contactar con el paciente, registrando en los documentos fuente las gestiones que ha realizado (p. ej., fechas en las que llamó al paciente por teléfono, cartas certificadas que ha enviado a su domicilio, etc).

Los pacientes en los que se suspenda el tratamiento del estudio no serán sustituidos por otros reclutados para reemplazarlos.

### Terminación del estudio y tratamiento post-estudio

El periodo de inclusión de pacientes en el estudio terminará cuando se haya completado el tamaño muestral del estudio o si el estudio se interrumpe antes de la fecha prevista. Si un paciente se encuentra en el periodo de selección una vez que el estudio ha terminado, seguirá siendo elegible para participar en el mismo.

Para cada paciente, el estudio se considerará terminado cuando se haya realizado la visita de seguimiento para la evaluación de la seguridad de la semana 72.

Si el paciente se hubiese retirado prematuramente del estudio, tendrá que volver al centro participante para la visita final del estudio. El investigador debe proporcionar atención médica de seguimiento adecuada a todos los pacientes que se hayan retirado prematuramente del estudio o bien debe derivarlos a otros profesionales para continuar con una atención médica adecuada.

**Terminación del estudio antes de la fecha prevista**

El estudio puede quedar suspendido hasta que sea posible realizar una evaluación más detallada si el promotor así lo solicita sobre la base de los criterios de seguridad preestablecidos. Además, el estudio puede darse por concluido en cualquier momento y por cualquier razón si así lo decide el promotor y así lo comunique y obtenga el visto bueno de los CEIC correspondiente. Si fuese necesario tomar esta decisión, los pacientes deben ser visitados lo antes posible y tratados de acuerdo con lo dispuesto en el Apartado nº de Aptdo de retirada prematura de un paciente. El investigador puede recibir información sobre otros procedimientos a realizar con el fin de garantizar que los pacientes reciben la debida consideración para proteger sus intereses. El investigador será responsable de informar al CRI y/o al CIE sobre la terminación del estudio antes de la fecha prevista.

#

# SEGURIDAD

## Exploración física

Se realizará una exploración física completa en las Visitas 0, 12,24,48,72 semanas. La exploración incluirá el examen del aspecto general del paciente, piel, cuello (incluyendo la tiroides), ojos, oídos, nariz, garganta, pulmones, corazón, abdomen, espalda, ganglios linfáticos, extremidades y vasos sanguíneos, así como una exploración neurológica. Si está indicado en función de los datos de la anamnesis/historia clínica y/o síntomas, se hara también una exploración del recto, genitales externos, mamas y pelvis.

La exploración física abreviada incluirá el examen del aspecto general del paciente y las constantes vitales (temperatura corporal, presión sanguínea y frecuencia respiratoria y del pulso). Se realizará una exploración física abreviada en todas las visitas en las que no está prevista una exploración física completa.

La información procedente de las exploraciones físicas debe incluirse en la documentación fuente. Los hallazgos relevantes que estén presentes antes de la administración de la primera dosis de los fármacos del estudio se harán constar en el apartado de antecedentes médicos/ enfermedades actuales relevantes del CRD del paciente. Los hallazgos relevantes que se observen después de la primera dosis de los fármacos del estudio que satisfagan los criterios de AA se registrarán en el apartado de acontecimientos adversos del CRD del paciente.

## Constantes vitales

Las constantes vitales se evaluarán en todas las visitas. Incluirá la temperatura corporal, la presión arterial y la frecuencia respiratoria y del pulso.

Una vez que el paciente haya permanecido sentado durante cinco minutos con la espalda apoyada en el respaldo de la silla y los pies en el suelo, se medirá tres veces la PS sistólica y diastólica mediante un dispositivo validado que disponga de un manguito de tamaño adecuado. Las medidas con el paciente sentado se obtendrán a intervalos de 1-2 minutos, y se anotará la media de las tres mediciones. En caso de que el manguito del dispositivo del que se dispone no sea suficiente para abarcar el perímetro del brazo del paciente, se utilizará un esfigmomanómetro con un manguito de tamaño adecuado.

## Estatura y peso corporal

La estatura se medirá en centímetros (cm) en la semana 0. El peso corporal (redondeando al 0,1 kilogramo [Kg] más próximo) se medirá con ropa normal, pero descalzo. La medición de peso se hará en todas las visitas.

## Evaluaciones de laboratorio

Se utilizará los laboratorios de cada centro para el análisis de todas las muestras recogidas durante el estudio. En el Anexo 1 (CRD) aparecen las pruebas de laboratorio que se harán durante el estudio y el momento de la recogida de las muestras.

## Pruebas de laboratorio

**- Hematología analítica**

Como se muestra en la Tabla 3 y el Anexo 1 las pruebas de hematología analítica se realizarán en todas las visitas.

**-Análisis bioquímico de la sangre**

Como se muestra en la Tabla 3 y el Anexo 1, el análisis bioquímico de la sangre se realizará en todas las visitas.

**-Análisis de orina**

Como se muestra en la Tabla 3 y el Anexo 1, los análisis de orina se harán en todas las visitas.

**-Electrocardiograma (ECG)**

Se realizará un ECG convencional de 12 derivaciones en las semanas 0, 12, 24, 48 y 72 o cuando un paciente se retire del estudio antes de su finalización. La interpretación del trazado electrocardiográfico debe hacerse por un médico cualificado y se documentará en el apartado del ECG del CRD. Todos los trazados ECG llevarán el número del estudio, las iniciales del paciente, el número del paciente y la fecha, y se guardarán en el centro participante con los documentos fuente. Se documentarán únicamente las anomalías que sean clínicamente relevantes. Las anomalías clínicamente significativas se registrarán también en el apartado de antecedentes médicos/historia clínica/enfermedades actuales relevantes del CRD. Los hallazgos clínicamente significativos observados en el ECG se notificarán al promotor antes de incluir al paciente en el estudio.

## Embarazo y evaluación de la fertilidad

Todas las mujeres, con independencia del estatus con respecto a la fertilidad autoinformado, se someterán a la prueba del embarazo a partir de un muestra de sangre durante el periodo de selección (visita 1) y a partir de un muestra de orina una vez al mes desde la Semana 2 hasta la Semana 72.

Un resultado positivo en la prueba del embarazo a partir de una muestra de orina requiere la suspensión inmediate del tratamiento con los fármacos del estudio hasta que se determine el nivel sérico de GCh-B y el resultado sea negativo. Si es positivo, la paciente debe retirarse del estudio.

En todas las especies animales estudiadas, se ha demostrado que la RBV tiene efectos teratogénicos y embrionicidas importantes. Por lo tanto, no se debe administrar RBV a mujeres embarazadas o que piensan quedarse embarazadas en un periodo de menos de 6 meses después de la finalización del tratamiento con RBV. Tampoco se debe administrar RBV a los hombres cuya compañera está embarazada o piensa quedarse embarazada durante el estudio o un periodo de menos de 7 meses después del tratamiento con RBV.

Todas las pacientes en edad reproductiva deben someterse a la prueba del embarazo una vez al mes mientras estén siendo tratadas y durante un periodo de 6 meses después de la última dosis de RBV. En cada una de las visitas programadas al centro participante que tenga lugar después de la visita de selección, se hará la prueba del embarazo en la orina.

Los casos de embarazo deben notificarse al promotor tan pronto como el personal del centro participante tenga conocimiento del hecho. Para una información más detallada sobre los procedimientos para notificar los embarazos, véase el Apartado (Embarazos). Se notificarán y se hará un seguimiento de todos los embarazos que se produzcan desde la administración de los fármacos del estudio el Día 1 hasta la Semana 48 en el caso de las pacientes que han completado las 24 semanas de tratamiento o 24 semanas después de la última dosis de RBV en el caso de las pacientes que han recibido menos de 24 semanas de tratamiento.

## Otras pruebas para evaluar la seguridad

**-Agudeza visual**

El examen de la agudeza visual se realizará por los médicos responsables de los pacientes. Si se observan cambios en la agudeza visual, el paciente se someterá a una exploración oftalmológica completa.

**-Escala de depresión**

La depresión se cribará mediante la escala CES-D abreviada que aparece en el Apéndice 7 en las semanas 0, 12, 24, 48. Si se detecta un caso de depresión se evaluará mediante la escala de depresión de Hamilton (Apéndice 7).

**-Monitorización de los acontecimientos adversos oftalmológicos**

Durante la evaluación rutinaria de los acontecimientos adversos el investigador o personal cualificado para ello preguntará al paciente sobre los acontecimientos adversos oftalmológicos (oculares). Estas preguntas se incluirán en la evaluación general de los acontecimientos adversos, en la que se preguntará por otros órganos o sistemas. Se hace así para que el autoinforme del paciente sobre acontecimientos adversos oculares no esté sesgado.

**-Validez de las medidas utilizadas para evaluar la seguridad**

Las evaluaciones de seguridad elegidas para este estudio son las convencionales en esta indicación y población de pacientes. Algunas medidas de seguridad adicionales se han incluido con el fin de monitorizar los acontecimientos observados en los estudios preclínicos de seguridad.

**-Visitas no programadas para evaluar la seguridad**

Se permitirán visitas no programadas para evaluar la seguridad en cualquier momento a lo largo del estudio, siempre que el investigador lo considere clínicamente justificado. Si es necesario hacer una visita no programada, el personal del estudio recogerá las constantes vitales, los acontecimientos adversos y la medicación concomitante (si es el caso) y llevará a cabo aquellas exploraciones y evaluaciones que se consideren necesarias.

# ACONTECIMIENTOS ADVERSOS

Un acontecimiento adverso (AA) consiste en la aparición o el empeoramiento de cualquier signo, síntoma o condición médica no deseados después de empezar el tratamiento con el fármaco en investigación, incluso cuando se considera que el acontecimiento no está relacionado con el fármaco en investigación. Por fármaco en estudio se entiende el fármaco que se está evaluando y el comparador que se administran durante cualquiera de las fases del estudio. Las condiciones médicas/enfermedades que estuvieran presentes antes de empezar el tratamiento con el fármaco en investigación se consideran AA únicamente si empeoran después de empezar el tratamiento con el fármaco en investigación. Los valores anormales de las pruebas de laboratorio o los resultados positivos de cualquier otra prueba se consideran AA únicamente cuando provocan signos o síntomas clínicos, se consideran clínicamente relevantes o requieren tratamiento.

La presencia de los AA debe buscarse haciendo al paciente en cada visita preguntas que no le sugieran o le empujen a dar una respuesta afirmativa. Los AA también pueden detectarse cuando el paciente habla de ellos de forma espontánea durante o fuera de las visitas, mediante la exploración física, las pruebas de laboratorio u otras evaluaciones.

Todos los AA que se detecten entre el comienzo del tratamiento con el fármaco en investigación el Día 1 y el final del estudio (es decir, 24 semanas después de la última dosis de los fármacos del estudio o cuando un paciente se retire del estudio antes de su finalización) se registrarán en el apartado de acontecimientos adversos del CRD.

Se realizará un seguimiento de los pacientes que presenten un acontecimiento adverso grave (AAG, véase definición en el apartado 12.2) que no se haya resuelto o estabilizado antes de la Semana 24. Se contactará con el paciente al menos cada 12 semanas hasta que el AAG se haya resuelto o estabilizado. Si no es posible averiguar cuál ha sido el desenlace del AAG (p. ej., el paciente se ha perdido para el seguimiento, ha retirado el consentimiento), el investigador debe documentar la razón por la que no se ha podido averiguar el desenlace del AAG.

Todos los AA se anotarán en el apartado correspondiente del CRD adjuntando la siguiente información:

## Intensidad

Los AA se valorarán utilizando las tablas modificadas de toxicidad de la Division of Microbiology and Infectious Diseases (DMID). Se aplican los siguientes criterios de severidad para los AA no incluidos en esas tablas de toxicidad:

•Leve: no provoca ninguna limitación en las actividades habituales del paciente

•Moderado: provoca algunas limitaciones en las actividades habituales del paciente

•Intenso: provoca incapacidad para realizar las actividades habituales del paciente, puede poner en peligro la vida del paciente* - puede causar la muerte o discapacidad permanente.

*Nota: El grado de intensidad “puede poner en peligro la vida del paciente” no es necesariamente el mismo que “potencialmente mortal”. Lo segundo significa que el AA representa un riesgo inminente para la vida del paciente, mientras que lo primera quiere decir sólo que podría poner en peligro la vida del paciente.

## Relación con el o los fármacos en investigación

• Existe sospecha

• No existe sospecha

**Duración**

Fecha de aparición y de resolución o si continúa estando presente en la evaluación final.

**Gravedad**

Anotar si se trata de un AAG.

Un AAG es un AA que:

• es mortal o puede poner en peligro la vida del paciente

• provoca una discapacidad importante o persistente

• constituye un anomalía o defecto congénito

• requiere la hospitalización del paciente o la prolongación de la hospitalización si el paciente se encontraba ya hospitalizado, a no ser que:

• la hospitalización sea para controlar o llevar a cabo un procedimiento rutinario de la indicación del estudio no relacionado con un deterioro de la enfermedad.

• la hospitalización se deba a un tratamiento previamente programado para una enfermedad o trastorno preexistente que no tiene ninguna relación con la indicación del estudio y que no ha empeorado desde el inicio del tratamiento con el fármaco en investigación.

• tratamiento de urgencia como paciente ambulatorio por algún acontecimiento que no reúne ninguno de los criterios de la definición de AAG citados más arriba y no requiere hospitalización.

• la hospitalización se debe a razones sociales o se hace para que el paciente o su cuidador habitual descansen, sin que se haya producido deterioro alguno en el estado general del paciente.

• el AA es médicamente relevante (p. ej., pone en peligro al paciente o puede requerir una intervención médica o quirúrgica para prevenir alguna de las situaciones citadas más arriba).

Al contrario de los que sucede con las evaluaciones rutinarias de la seguridad, los AAG deben ser monitorizados continuamente y siguen procedimientos especiales para su notificación (ver apartado 12.4).

Todos los AA deben ser tratados adecuadamente. Las acciones emprendidas para tratar los AA se anotarán en el apartado de acontecimientos adversos del CRD.

El manual del investigador (MI) contiene información sobre los efectos secundarios ya conocidos del fármaco en investigación. Si entre una actualización y otra del MI, el promotor del estudio tuviese conocimiento de efectos secundarios del fármaco en investigación hasta ese momento no conocidos, lo comunicaría a los investigadores a través de las Notificaciones para el Investigador. Esto debe incluirse en la hoja informativa para solicitar el consentimiento informado y, siempre que sea necesario, se le explicará al paciente durante el estudio.

## Reacción adversa

Una reacción adversa (RA) es toda reacción nociva y no intencionada a un medicamento en investigación, independientemente de la dosis administrada. En una RA existe una sospecha de relación causal entre el medicamento en investigación y el acontecimiento adverso. La relación causal será sospechada por el investigador correspondiente fundamentándola en el perfil de reacciones adversas conocidos e incluidos en las fichas técnicas de nitazoxanida, Peg-IFN alfa-2b y RBV. Reacción adversa grave (RAG) es aquella que es mortal o puede poner en peligro la vida del paciente, provoca una discapacidad importante o persistente, constituye un anomalía o defecto congénito, requiere la hospitalización del paciente o la prolongación de la hospitalización si el paciente se encontraba ya hospitalizado. Se considerarán RAG aquellos que sean médicamente relevantes, aunque no cumplan los criterios anteriores de RAG (p. ej., pone en peligro al paciente o puede requerir una intervención médica o quirúrgica para prevenir alguna de las situaciones citadas más arriba). También se notificarán como graves todas las sospechas de transmisión de un agente infeccioso a través de un medicamento.

Una reacción adversa inesperada (RAI) es cualquier reacción cuya naturaleza o consecuencias no se corresponden con la información de referencia para el medicamento. El carácter inesperado de una RA se basa en el hecho de no haber sido previamente observada y no ser anticipable por las propiedades farmacológicas del medicamento.

## Notificación de los acontecimientos adversos graves

Para garantizar la seguridad de los pacientes, todos los AAG, con independencia de si existe o no sospecha de una relación causal con el fármaco en investigación, y todos los RAG o RAGI que aparezcan una vez el paciente ha empezado a tomar el fármaco en investigación y hasta 30 días después de que haya terminado la participación del paciente en el estudio, se notificarán a el promotor.

Los AAG, RAG o RAGI que aparezcan después de este periodo de 30 días se notificarán a el promotor sólo si el investigador sospecha que tiene relación causal con el fármaco en investigación. Las recurrencias, las complicaciones y el progreso del AAG, RAG o RAGI inicial deben notificarse como parte del seguimiento que se hace del episodio inicial, con independencia de en qué momento se producen. La notificación debe hacerse en el plazo de 24 horas a contar desde el momento en el que el investigador recibe la información de seguimiento. Si se considera que un AAG, RAG o RAGI no está en absoluto relacionado con otro AAG, RAG o RAGI que ya se ha notificado, el investigador debe notificarlo como un AAG, RAG o RAGI nuevo.

La información sobre los AAG, RAG y RAGI se recogerá y registrará en el formulario de acontecimientos adversos graves. El investigador debe evaluar la posible relación entre el AAG o RAG y el fármaco en investigación, cumplimentar el formulario de acontecimientos adversos graves, firmarlo y enviarlo por fax al teléfono 955015461. En la carpeta del investigador que se entrega a todos los centros participantes figura el número de teléfono y de telecopia de la persona encargada de la Seguridad Clínica y Epidemiología del promotor. El original del formulario para la notificación de AAG o RAG y la hoja de confirmación del envío por fax deben guardarse como parte del CRD en el centro participante.

La información del seguimiento del AAG o RAG se enviará a la misma persona que el formulario para la notificación de AAG o RAG, utilizando para ello un nuevo formulario en el que se hará constar que se trata del seguimiento de un AAG o RAG ya notificado y la fecha del formulario del AAG o RAG original. En la información de seguimiento se especificará si el AAG o RAG se ha resuelto o continúa, si ha sido o no tratado, de qué forma se ha tratado y si el paciente continúa participando en el estudio o se ha retirado.

Si el AAG o RAG no está documentado en el MI o en el prospecto del producto (AAG nuevo o RAGI) y se considera que está relacionado con el fármaco en investigación, el promotor puede pedir más información urgentemente sobre el AAG o RAG con el fin de informar a las autoridades sanitarias. El promotor enviará a todos los investigadores implicados en el estudio una Notificación para el Investigador, informando de que se ha notificado un AAG nuevo o un RAGI. Las RAGI se registrarán y notificarán por el promotor a las autoridades sanitarias competentes y a los comités de ética en cumplimiento del RD 233/2003 del 6 de Febrero y/o de las leyes y normas regulatorias actuales, específicamente:

- **Notificación de las RAGI asociadas a nitazoxanida**

La notificación se realizará por fax (918225076). Las sospechas de reacciones adversas se acompañarán de la carta de acompañamiento indicada en el Anexo 3.

- **Notificación de RAGI asociadas a interferón pegilado alfa 2-b y ribavirina**

La notificación se realizará por fax (918225336). Las sospechas de reacciones adversas se acompañarán de la carta de acompañamiento indicada en el Anexo 4.

- **Notificación expeditiva de las RAGI a los CEIC y CCAA**

Se notificará a cada uno de los CEIC implicados en un ensayo clínico todas las RAGI que hubieran ocurrido en los sujetos participantes en los centros de su área de influencia. Asimismo, se notificará al órgano competente de cada una de las Comunidades Autónomas donde se realiza el ensayo las sospechas de RAGI ocurridas en los centros sanitarios de su Comunidad. En ambos casos, se utilizará para ello el formulario de notificación que consta como Anexo 5.

El promotor realizará la notificación en formato electrónico de todas las RAGI ocurridas también a Eudravigilance-CT.

## Embarazos

Para garantizar la seguridad de los pacientes, a todos los participantes en edad reproductiva (tanto mujeres como hombres) se les explicará que deben evitar los embarazos y que para ello deben utilizar dos métodos anticonceptivos desde el inicio del estudio hasta 6 meses después de la finalización del tratamiento o de la última dosis de RBV (fechas más tardía de ambas). Todos los embarazos que se produzcan después de que el paciente haya empezado a tomar los fármacos del estudio y hasta seis meses después de la última dosis de RBV (dependiendo de cuál de estas dos fechas es más tardía**)** deben notificarse al promotor en el plazo de 24 horas a contar desde el momento en el que se conoce el embarazo. Debe hacerse un seguimiento de la gestación para determinar el desenlace de la misma, incluyendo el aborto espontáneo, la interrupción voluntaria del embarazo, los detalles del parto y la presencia o ausencia de cualquier defecto o anomalía congénita o de complicaciones en la madre o en el recién nacido. Los embarazos que se produzcan después de firmar el consentimiento informado y antes de que el paciente entre en el estudio se considerarán un criterio de exclusión.

La información del seguimiento del embarazo se registrará en el CRD y se incluirá una evaluación de la posible relación entre el fármaco en investigación y el desenlace de la gestación. Los AAG o RAG que aparezcan durante el embarazo se registrarán en el formulario de AAG o RAG.

También debe registrarse el desenlace de la gestación de la pareja de los pacientes de sexo masculino que participen en el estudio. En estos casos, la madre debe dar su consentimiento para la obtención de dicha información.

## Comité para la Vigilancia de Datos y Seguridad

Se constituirá un Comité de Vigilancia de Datos y Seguridad (CVDS). Estará formado por los investigadores principales y por el promotor.

El CVDS se regirá por una serie de normas que se redactarán antes de la primera visita del primer paciente. En estas normas se incluirán las recomendaciones para el ajuste de la dosis de los fármacos del estudio (si es necesaria), los criterios para retirar un paciente, los criterios para suspender un grupo de tratamiento y los criterios para suspender el estudio.

Es necesario que el CVDS evalúe inmediatamente los AAG o RAG durante los primeros 3 meses del tratamiento, con el fin de proporcionar las recomendaciones oportunas sobre la modificación/suspensión, etc. del tratamiento. Durante el periodo de tratamiento que incluye el tratamiento de referencia, los pacientes pueden ser tratados de acuerdo con la directrices establecidas en el protocolo y, una vez terminado el tratamiento, los investigadores tomarán las decisiones finales que consideren más convenientes para sus pacientes.

En las normas de funcionamiento del CVDS se especificará la periodicidad y los mecanismos de la comunicación entre los investigadores, el CVDS y el promotor.

# ASPECTOS ÉTICOS / PROTECCIÓN DE LOS SUJETOS PARTICIPANTES

Este estudio clínico se ha diseñado, se realizará y se publicará con pleno cumplimiento de lo dispuesto en las Directrices sobre Buena Práctica Clínica, así como de los dispuesto en las leyes y normas administrativas de España (incluyendo la Directiva Europea 2001/20/EC). Asimismo, respetará los principios éticos establecidos en la Declaración de Helsinki.

## Evaluación beneficio-riesgo

Los pacientes incluidos en este ensayo recibirán el tratamiento estándar actual frente al VHC, Peg-IFN más RBV, del que se conocen su seguridad y eficacia en sujetos infectados por VIH/VHC. Además, se añadirá a este tratamiento estándar NTZ, un fármaco empleado ampliamente como antiparasitario, incluyendo experiencia de uso en sujetos infectados por VIH. La NTZ ha sido segura en el contexto de tratamiento de parasitosis. Por otro lado, en pacientes infectados únicamente por VHC, el uso de NTZ más Peg-IFN y RBV no incrementó significativamente los efectos adversos asociados a la combinación de Peg-IFN más RBV. Finalmente, la adición de NTZ al tratamiento estándar frente al VHC supuso un aumento significativo en la tasa de RVS de los pacientes infectados por VHC-4. Por todo ello, la relación riesgo-beneficio de una combinación de NTZ más Peg-IFN más RBV en pacientes coinfectados por VIH/VHC-4 será favorable.

## Hoja de información y formulario de consentimiento

A cada sujeto que se le proponga participar en el estudio, se le entregará un documento escrito denominado “Hoja de información al paciente”, en la que, de forma detallada, se referirán el objetivo y la descripción del estudio, procedimientos del estudio, duración prevista y número de sujetos participantes, beneficios y riesgos posibles derivados de su participación en el mismo, confidencialidad de los datos de carácter personal, así como datos de contacto del médico responsable del estudio, tal y como se presenta en el Anexo 7 de este protocolo. Este documento estará redactado con un vocabulario que permita que su contenido sea completamente legible y comprensible para el paciente.

El investigador deberá informar al paciente sobre la naturaleza voluntaria de su participación y que no supone ningún cambio ni en su tratamiento ni en su atención médica respecto a los que recibiría de no participar. Él contestará a sus dudas y preguntas y de acuerdo con la normativa vigente obtendrá el consentimiento del sujeto o en su defecto, de un testigo imparcial, en cuyo caso firmará el consentimiento informado oral ante testigos (Anexo 8).

El sujeto participante en el estudio podrá revocar en cualquier momento su consentimiento para la utilización de sus datos en el análisis, sin expresión de causa y sin que por ello se derive para él responsabilidad ni perjuicio alguno.

## Confidencialidad de los datos

La recogida de los datos se realizará mediante la cumplimentación de una base de datos electrónica realizada específicamente para este estudio. Los datos de seguimiento de los pacientes deberán incluirse en la base hasta la finalización del periodo de estudio. Los coordinadores del proyecto podrán acceder a la base de datos completa, aunque no podrán modificar los datos ya introducidos. Únicamente el responsable final del fichero y su análisis podrá corregir errores de introducción o valoración, previa consulta con el facultativo responsable de cada caso.

Se adoptarán las medidas técnicas y organizativas necesarias para garantizar la seguridad de los datos de carácter personal y evitar su alteración, pérdida, tratamiento o acceso no autorizado. No se introducirán datos que permitan la identificación del paciente, ya que se utilizará un sistema de codificación cuya correlación real sólo será conocida por el investigador responsable de cada centro.

El tratamiento de los datos de carácter personal requeridos en este estudio se rige por la Ley Orgánica 15/1999 de 13 de diciembre de Protección de Datos de Carácter Personal. Con el fin de garantizar la confidencialidad de los datos del estudio, sólo tendrán acceso a los mismos, el investigador y su equipo de colaboradores, el promotor o la persona que este designe, el Comité Ético de Investigación Clínica, las autoridades sanitarias pertinentes y los responsables del análisis de los mismos.

El contenido de la base de datos electrónica donde se registre la información, estará codificada y protegida de usos no permitidos por personas ajenas a la investigación y, por tanto, será considerada estrictamente confidencial y no será revelada a terceros excepto a los especificados en el párrafo anterior.

## Responsabilidades del investigador y del CEIC

Antes del inicio del estudio, el protocolo y el modelo de hoja informativa para el paciente/formulario de consentimiento informado deben ser revisados y aprobados por un CEIC constituido según normativa.. Antes de comenzar el estudio, los centros participantes enviarán al promotor un documento firmado y fechado en el que se demuestre que el protocolo y la hoja informativa para el paciente/formulario para el consentimiento informado han sido aprobados por el CEIC. Así mismo, antes del comienzo del estudio, se le pedirá al investigador que firme en la página de firmas del protocolo para confirmar que acepta realizar el estudio de acuerdo con las instrucciones y procedimientos contenidos en el protocolo y que se compromete a permitir el acceso a todos los documentos y registros relevantes a los monitores del estudio y a los representantes del CEIC Y de las autoridades reguladoras en caso de requerirlo. Si los organismos reguladores oficiales solicitan realizar una inspección del centro participante en el estudio, el investigador deberá ponerlo en conocimiento del promotor de inmediato.

# PLANES DE DIFUSIÓN DE LOS RESULTADOS

Toda la información relacionada con el estudio es considerada confidencial y propiedad del promotor hasta su publicación, no podrá ser revelada a otros sin previo consentimiento escrito del promotor y no podrá ser utilizada excepto para la realización de este estudio.

Sólo el promotor o sus representantes pueden hacer extensible a los médicos y a los organismos reglamentarios la información obtenida en este estudio, excepto si es requerido mediante una orden.

Los resultados del estudio serán publicados en revistas científicas, y/o difundidos a través de comunicaciones a congresos.

Respecto a la política de autorías y publicación de resultados, se observarán las normas establecidas por el promotor:

- La publicación del estudio se realizará en revistas científicas y con mención de los Comités Éticos de Investigación Clínica correspondientes.
- El orden de autores en las publicaciones será el siguiente: como primer autor aparecerá el investigador coordinador del estudio, y como co-autores el resto de investigadores por orden de aparición según el número de pacientes reclutados, y teniendo en cuenta la limitación del número de autores establecido por la editorial de la revista a la que se envíe el manuscrito para su publicación.
- Cuando se haga público el desarrollo y resultados del estudio, en todo caso, se hará constar el origen de los fondos para su realización.
- Se mantendrá en todo momento el anonimato de los casos incluidos en el estudio.
- Los resultados o conclusiones de este estudio se comunicarán, de forma prioritaria, en publicaciones científicas antes de ser divulgados al público no sanitario.

# FINANCIACIÓN

El promotor, Dr. Juan Macías Sánchez, garantiza la no interferencia en los procesos de selección de los casos, análisis de la información y/o presentación de resultados, o cualquier otro proceso que pueda incidir en los resultados del estudio.

La financiación del estudio de acuerdo con las directrices del presente protocolo procederá de la convocatoria 2010 de Ayudas para el Fomento de la Investigación Clínica Independiente del Ministerio de Sanidad, Política Social e Igualdad (expediente EC10-187). La financiación será en todo caso independiente de los resultados del estudio.

En todo caso el promotor del estudio dispondrá de una memoria económica del estudio que estará disponible para su consulta en el momento en que sea necesario.

# MODIFICACIONES DEL PROTOCOLO

Todas las modificaciones que sufra el presente protocolo serán comunicadas al CEIC que haya realizado su evaluación. En el caso de enmiendas relevantes, aquellas que afectan a aspectos fundamentales del protocolo del estudio como objetivos, métodos o aspectos éticos, se someterá de nuevo a la evaluación del CEIC que informó favorablemente sobre el mismo, y se solicitará autorización administrativa para dicha enmienda. Para el resto de enmiendas bastará con la notificación al CEIC, justificando el motivo por el que no se considera relevante.

## Adherencia al protocolo

Los investigadores deben hacer todo lo posible para evitar desviaciones del protocolo. Bajo ninguna circunstancia el investigador se pondrá en contacto con el promotor para solicitar autorización para realizar una desviación del protocolo, ya que no se permite ninguna violación del mismo. Si el investigador considera que una violación del protocolo mejoraría el desarrollo del estudio, debe tener en cuenta que lo que está proponiendo es una enmienda al protocolo, y no podrá poner en práctica tal enmienda si antes no es aprobada por el promotor y los correspondientes CEIC. Todas las violaciones relevantes del protocolo serán registradas y comunicadas en el CRD.

# CONSIDERACIONES PRÁCTICAS

## Informes de seguimiento y final

El cierre definitivo del ensayo se realizará una vez se hayan completado todos los datos del último paciente incluido en el estudio. Tras el cierre de la base de datos se realizará el análisis estadístico y se presentará un informe con los datos descriptivos, que será revisado y aprobado por el promotor del estudio.

El promotor redactará dos informes de seguimiento semestrales y un informe final de acuerdo a la legislación vigente. Asimismo, el promotor informará de forma inmediata sobre cualquier incidencia relevante (interrupción problema grave de seguridad, etc.) que pueda producirse en el transcurso del estudio. Todas estas comunicaciones se presentarán a los órganos competentes de las CC.AA. involucradas y a la AEMPS.

Se remitirá un informe final entre los 3 y 6 meses después de la finalización del estudio a la AEMPS y a los órganos competentes de las CC.AA. donde se realizó.

## Difusión de los resultados

Se realizará tal como se ha descrito en el apartado 14.

## Responsabilidades del promotor

Las responsabilidades del promotor del estudio son:

- Firmar con el investigador coordinador el protocolo y cualquier modificación del mismo.
- Suministrar a los investigadores el protocolo y la ficha técnica de los medicamentos del estudio si aplica
- Remitir el protocolo al CEIC.
- Solicitar la autorización de la Administración, cuando proceda, y presentar la documentación correspondiente.
- Presentar los informes de seguimiento y final, en los plazos establecidos y comunicar, en su caso, la interrupción y las razones de la misma.
- Entregar copia del protocolo y de los documentos que acrediten el seguimiento de los procedimientos establecidos en las presentes directrices a los responsables de las entidades proveedoras de servicios de atención a la salud donde se vaya a realizar el estudio.
- Aplicar un control de calidad en la obtención y el manejo de datos para asegurar que los datos son fiables.
- Identificar las fuentes de financiación del estudio.
- Firmar, en su caso, el contrato con la entidad competente.
- Hacer públicos los resultados del estudio, a ser posible, a través de una revista científica.

## Responsabilidades del investigador

Las responsabilidades del investigador en cada centro participante serán:

- Firmar un compromiso en el que se reconocen como investigadores del estudio y afirman que conocen el protocolo y cualquier modificación del mismo, y están de acuerdo con él en todos sus términos.
- Informar a los sujetos de investigación y obtener su consentimiento.
- Recoger, registrar y notificar los datos de forma correcta respondiendo de su actualización y calidad ante las auditorias oportunas.
- Notificar al promotor los acontecimientos adversos según se establezca en el protocolo.
- Respetar la confidencialidad de los datos de los sujetos participantes en el estudio.
- Facilitar las auditorias del promotor y las inspecciones de las autoridades sanitarias.
- Saber responder sobre los objetivos, metodología básica y significado de los resultados del estudio ante la comunidad científica y profesional.

## Responsabilidades del investigador coordinador

Las responsabilidades del investigador coordinador del estudio son las enumeradas anteriormente para el investigador más las siguientes:

- Firmar el protocolo y cualquier modificación del mismo junto con el promotor.
- Co-responsabilizarse con el promotor de la elaboración de los informes de seguimiento y finales.
- Contribuir a difundir los resultados del estudio, en colaboración con el promotor.

# Anexo 1. CUADERNO DE RECOGIDA DE DATOS

**Criterios de inclusión:**

1. Infección por VIH.
2. Infección por el genotipo 4 del VHC.
3. No tratamiento previo con ningún tipo de interferón o ausencia de respuesta a un primer tratamiento previo con Peg-IFN más RBV. La falta de respuesta incluirá tanto a los sujetos no respondedores, como a los que mostraron recidivas.
4. TAR estable 24 semanas antes de comenzar el estudio, con ARN plasmático del VIH indetectable durante ese periodo de tiempo.
5. Compromiso de usar dos métodos anticonceptivos no hormonales durante el estudio y hasta 24 semanas después del mismo.
6. Aceptación de dar el consentimiento informado por escrito para participar en el ensayo.

**Criterios de exclusión:**

1. Inclusión de didanosina, estavudina, zidovudina o abacavir en el TAR.
2. Cirrosis descompensada.
3. Presencia de otras enfermedades hepáticas significativas, incluidas hepatitis crónica o aguda por virus de la hepatitis B, hepatitis aguda por virus de la hepatitis A, hemocromatosis o déficit de alfa-1 antitripsina.
4. Embarazo o lactancia.
5. Hombres con planes de embarazo con sus parejas durante el estudio y hasta 24 semanas después de terminado el tratamiento.
6. Depresión activa o mal controlada, otras enfermedades psiquiátricas, o cualquier enfermedad a lo largo del año previo que pueda, en opinión del investigador, impedir la participación en el estudio.
7. Intento de suicido previo.
8. Enfermedad tiroidea activa o mal controlada con tratamiento.
9. Enfermedades autoinmunes previas como enfermedad inflamatoria intestinal, psosiasis grave, o artritis reumatoide, que pudieran exacerbarse con el interferón.
10. Tratamiento quimioterápico o inmunomodulatorio 24 semanas antes de comenzar el estudio.
11. Enfermedad grave, incluyendo cáncer o enfermedad coronaria no estable, 24 semanas antes de comenzar el estudio.
12. Cualquier enfermedad o problema crónico que, en opinión del investigador, puede impedir completar el estudio.
13. Presencia de enfermedades oportunistas agudas o activas 48 semanas antes de comenzar el estudio.
14. Evidencia de hepatocarcinoma o niveles de alfa-fetoproteina ≥ 50 ng/ml, a no ser que una técnica de imagen muestre que no hay evidencia de tumor hepático, todo ello obtenido 24 semanas antes de comenzar el estudio.
15. Hemoglobinopatía u otra enfermedad que pueda facilitar la hemólisis.
16. Transplante de órgano sólido o médula ósea.
17. Hipersensibilidad conocida a alguno de los fármacos objeto del estudio.
18. Consumo activo de drogas o alcohol que en opinión del investigador pudiera interferir con la participación en el estudio. El uso de metadona u otra terapia sustitutiva de los opiáceos no se considerará un criterio de exclusión.
19. Efectos adversos graves con el tratamiento con Peg-IFN más RBV previo en los sujetos con fracaso a dicho tratamiento.

**Esquema de visitas:**

| **Evaluación** | **Semanas (Visitas)** | | | | | | | | | | | | | |
| --- | --- | --- | --- | --- | --- | --- | --- | --- | --- | --- | --- | --- | --- | --- |
|  | **Cribado**  **(V1)** | **0 (V2)** | **4 (V3)** | **8 (V4)** | **12 (V5)** | **16 (V6)** | **20 (V7)** | **24 (V8)** | **28 (V9)** | **36 (V10)** | **44 (V11)** | **52 (V12)** | **64 (V13)** | **76 (V14)** |
| Procedimientos del estudio |  |  |  |  |  |  |  |  |  |  |  |  |  |  |
| Consentimiento informado |  | x |  |  |  |  |  |  |  |  |  |  |  |  |
| Historia médica y examen físico | x | x | x |  | x |  |  | x |  | x |  | x |  | × |
| Signos vitales | x | x | x | x | x | x | x | x | x | x | × | x | × | × |
| Intervención |  |  |  |  |  |  |  |  |  |  |  |  |  |  |
| NTZ monoterapia |  | x | x |  |  |  |  |  |  |  |  |  |  |  |
| NTZ+PegIFN+RBV |  |  | x | x | x | x | x | x | x | x | x | x |  |  |
| Evaluaciones analíticas |  |  |  |  |  |  |  |  |  |  |  |  |  |  |
| Test de embarazo (orina o suero) | x | x | x | x | x | x | x | x | x | x | x | x | x | x |
| ARN de VIH plasmático | x | x |  |  | x |  |  | x |  | x |  | x |  | x |
| Recuento de células CD4+ y CD8+ | x | x |  |  | x |  |  | x |  | x |  | x |  | × |
| Hematología | x | x | x | x | x | x | x | x | x | x | × | × | × | × |
| Bioquímica | x | x | x | x | x | x | x | x | x | x | × | × | × | × |
| Perfil lipídico |  | x | x |  | x |  |  | x |  | x |  | x |  | x |
| Análisis de orina | x | x | x | x | x | x | x | x | x | x | × | × | × | × |
| Test de función tiroidea | x | x | x | x | x | x | x | x | x | x | x | x | x | × |
| HOMA |  | x |  |  |  |  |  |  |  |  |  | x |  | x |
| Polimorfismos de IL28B |  | x |  |  |  |  |  |  |  |  |  |  |  |  |
| Pruebas de imagen y otras |  |  |  |  |  |  |  |  |  |  |  |  |  |  |
| Fibrosis hepática por medio de elastometría transitoria |  | x |  |  |  |  |  |  |  |  |  |  |  | x |
| ECG |  | x |  |  | x |  |  | x |  |  |  | x |  | x |
| Medidas de eficacia |  |  |  |  |  |  |  |  |  |  |  |  |  |  |
| ARN de VHC plasmático | x | x | x | x | x | x | x | x | x | x | x | x | x | × |
| Evaluación cumplimentación tratamiento |  |  | x | x | x | x | x | x | x | x | × | × |  |  |
| Medidas de seguridad |  |  |  |  |  |  |  |  |  |  |  |  |  |  |
| Efectos adversos |  |  | x | x | x | x | x | x | x | x | × | × |  |  |
| Medicación concomitante | x | x | x | x | x | x | x | x | x | x | × | × | × | × |

**VISITA 1 DE CRIBADO**

**FECHA (DD/MM/YY):___/___/___**

| **Historia médica y examen físico** | Recoger en historia del paciente |  |
| --- | --- | --- |
| **Signos vitales** |  |  |
|  | Estatura (cm) |  |
|  | Peso (Kg) |  |
|  | Temperatura (ºC) |  |
|  | Presión arterial (mmHg) |  |
|  | Frecuencia cardíaca (spm) |  |
| **Test de embarazo (orina o suero)** |  |  |
| **ARN de VHC plasmático** |  |  |
| **ARN de VIH plasmático** |  |  |
| **Hematología** |  |  |
|  | Leucocitos (K/μL) |  |
|  | Neutrófilos (K/μL) |  |
|  | Linfocitos (K/μL) |  |
|  | Recuento de CD4+ (cel/μL) |  |
|  | Recuento de CD8+ (cel/μL) |  |
|  | Hemoglobina (g/dL) |  |
|  | VCM (fL) |  |
|  | Plaquetas (K/μL) |  |
|  | INR |  |
|  | Actividad de protrombina (%) |  |
| **Bioquímica** |  |  |
|  | ALT (U/L) |  |
|  | AST (U/L) |  |
|  | GGT (U/L) |  |
|  | Fosfatasa alcalina (U/L) |  |
|  | Colinesterasa (U/L) |  |
|  | Bilirrubina total (mg/dL) |  |
|  | Bilirrubina directa (mg/dL) |  |
|  | Urea (mg/dL) |  |
|  | Creatinina (mg/dL) |  |
|  | Glucosa (mg/dL) |  |
|  | Albúmina (mg/dL) |  |
| **Perfil lipídico** |  |  |
|  | Colesterol total (mg/dL) |  |
|  | Colesterol LDL (mg/dL) |  |
|  | Colesterol HDL (mg/dL) |  |
|  | Triglicéridos (mg/dL) |  |
| **Análisis de orina** |  |  |
|  | Sedimento urinario |  |
|  | Proteinuria (mg/dL) |  |
|  | Hematíes (cel/μL) |  |
|  | Leucocitos (cel/μL) |  |
| **Test de función tiroidea** |  |  |
|  | TSH (μU/mL) |  |
|  | T4L (si procede) (ng/dL) |  |
|  | T3L (si procede) (ng/dL) |  |
| **Medicación concomitante** |  |  |

**VISITA 2, SEMANA 0**

**FECHA (DD/MM/YY):___/___/___**

| **Historia médica y examen físico** | Recoger en historia del paciente |  |
| --- | --- | --- |
| **Signos vitales** |  |  |
|  | Estatura (cm) |  |
|  | Peso (Kg) |  |
|  | Temperatura (ºC) |  |
|  | Presión arterial (mmHg) |  |
|  | Frecuencia cardíaca (spm) |  |
| **Test de embarazo (orina o suero)** |  |  |
| **ARN de VHC plasmático** |  |  |
| **ARN de VIH plasmático** |  |  |
| **Hematología** |  |  |
|  | Leucocitos (K/μL) |  |
|  | Neutrófilos (K/μL) |  |
|  | Linfocitos (K/μL) |  |
|  | Recuento de CD4+ (cel/μL) |  |
|  | Recuento de CD8+ (cel/μL) |  |
|  | Hemoglobina (g/dL) |  |
|  | VCM (fL) |  |
|  | Plaquetas (K/μL) |  |
|  | INR |  |
|  | Actividad de protrombina (%) |  |
| **Bioquímica** |  |  |
|  | ALT (U/L) |  |
|  | AST (U/L) |  |
|  | GGT (U/L) |  |
|  | Fosfatasa alcalina (U/L) |  |
|  | Colinesterasa (U/L) |  |
|  | Bilirrubina total (mg/dL) |  |
|  | Bilirrubina directa (mg/dL) |  |
|  | Urea (mg/dL) |  |
|  | Creatinina (mg/dL) |  |
|  | Glucosa (mg/dL) |  |
|  | Albúmina (mg/dL) |  |
|  | Insulina (mU/mL) |  |
|  | HOMA |  |
| **Perfil lipídico** |  |  |
|  | Colesterol total (mg/dL) |  |
|  | Colesterol LDL (mg/dL) |  |
|  | Colesterol HDL (mg/dL) |  |
|  | Triglicéridos (mg/dL) |  |
| **Análisis de orina** |  |  |
|  | Sedimento urinario |  |
|  | Proteinuria (mg/dL) |  |
|  | Hematíes (cel/μL) |  |
|  | Leucocitos (cel/μL) |  |
| **Test de función tiroidea** |  |  |
|  | TSH (μU/mL) |  |
|  | T4L (si procede) (ng/dL) |  |
|  | T3L (si procede) (ng/dL) |  |
| **Polimorfismo IL28B (**rs12979860) | CC/CT/TT |  |
| **Elastometría transitoria hepática** | (KPa) |  |
| **Medicación concomitante** |  |  |
| **ECG** |  |  |

**VISITA 3, SEMANA 4.**

**FECHA (DD/MM/YY):___/___/___**

| **Historia médica y examen físico** | Recoger en historia del paciente |  |
| --- | --- | --- |
| **Signos vitales** |  |  |
|  | Peso (Kg) |  |
|  | Temperatura (ºC) |  |
|  | Presión arterial (mmHg) |  |
|  | Frecuencia cardíaca (spm) |  |
| **Test de embarazo (orina o suero)** |  |  |
| **ARN de VHC plasmático** |  |  |
| **Hematología** |  |  |
|  | Leucocitos (K/μL) |  |
|  | Neutrófilos (K/μL) |  |
|  | Linfocitos (K/μL) |  |
|  | Hemoglobina (g/dL) |  |
|  | VCM (fL) |  |
|  | Plaquetas (K/μL) |  |
|  | INR |  |
|  | Actividad de protrombina (%) |  |
| **Bioquímica** |  |  |
|  | ALT (U/L) |  |
|  | AST (U/L) |  |
|  | GGT (U/L) |  |
|  | Fosfatasa alcalina (U/L) |  |
|  | Colinesterasa (U/L) |  |
|  | Bilirrubina total (mg/dL) |  |
|  | Bilirrubina directa (mg/dL) |  |
|  | Urea (mg/dL) |  |
|  | Creatinina (mg/dL) |  |
|  | Glucosa (mg/dL) |  |
|  | Albúmina (mg/dL) |  |
| **Perfil lipídico** |  |  |
|  | Colesterol total (mg/dL) |  |
|  | Colesterol LDL (mg/dL) |  |
|  | Colesterol HDL (mg/dL) |  |
|  | Triglicéridos (mg/dL) |  |
| **Análisis de orina** |  |  |
|  | Sedimento urinario |  |
|  | Proteinuria (mg/dL) |  |
|  | Hematíes (cel/μL) |  |
|  | Leucocitos (cel/μL) |  |
| **Test de función tiroidea** |  |  |
|  | TSH (μU/mL) |  |
|  | T4L (si procede) (ng/dL) |  |
|  | T3L (si procede) (ng/dL) |  |
| **Cumplimentación del tratamiento** |  |  |
|  | Peg-IFN, dosis (%cumplimentación) |  |
|  | RBV, dosis (%cumplimentación) |  |
|  | NTZ, dosis (%cumplimentación) |  |
| **Medicación concomitante** |  |  |
| **Efectos adversos** |  |  |

**VISITAS DE SEMANAS 12 (V5), 24 (V8) y 36 (10).**

**FECHA (DD/MM/YY):___/___/___**

| **Historia médica y examen físico** | Recoger en historia del paciente |  |
| --- | --- | --- |
| **Signos vitales** |  |  |
|  | Peso (Kg) |  |
|  | Temperatura (ºC) |  |
|  | Presión arterial (mmHg) |  |
|  | Frecuencia cardíaca (spm) |  |
| **Test de embarazo (orina o suero)** |  |  |
| **ARN de VHC plasmático** |  |  |
| **ARN de VIH plasmático** |  |  |
| **Hematología** |  |  |
|  | Leucocitos (K/μL) |  |
|  | Neutrófilos (K/μL) |  |
|  | Linfocitos (K/μL) |  |
|  | Recuento de CD4+ (cel/μL) |  |
|  | Recuento de CD8+ (cel/μL) |  |
|  | Hemoglobina (g/dL) |  |
|  | VCM (fL) |  |
|  | Plaquetas (K/μL) |  |
|  | INR |  |
|  | Actividad de protrombina (%) |  |
| **Bioquímica** |  |  |
|  | ALT (U/L) |  |
|  | AST (U/L) |  |
|  | GGT (U/L) |  |
|  | Fosfatasa alcalina (U/L) |  |
|  | Colinesterasa (U/L) |  |
|  | Bilirrubina total (mg/dL) |  |
|  | Bilirrubina directa (mg/dL) |  |
|  | Urea (mg/dL) |  |
|  | Creatinina (mg/dL) |  |
|  | Glucosa (mg/dL) |  |
|  | Albúmina (mg/dL) |  |
| **Perfil lipídico** |  |  |
|  | Colesterol total (mg/dL) |  |
|  | Colesterol LDL (mg/dL) |  |
|  | Colesterol HDL (mg/dL) |  |
|  | Triglicéridos (mg/dL) |  |
| **Análisis de orina** |  |  |
|  | Sedimento urinario |  |
|  | Proteinuria (mg/dL) |  |
|  | Hematíes (cel/μL) |  |
|  | Leucocitos (cel/μL) |  |
| **Test de función tiroidea** |  |  |
|  | TSH (μU/mL) |  |
|  | T4L (si procede) (ng/dL) |  |
|  | T3L (si procede) (ng/dL) |  |
| **Cumplimentación del tratamiento** |  |  |
|  | Peg-IFN, dosis (%cumplimentación) |  |
|  | RBV, dosis (%cumplimentación) |  |
|  | NTZ, dosis (%cumplimentación) |  |
| **Medicación concomitante** |  |  |
| **Efectos adversos** |  |  |
| **ECG** |  |  |

**VISITAS DE SEMANAS 8 (V4), 16 (V6), 20 (V7), 28 (V9) y 44 (V11).**

**FECHA (DD/MM/YY):___/___/___**

| **Historia médica y examen físico** | Recoger en historia del paciente |  |
| --- | --- | --- |
| **Signos vitales** |  |  |
|  | Peso (Kg) |  |
|  | Temperatura (ºC) |  |
|  | Presión arterial (mmHg) |  |
|  | Frecuencia cardíaca (spm) |  |
| **Test de embarazo (orina o suero)** |  |  |
| **ARN de VHC plasmático** |  |  |
| **Hematología** |  |  |
|  | Leucocitos (K/μL) |  |
|  | Neutrófilos (K/μL) |  |
|  | Linfocitos (K/μL) |  |
|  | Hemoglobina (g/dL) |  |
|  | VCM (fL) |  |
|  | Plaquetas (K/μL) |  |
|  | INR |  |
|  | Actividad de protrombina (%) |  |
| **Bioquímica** |  |  |
|  | ALT (U/L) |  |
|  | AST (U/L) |  |
|  | GGT (U/L) |  |
|  | Fosfatasa alcalina (U/L) |  |
|  | Colinesterasa (U/L) |  |
|  | Bilirrubina total (mg/dL) |  |
|  | Bilirrubina directa (mg/dL) |  |
|  | Urea (mg/dL) |  |
|  | Creatinina (mg/dL) |  |
|  | Glucosa (mg/dL) |  |
|  | Albúmina (mg/dL) |  |
| **Análisis de orina** |  |  |
|  | Sedimento urinario |  |
|  | Proteinuria (mg/dL) |  |
|  | Hematíes (cel/μL) |  |
|  | Leucocitos (cel/μL) |  |
| **Test de función tiroidea** |  |  |
|  | TSH (μU/mL) |  |
|  | T4L (si procede) (ng/dL) |  |
|  | T3L (si procede) (ng/dL) |  |
| **Cumplimentación del tratamiento** |  |  |
|  | Peg-IFN, dosis (%cumplimentación) |  |
|  | RBV, dosis (%cumplimentación) |  |
|  | NTZ, dosis (%cumplimentación) |  |
| **Medicación concomitante** |  |  |
| **Efectos adversos** |  |  |

**VISITA 12, SEMANA 52.**

**FECHA (DD/MM/YY):___/___/___**

| **Historia médica y examen físico** | Recoger en historia del paciente |  |
| --- | --- | --- |
| **Signos vitales** |  |  |
|  | Peso (Kg) |  |
|  | Temperatura (ºC) |  |
|  | Presión arterial (mmHg) |  |
|  | Frecuencia cardíaca (spm) |  |
| **Test de embarazo (orina o suero)** |  |  |
| **ARN de VHC plasmático** |  |  |
| **ARN de VIH plasmático** |  |  |
| **Hematología** |  |  |
|  | Leucocitos (K/μL) |  |
|  | Neutrófilos (K/μL) |  |
|  | Linfocitos (K/μL) |  |
|  | Recuento de CD4+ (cel/μL) |  |
|  | Recuento de CD8+ (cel/μL) |  |
|  | Hemoglobina (g/dL) |  |
|  | VCM (fL) |  |
|  | Plaquetas (K/μL) |  |
|  | INR |  |
|  | Actividad de protrombina (%) |  |
| **Bioquímica** |  |  |
|  | ALT (U/L) |  |
|  | AST (U/L) |  |
|  | GGT (U/L) |  |
|  | Fosfatasa alcalina (U/L) |  |
|  | Colinesterasa (U/L) |  |
|  | Bilirrubina total (mg/dL) |  |
|  | Bilirrubina directa (mg/dL) |  |
|  | Urea (mg/dL) |  |
|  | Creatinina (mg/dL) |  |
|  | Glucosa (mg/dL) |  |
|  | Albúmina (mg/dL) |  |
|  | Insulina (mU/mL) |  |
|  | HOMA |  |
| **Perfil lipídico** |  |  |
|  | Colesterol total (mg/dL) |  |
|  | Colesterol LDL (mg/dL) |  |
|  | Colesterol HDL (mg/dL) |  |
|  | Triglicéridos (mg/dL) |  |
| **Análisis de orina** |  |  |
|  | Sedimento urinario |  |
|  | Proteinuria (mg/dL) |  |
|  | Hematíes (cel/μL) |  |
|  | Leucocitos (cel/μL) |  |
| **Test de función tiroidea** |  |  |
|  | TSH (μU/mL) |  |
|  | T4L (si procede) (ng/dL) |  |
|  | T3L (si procede) (ng/dL) |  |
| **Cumplimentación del tratamiento** |  |  |
|  | Peg-IFN, dosis (%cumplimentación) |  |
|  | RBV, dosis (%cumplimentación) |  |
|  | NTZ, dosis (%cumplimentación) |  |
| **Medicación concomitante** |  |  |
| **Efectos adversos** |  |  |
| **ECG** |  |  |

**VISITA 13, SEMANA 64.**

**FECHA (DD/MM/YY):___/___/___**

| **Historia médica y examen físico** | Recoger en historia del paciente |  |
| --- | --- | --- |
| **Signos vitales** |  |  |
|  | Peso (Kg) |  |
|  | Temperatura (ºC) |  |
|  | Presión arterial (mmHg) |  |
|  | Frecuencia cardíaca (spm) |  |
| **Test de embarazo (orina o suero)** |  |  |
| **ARN de VHC plasmático** |  |  |
| **Hematología** |  |  |
|  | Leucocitos (K/μL) |  |
|  | Neutrófilos (K/μL) |  |
|  | Linfocitos (K/μL) |  |
|  | Hemoglobina (g/dL) |  |
|  | VCM (fL) |  |
|  | Plaquetas (K/μL) |  |
|  | INR |  |
|  | Actividad de protrombina (%) |  |
| **Bioquímica** |  |  |
|  | ALT (U/L) |  |
|  | AST (U/L) |  |
|  | GGT (U/L) |  |
|  | Fosfatasa alcalina (U/L) |  |
|  | Colinesterasa (U/L) |  |
|  | Bilirrubina total (mg/dL) |  |
|  | Bilirrubina directa (mg/dL) |  |
|  | Urea (mg/dL) |  |
|  | Creatinina (mg/dL) |  |
|  | Glucosa (mg/dL) |  |
|  | Albúmina (mg/dL) |  |
| **Análisis de orina** |  |  |
|  | Sedimento urinario |  |
|  | Proteinuria (mg/dL) |  |
|  | Hematíes (cel/μL) |  |
|  | Leucocitos (cel/μL) |  |
| **Test de función tiroidea** |  |  |
|  | TSH (μU/mL) |  |
|  | T4L (si procede) (ng/dL) |  |
|  | T3L (si procede) (ng/dL) |  |
| **Medicación concomitante** |  |  |

**VISITA 14, SEMANA 76.**

**FECHA (DD/MM/YY):___/___/___**

| **Historia médica y examen físico** | Recoger en historia del paciente |  |
| --- | --- | --- |
| **Signos vitales** |  |  |
|  | Peso (Kg) |  |
|  | Temperatura (ºC) |  |
|  | Presión arterial (mmHg) |  |
|  | Frecuencia cardíaca (spm) |  |
| **Test de embarazo (orina o suero)** |  |  |
| **ARN de VHC plasmático** |  |  |
| **ARN de VIH plasmático** |  |  |
| **Hematología** |  |  |
|  | Leucocitos (K/μL) |  |
|  | Neutrófilos (K/μL) |  |
|  | Linfocitos (K/μL) |  |
|  | Recuento de CD4+ (cel/μL) |  |
|  | Recuento de CD8+ (cel/μL) |  |
|  | Hemoglobina (g/dL) |  |
|  | VCM (fL) |  |
|  | Plaquetas (K/μL) |  |
|  | INR |  |
|  | Actividad de protrombina (%) |  |
| **Bioquímica** |  |  |
|  | ALT (U/L) |  |
|  | AST (U/L) |  |
|  | GGT (U/L) |  |
|  | Fosfatasa alcalina (U/L) |  |
|  | Colinesterasa (U/L) |  |
|  | Bilirrubina total (mg/dL) |  |
|  | Bilirrubina directa (mg/dL) |  |
|  | Urea (mg/dL) |  |
|  | Creatinina (mg/dL) |  |
|  | Glucosa (mg/dL) |  |
|  | Albúmina (mg/dL) |  |
|  | Insulina (mU/mL) |  |
|  | HOMA |  |
| **Perfil lipídico** |  |  |
|  | Colesterol total (mg/dL) |  |
|  | Colesterol LDL (mg/dL) |  |
|  | Colesterol HDL (mg/dL) |  |
|  | Triglicéridos (mg/dL) |  |
| **Análisis de orina** |  |  |
|  | Sedimento urinario |  |
|  | Proteinuria (mg/dL) |  |
|  | Hematíes (cel/μL) |  |
|  | Leucocitos (cel/μL) |  |
| **Test de función tiroidea** |  |  |
|  | TSH (μU/mL) |  |
|  | T4L (si procede) (ng/dL) |  |
|  | T3L (si procede) (ng/dL) |  |
| **Elastometría transitoria hepática** | (KPa) |  |
| **Medicación concomitante** |  |  |
| **ECG** |  |  |

**Escala del Centro de Estudios Epidemiológicos de la Depresión (Center for Epidemiologic Studies-Depression Scale, CES-D)**


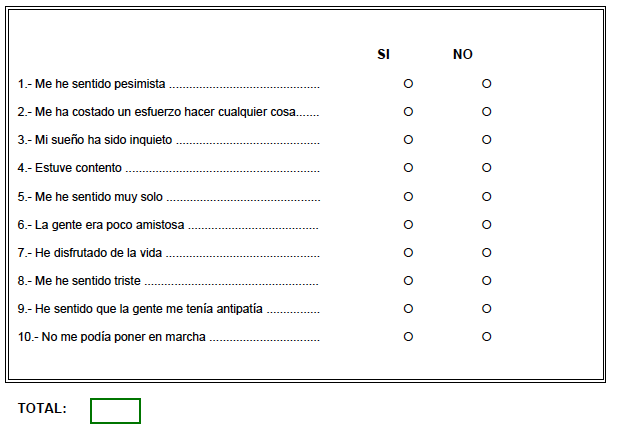


Cada ítem puntúa No=0 ó Sí=1, con un rango de puntuación de 0-10. El punto de corte utilizado es > 4.

**Escala de Hamilton para la Depresión**

Instrucciones para su administración

• Es una escala heteroadministrada por un clínico tras una entrevista.

• La prueba consta de 17 ítems.

• Algunos de los ítems se valoran de 0 a 2 y otros de 0 a 4 puntos.

• El rango de puntuaciones oscila entre 0 y 52 puntos.

• ➢ Una puntuación igual o superior a 25 corresponde a una depresión grave.

• ➢ Una puntuación entre 7 y 17 corresponde a una depresión moderada.

• ➢ Valores inferiores son indicativos de la ausencia o remisión del trastorno.

1. Humor deprimido, tristeza (melancolía), desesperanza, desamparo,

inutilidad:

0 Ausente

1 Estas sensaciones las expresa solamente si le preguntan como se siente

2 Estas sensaciones las relata espontáneamente

3 Sensaciones no comunicadas verbalmente (expresión facial, postura,

voz, tendencia al llanto)

4 Manifiesta estas sensaciones en su comunicación verbal y no verbal

en forma espontánea

2. Sentimiento de culpa:

0 Ausente

1 Se culpa a sí mismo, cree haber decepcionado a la gente

2 Tiene ideas de culpabilidad o medita sobre errores pasados o malas

acciones

3 Siente que la enfermedad actual es un castigo

4 Oye voces acusatorias o de denuncia y/o experimenta alucinaciones

visuales amenazadoras

3. Suicidio:

0 Ausente

1 Le parece que la vida no vale la pena ser vivida

2 Desearía estar muerto o tiene pensamientos sobre la posibilidad de

morirse

3 Ideas de suicidio o amenazas

4 Intentos de suicidio (cualquier intento serio)

4. Insomnio precoz:

0 No tiene dificultad

1 Dificultad ocasional para dormir, por ejemplo le toma más de media

hora el conciliar el sueño

2 Dificultad para dormir cada noche.

5. Insomnio intermedio:

0 No hay dificultad

1 Está desvelado e inquieto o se despierta varias veces durante la noche

2 Está despierto durante la noche, cualquier ocasión de levantarse de la

cama se clasifica en 2 (excepto por motivos de evacuar)

6. Insomnio tardío:

0 No hay dificultad

1 Se despierta a primeras horas de la madrugada, pero se vuelve a dormir

2 No puede volver a dormirse si se levanta de la cama

7. Trabajo y actividades:

0 No hay dificultad

1 Ideas y sentimientos de incapacidad, fatiga o debilidad (trabajos,

pasatiempos)

2 Pérdida de interés en su actividad (disminución de la atención, indecisión

y vacilación)

3 Disminución del tiempo actual dedicado a actividades o disminución

de la productividad

4 Dejó de trabajar por la presente enfermedad. Solo se compromete en

las pequeñas tareas, o no puede realizar estas sin ayuda.

8. Inhibición psicomotora (lentitud de pensamiento y palabra, facultad

de concentración disminuida, disminución de la actividad motora):

0 Palabra y pensamiento normales

1 Ligero retraso en el habla

2 Evidente retraso en el habla

3 Dificultad para expresarse

4 Incapacidad para expresarse

9. Agitación psicomotora:

0 Ninguna

1 Juega con sus dedos

2 Juega con sus manos, cabello, etc.

3 No puede quedarse quieto ni permanecer sentado

4 Retuerce las manos, se muerde las uñas, se tira de los cabellos, se

muerde los labios

10. Ansiedad psíquica:

0 No hay dificultad

1 Tensión subjetiva e irritabilidad

2 Preocupación por pequeñas cosas

3 Actitud aprensiva en la expresión o en el habla

4 Expresa sus temores sin que le pregunten

11. Ansiedad somática (Signos físicos concomitantes de ansiedad tales como:

Gastrointestinales: sequedad de boca, diarrea, eructos, etc. Cardiovasculares:

palpitaciones, cefaleas. Respiratorios: hiperventilación,

suspiros. Frecuencia de micción incrementada. Transpiración):

0 Ausente

1 Ligera

2 Moderada

3 Severa

4 Incapacitante

12. Síntomas somáticos gastrointestinales:

0 Ninguno

1 Pérdida del apetito pero come sin necesidad de que lo estimulen.

Sensación de pesadez en el abdomen

2 Dificultad en comer si no se le insiste. Solicita laxantes o medicación

intestinal para sus síntomas gastrointestinales

13. Síntomas somáticos generales:

0 Ninguno

1 Pesadez en las extremidades, espalda o cabeza. Dorsalgias. Cefaleas,

algias musculares.

2 Pérdida de energía y fatigabilidad. Cualquier síntoma bien definido

se clasifica en 2.

14. Síntomas genitales (tales como: disminución de la libido y trastornos

menstruales):

0 Ausente

1 Débil

2 Grave

15. Hipocondría:

0 Ausente

1 Preocupado de si mismo (corporalmente)

2 Preocupado por su salud

3 Se lamenta constantemente, solicita ayuda

16. Pérdida de peso:

0 Pérdida de peso inferior a 500 g en una semana

1 Pérdida de más de 500 g en una semana

2 Pérdida de más de 1 kg en una semana

17. Perspicacia:

0 Se da cuenta que está deprimido y enfermo

1 Se da cuenta de su enfermedad pero atribuye la causa a la mala alimentación,

clima, exceso de trabajo, virus, necesidad de descanso, etc.

3 No se da cuenta que está enfermo

TOTAL PUNTUACIÓN:

# Anexo 2. REGISTRO DE DISPENSACION DE PACIENTES

| **REGISTRO DISPENSACION PACIENTES ESTUDIO *NTZSPA001*** | | | | | | | |
| --- | --- | --- | --- | --- | --- | --- | --- |
| **FECHA** | **DISPENSACION** | **MEDICAMENTO DISPENSADO** | **CANTIDAD**  **DISPENSADA** | **Nº LOTE** | **FECHA CADUCIDAD** | **FECHA DEVOLUCION** | **CANTIDAD DEVUELTA** |
|  |  |  |  |  |  |  |  |
|  |  |  |  |  |  |  |  |
|  |  |  |  |  |  |  |  |
|  |  |  |  |  |  |  |  |
|  |  |  |  |  |  |  |  |
|  |  |  |  |  |  |  |  |
|  |  |  |  |  |  |  |  |
|  |  |  |  |  |  |  |  |
|  |  |  |  |  |  |  |  |
|  |  |  |  |  |  |  |  |
|  |  |  |  |  |  |  |  |
|  |  |  |  |  |  |  |  |
|  |  |  |  |  |  |  |  |
|  |  |  |  |  |  |  |  |
|  |  |  |  |  |  |  |  |

# Anexo A3. NOTIFICACIÓN DE RAGI A NITAZOXANIDA.

Unidad de Registro y Tasas Agencia Española de Medicamentos y Productos Sanitarios

Destinatario final: Subdirección General de Medicamentos de Uso Humano.

Área de Ensayos Clínicos

Parque empresarial Las Mercedes Edif. 8

C/ Campezo 1, 28022 Madrid (España)

- Fecha : ……………………………………………………………………………

- Promotor - Persona/organización que notifica a la AEMPS: Juan Macías Sánchez

- Nombre del medicamento en investigación al que se asocian las RAGI: Nitazoxanida

**RAGIs notificadas en España (ocurridas en el EC o fuera de él).**

| **RAGI notificadas en España** | | |
| --- | --- | --- |
| **EC referentes al Nº de PEI……..** | Nº RAGI | Comentarios |
| Nº EudraCT |  |  |
|  |  |  |
|  |  |  |
| Nº EudraCT |  |  |
|  |  |  |
|  |  |  |
| Nº EudraCT |  |  |
|  |  |  |
|  |  |  |
| *(…………)* |  |  |
|  |  |  |
|  |  |  |

*Las RAGIs notificadas en España se archivarán en la carpeta 35-00 del EC al que hagan referencia.*

| **RAGIs notificadas fuera de España (dentro o fuera de un EC) que el promotor conoce** | |
| --- | --- |
| Nº RAGI | Comentarios |
|  |  |
|  |  |
|  |  |

*Las RAGIs ocurridas fuera de España (dentro o fuera de un EC) se archivarán en la carpeta 35-01 de todos los EC previamente identificados*

Otra información de interés sobre la notificación:.......................................................................................

..........................................................................................................................................................................

Por la presente confirmo que la información reflejada en esta documentación es veraz

Firma del notificador a la AEMPS

Juan Macías Sánchez

# Anexo 4. NOTIFICACIÓN DE RAGI A INTERFERÓN PEGILADO ALFA 2-B Ó RIBAVIRINA.

Unidad de Registro y Tasas Agencia Española de Medicamentos y Productos Sanitarios

Destinatario final: Subdirección General de Medicamentos de Uso Humano.

Área de Ensayos Clínicos.

Parque empresarial Las Mercedes Edif. 8

C/ Campezo 1, 28022 Madrid (España)

- Fecha : ……………………………………………………………………………
- Promotor - Persona/organización que notifica a la AEMPS: Juan Macías Sánchez
- Nombre del medicamento en investigación al que se refieren las RAGI …………………

RAGI agrupadas por solicitudes nº 16 de EC que se notifican en el mismo CD/DVD

| Nº EudraCT |  | Nº Referencia RAGI | Comentarios |
| --- | --- | --- | --- |
|  | RAGIs del EC en España |  |  |
|  |  |  |  |
|  |  |  |  |
|  | RAGIs del EC fuera de España |  |  |

| Nº EudraCT |  | Nº Referencia RAGI | Comentarios |
| --- | --- | --- | --- |
|  | RAGIs del EC en España |  |  |
|  |  |  |  |
|  |  |  |  |
|  | RAGIs del EC fuera de España |  |  |

** Las RAGIs del EC en España se archivarán en la carpeta 35-00 del EC al que hagan referencia.*

*Las RAGI del EC fuera de España se archivarán en la carpeta 35-01 del EC al que hagan referencia.*

Otra información de interés sobre la notificación: .....................................................................................

........................................................................................................................................................................................................................................................

Por la presente confirmo que la información reflejada en esta documentación es veraz

Firma del notificador a la AEMPS

Juan Macías Sánchez

# Anexo 5. FORMULARIO DE NOTIFICACIÓN DE REACCIÓN ADVERSA GRAVE E INESPERADA.

| NOTIFICACION DE SOSPECHA DE  REACCION ADVERSA PARA  MEDICAMENTOS EN INVESTIGACIÓN | CODIGO DE PROTOCOLO (promotor)……………….  Nº EUDRACT/ Nº Protocolo AEMPS……………….. | Nº NOTIFICACION (Promotor) |
| --- | --- | --- |
| Notificación realizada a Eudravigilance SI NO | PACIENTE Nº | Nº NOTIFICACION |

INFORMACION SOBRE LA REACCIÓN ADVERSA

| 1a. PAÍS | 2. FECHA DE NACIMIENTO | | | 2a. EDAD | 3. SEXO | 3a. PESO | 3b. TALLA | | 4-6. FECHA DE INICIO DE LA REACCIÓN | | |
| --- | --- | --- | --- | --- | --- | --- | --- | --- | --- | --- | --- |
|  | DÍA | MES | AÑO |  | HOMBRE  MUJER |  |  | | DÍA | MES | AÑO |
| 7. DESCRIPCIÓN DE LA REACCIÓN ADVERSA (Incluyendo resultados relevantes de exploración o de laboratorio, y la fecha de finalización, si procede). | | | | | | | | 8-13b. CRITERIOS DE GRAVEDAD/ DESENLACE  FALLECIMIENTO  LA VIDA DEL PACIENTE HA ESTADO EN PELIGRO  HOSPITALIZACIÓN  PROLONGACIÓN HOSPITALIZACIÓN  INCAPACIDAD PERMANENTE O SIGNIFICATIVA  RA CLINICAMENTE RELEVANTE  Desenlace  PERSISTENCIA DE LA RA  RECUPERACIÓN SIN SECUELAS  RECUPERACIÓN CON SECUELAS  DESCONOCIDO | | | |

II. INFORMACION DEL MEDICAMENTO EN INVESTIGACIÓN

| 14. MEDICAMENTO SOSPECHOSO | 15. DOSIS  DIARIA | 16. VÍA | 17. ENFERMEDAD EN ESTUDIO | | 18. FECHAS DE  INICIO FINAL | | 19. DURACIÓN DEL  TRATAMIENTO |
| --- | --- | --- | --- | --- | --- | --- | --- |
|  |  |  |  | |  |  |  |
| 20. ¿REMITIÓ LA REACCIÓN AL SUSPENDER LA  MEDICACIÓN?  SI NO NO PROCEDE | | 20a. ¿REMITIÓ LA REACCIÓN AL REDUCIR LA  DOSIS?  SI NO NO PROCEDE | | 21. ¿REAPARECIÓ LA REACCIÓN AL ADMINISTRAR DE NUEVO LA MEDICACIÓN?  SI NO NO PROCEDE | | | |

III. MEDICAMENTOS CONCOMITANTES E HISTORIA CLÍNICA

| 22. MEDICAMENTOS CONCOMITANTES (Márquese con un asterisco el o los medicamentos sospechosos) | 22a. DOSIS  DIARIA | 22b. VÍA | 22c. FECHAS DE  INICIO FINAL | | 22d. MOTIVO DE LA PRESCRIPCIÓN |
| --- | --- | --- | --- | --- | --- |
|  |  |  |  |  |  |
|  |  |  |  |  |  |
|  |  |  |  |  |  |
|  |  |  |  |  |  |
| 23. DATOS IMPORTANTES DE LA HISTORIA CLÍNICA (ej. diagnósticos, alergias, embarazos, etc.) | | | | | |

IV. INFORMACION SOBRE PROMOTOR E INVESTIGADOR

| 24a. NOMBRE Y DIRECCION DEL PROMOTOR | | 24b. NOMBRE Y DIRECCION DEL INVESTIGADOR |
| --- | --- | --- |
| 24c. CODIGO DE LABORATORIO  (Nº AEMPS) | 25a. TIPO DE INFORME  INICIAL  SEGUIMIENTO | 24c. TECNICO DEL PROMOTOR QUE INFORMA  NOMBRE:  TELEFONO:  FIRMA: |
| 24e. FECHA DEL INFORME | 24f. FECHA DE ENTRADA AEM | 25b. SE ADJUNTA INFORME COMPLEMENTARIO |

**INSTRUCCIONES GENERALES**

1. Este formulario se utilizará solamente para comunicar las sospechas de reacciones adversas (RA) graves e inesperadas que ocurran con medicamentos en investigación.

2. Las sospechas de RA mortales o que entrañen riesgo vital (aquellas que de no haber mediado una intervención terapéutica inmediata hubieran supuesto la muerte del paciente) se comunicarán en el plazo máximo de 7 días naturales; si no se dispusiera de toda la información, ésta podrá completarse en el plazo adicional de 8 días. Las demás sospechas de RA graves e inesperadas se comunicarán en el plazo máximo de 15 días.

3. Cuando el espacio disponible sea insuficiente, se añadirá una hoja de información adicional, correctamente identificada con el nombre del promotor y el número asignado a la notificación. En dicha información podrá hacerse constar la evaluación de la causalidad realizada por el técnico que informa.

**INSTRUCCIONES ESPECÍFICAS**

Ø. El código de protocolo es el asignado por el promotor para identificar el ensayo. El número de notificación del promotor es el que éste utiliza para su archivo. Cuando se trate de información de seguimiento se utilizará el mismo número o bien, si se modifica, se indicará el número de la notificación inicial. Se dejará sin rellenar el espacio “Nº de notificación” que aparece sombreado.

2. La edad se pondrá en años, meses, semanas o días según convenga, pero siempre indicándolo. Si no se conoce con precisión la edad debe referirse, al menos, el grupo de edad al que pertenece (p. ej.: lactante, niño, adolescente, adulto, anciano).

7. Se describirá la RA en forma completa, indicando la fecha de finalización de la misma e incluyendo los resultados de las exploraciones complementarias o pruebas de laboratorio que se consideren de interés. A esta notificación podrán acompañarse cuantos informes se estimen convenientes para la adecuada interpretación del cuadro clínico sospechoso de ser una reacción adversa.

8-13. Las categorías no son mutuamente excluyentes. La asistencia en un Servicio de Urgencias de un Hospital inferior a 24 horas, no se considerará hospitalización.

14. Los medicamentos en investigación se identificarán a ser posible por su nombre genérico (DOE o DCI), indicando cuando esté disponible el nombre comercial, o en su defecto, por el nombre propuesto o código de laboratorio para el producto.

15. En caso de que la administración no sea diaria se intentará describirla con alguna de las siguientes posibilidades: cíclica, semanal, mensual, anual o número de veces que se ha utilizado (poniendo en este caso la dosis de cada toma, no la total).

17. Se hará constar el proceso patológico del paciente al que va destinado el producto en investigación, o bien “voluntario sano” en caso de tratarse de tal.

19. Se hará constar la duración del tratamiento hasta el inicio de la reacción adversa.

22. Se indicará explícitamente si no se han tomado fármacos concomitantes. En el caso de considerar sospechoso alguno o algunos de los fármacos concomitantes se marcarán con un asterisco (p.ej.: * AMOXICILINA). Se excluirán los medicamentos utilizados para tratar la reacción adversa.

# Anexo 6. COMPROMISO DEL INVESTIGADOR COORDINADOR

Dr:

del servicio de

del centro

Hace constar:

Que ha evaluado el protocolo del estudio titulado: “ENSAYO CLÍNICO PILOTO, FASE II PARA EVALUAR LA ACTIVIDAD ANTIVÍRICA DE LA COMBINACIÓN DE INTERFERÓN PEGILADO MÁS RIBAVIRINA MÁS NITAZOXANIDA EN INDIVIDUOS CON HEPATITIS CRÓNICA DEBIDA A GENOTIPO 4 DEL VHC Y COINFECTADOS POR VIH”, versión final revisión 2 (Abril 2010), con código de protocolo *Código EudraCT : 2010-024336-42* , cuyo promotor es el Dr. Juan Macías Sánchez.

Y se compromete a:

- Firmar un compromiso en el que se reconoce como investigador del estudio y afirma que conoce el protocolo y cualquier modificación del mismo, y está de acuerdo con él en todos sus términos.
- Informar a los sujetos de investigación y obtener su consentimiento.
- Recoger, registrar y notificar los datos de forma correcta respondiendo de su actualización y calidad ante las auditorias oportunas.
- Notificar al promotor los acontecimientos adversos según se establezca en el protocolo.
- Respetar la confidencialidad de los datos de los sujetos participantes en el estudio.
- Facilitar las auditorias del promotor y las inspecciones de las autoridades sanitarias.
- Saber responder sobre los objetivos, metodología básica y significado de los resultados del estudio ante la comunidad científica y profesional.
- Firmar el protocolo y cualquier modificación del mismo junto con el promotor.
- Co-responsabilizarse con el promotor de la elaboración de los informes de seguimiento y finales.
- Contribuir a difundir los resultados del estudio, en colaboración con el promotor.

Firma del Investigador: ____________________________________________ Fecha: ___/___/___

*Día Mes Año*

# Anexo 7. COMPROMISO DEL INVESTIGADOR

Dr:

del servicio de

del centro

Hace constar:

Que ha evaluado el protocolo del estudio titulado: “ENSAYO CLÍNICO PILOTO, FASE II PARA EVALUAR LA ACTIVIDAD ANTIVÍRICA DE LA COMBINACIÓN DE INTERFERÓN PEGILADO MÁS RIBAVIRINA MÁS NITAZOXANIDA EN INDIVIDUOS CON HEPATITIS CRÓNICA DEBIDA A GENOTIPO 4 DEL VHC Y COINFECTADOS POR VIH”, versión final (Noviembre 2010), con código de protocolo *Código EudraCT : 2010-024336-42*, cuyo promotor es el Dr. Juan Macías Sánchez.

Que acepta participar como investigador principal en este estudio, y se compromete a:

- Firmar un compromiso en el que se reconocen como investigadores del estudio y afirman que conocen el protocolo y cualquier modificación del mismo, y están de acuerdo con él en todos sus términos.
- Informar a los sujetos de investigación y obtener su consentimiento.
- Recoger, registrar y notificar los datos de forma correcta respondiendo de su actualización y calidad ante las auditorias oportunas.
- Notificar al promotor los acontecimientos adversos según se establezca en el protocolo.
- Respetar la confidencialidad de los datos de los sujetos participantes en el estudio.
- Facilitar las auditorias del promotor y las inspecciones de las autoridades sanitarias.
- Saber responder sobre los objetivos, metodología básica y significado de los resultados del estudio ante la comunidad científica y profesional.

Firma del Investigador: ____________________________________________ Fecha: ___/___/___

*Día Mes Año*

# Anexo 8. CONFORMIDAD DEL CEIC

# Anexo 9. FICHA TÉCNICA DEL MEDICAMENTO INVESTIGADO

Se adjunta en documento aparte.

# Anexo 10. HOJA DE INFORMACIÓN AL PACIENTE

**Título:** “ENSAYO CLÍNICO PILOTO, FASE II PARA EVALUAR LA ACTIVIDAD ANTIVÍRICA DE LA COMBINACIÓN DE INTERFERÓN PEGILADO MÁS RIBAVIRINA MÁS NITAZOXANIDA EN INDIVIDUOS CON HEPATITIS CRÓNICA DEBIDA A GENOTIPO 4 DEL VHC Y COINFECTADOS POR VIH”, versión final. Revisión 12 (Abril 2011)

**Código EudraCT:** *2010-024336-42.*

**Versión:** Final. Revisión 2 (Abril 2011)

**Promotor:** Dr. Juan Macías Sánchez. Hospital Universitario de Valme

Estimado paciente,

Nos dirigimos a usted para invitarle a participar en un estudio de tipo ensayoclínico en fase II en el que está previsto incluir un total de 45 pacientes procedentes de 9 hospitales de todo el país que, como usted, estén diagnosticados de coinfección por VIH y genotipo 4 del VHC. El doctor que lleva a cabo el estudio

………………………………………………………………………………………………………………ha determinado que usted reúne los requisitos para poder participar.

Para ello, es necesario que previamente usted reciba la información correcta y suficiente, de este modo podrá evaluar y juzgar si quiere o no participar.

Por favor, lea detenidamente esta hoja informativa con atención, y no dude en ponerse en contacto con su médico para cualquier duda que le pueda surgir acerca de este documento y/o de la información sobre el estudio que le entregamos a continuación.

**Participación en el estudio**

Debe saber que su participación en el estudio es totalmente voluntaria. Usted puede negarse a participar antes o durante el estudio sin que de ello se derive ningún perjuicio, ni que se vea afectada su atención médica o que suponga pérdida de los beneficios a los que usted tiene derecho.

**Objetivo y descripción del estudio**

La nitazoxanida (NTZ) es un fármaco antiparasitario. Recientemente se ha determinado que en pacientes infectados por el genotipo 4 del VHC (VHC-4), sin coinfección por VIH, puede contribuir a aumentar la tasa de respuesta viral sostenida (RVS), que se considera indicativa de curación de la infección por VHC, del tratamiento estándar consistente en interferón pegilado (Peg-IFN) más ribavirina (RBV). Se desconoce si la adición de NTZ al tratamiento estándar actual podría mejorar las pobres tasas de RVS de los sujetos coinfectados por VIH y VHC-4, y si, además, puede incrementar eficacia del retratamiento de pacientes que no han presentado RVS con terapia estándar. Además, no se conocen la seguridad de añadir NTZ a Peg-IFN más RBV en pacientes infectados por VIH que suelen estar tomando fármacos antirretrovirales. Por estas razones, le planteamos participar en este estudio que evalúa la eficacia y seguridad de Peg-IFN más RBV más NTZ en pacientes coinfectados por VIH/VHC-4.

**Procedimientos del estudio**

Su participación en el estudio consistirá en las visitas habituales de todo paciente que recibe tratamiento con Peg-IFN más RBV, es decir, previa al tratamiento, al inicio del tratamiento, a las 4, 12, y cada 12 semanas posteriormente hasta la evaluación de la RVS. En total, para un traatmiento de 48 semanas, se programan 9 visitas. Este ensayo tendrá 14 visitas, 5 más que un tratamiento convencional. Se añaden visitas intermedias para evaluar la seguridad de la combinación Peg-IFN más RBV más NTZ. Las visitas cada 12 semanas coincidirán con sus visitas habituales para controlar la infección por VIH.

En la primera visita se determinará si usted reúne los criterios de inclusión en este ensayo. Posteriormente, en la segunda visita comenzará con la NTZ 500 mg, 1 comprimido cada 12 horas, durante 4 semanas. En la segunda visita, se añadirá Peg-IFN y RBV a las dosis estándar. Seguirá con esta combinación de Peg-IFN más RBV y NTZ durante 52 semanas. En ese momento se suspenderá el tratamiento y se le evaluará a loas 12 y 24 semanas posteriormente para determinar si ha presentado una RVS. Si durante su tratamiento frente al VHC no alcanza los criterios establecidos de respuesta al mismo se suspenderá anticipadamente. Después de esto, su participación habrá finalizado. En cada visita, se recogerán los datos que habitualmente se examinan durante el tratamiento con Peg-IFN más RBV. Se vigilará especialmente la aparición de efectos adversos relacionados con el tratamiento.

**Duración prevista del estudio y número de sujetos participantes**

Se estima que en este ensayo participen 45 sujetos que, como usted, estén diagnosticados de coinfección por VIH/VHC-4. El estudio se realizará en los Servicios de Enfermedades Infecciosas y Medicina Interna de 9 hospitales españoles, y tendrá una duración de 76 semanas.

**Beneficios posibles por participar en el estudio**

Si en los paciente coinfectados por VIH y VHC-4 se obtuviesen incrementos similares en las tasas de RVS a las observadas en los pacientes con infección por VHC-4 sin coinfección por VIH, este tratameinto supondría un beneficio potencial importante. Sin embargo, este hecho es una incognita. Este ensayo se ha diseñado para determinar si existe esa posibilidad y son necesarios ensayos de mayor tamaño.

**Riesgos derivados del estudio**

Desconocemos las posibles interacciones entre Peg-IFN, RBV, fármacos antirretrovirales y NTZ. Se conoce la seguridad de la NTZ añadida al Peg-IFN más RBV, a los que añade muy poca toxicidad. Se conoce la toxicidad de NTZ para tratar infecciones oportunistas de pacientes con sida, en general bien tolerada. Pero no tenemos datos de la seguridad de la NTZ en conjunción con los otros fármacos. Presumiblemente la NTZ se tolerará bien y no son esperables efectos adversos nuevos. En cualquier caso, el ensayo está diseñado para vigilar la aparición de dichos efectos adversos.

**Confidencialidad**

Toda la información que se obtenga durante el estudio será confidencial y ni usted ni sus datos, en ningún caso, estarán identificados en cualquier informe que se emita de este estudio. Estos datos se manejarán de acuerdo con la Ley Orgánica de Protección de Datos de Carácter Personal 15/1999, de 13 de diciembre, teniendo usted los derechos que la citada ley le reconoce de acceso, rectificación, cancelación y oposición de los datos.

Si usted decide participar, sus datos serán accesibles a las Autoridades Sanitarias, Comités Éticos de Investigación Clínica (CEICs), auditores y al Promotor, para la verificación de los procedimientos y datos obtenidos durante el estudio, sin violar la confidencialidad de sus datos. Los datos del estudio podrán ser publicados en revistas científicas pero su identidad permanecerá confidencial. En cualquier caso se cumplirá lo establecido en la Ley Orgánica de Protección de Datos de Carácter Personal 15/1999, de 13 de diciembre.

**Información adicional y teléfono de contacto**

El presente estudio y Consentimiento Informado han sido aprobados por el Comité Ético de Investigación Clínica del Hospital de Valme (Sevilla).

Su médico y/o miembros de su equipo están a su disposición para atender cualquier consulta que usted quiera realizar con relación al procedimiento del estudio. Recibirá una copia de este documento de consentimiento informado y podrá solicitar información adicional contactando con el investigador, Dr.__________________________ en el número de teléfono ___________________.

# Anexo 11. FORMULARIO DE CONSENTIMIENTO INFORMADO

**Título:** “ENSAYO CLÍNICO PILOTO, FASE II PARA EVALUAR LA ACTIVIDAD ANTIVÍRICA DE LA COMBINACIÓN DE INTERFERÓN PEGILADO MÁS RIBAVIRINA MÁS NITAZOXANIDA EN INDIVIDUOS CON HEPATITIS CRÓNICA DEBIDA A GENOTIPO 4 DEL VHC Y COINFECTADOS POR VIH”, versión final. Revisión 2 (Abril 2011)

**Código EudraCT:** *2010-024336-42.*

**Versión:** Final. Revisión 2 (Abril 2011)

**Promotor:** Dr. Juan Macías Sánchez. Hospital Universitario de Valme

Yo, (nombre y apellidos) ____________________________________________ manifiesto que

he sido informado/a del presente estudio y:

- He leído la hoja de información que se me ha entregado.
- He podido hacer preguntas sobre el estudio.
- He recibido suficiente información sobre el estudio.
- He hablado con el doctor_____________________________________________.
- Comprendo que mi participación es voluntaria.
- Comprendo que puedo retirarme del estudio:
- Cuando quiera.
- Sin tener que dar explicaciones.
- Sin que esto repercuta en mis cuidados médicos.
- Comprendo que mi participación en el estudio no conlleva ningún perjuicio para mi salud.
- He sido informado/a de que mis datos personales serán protegidos, que los resultados de mi evaluación personal serán estrictamente confidenciales.
- He recibido una copia firmada de este formulario de consentimiento.

Tomando esto en consideración, OTORGO libremente mi CONSENTIMIENTO a participar en el estudio y a que mis datos puedan ser utilizados con fines de investigación.

__________________________________________ Fecha: ___/___/___

Firma del Participante en el ensayo *Día Mes Año*

__________________________________________ Fecha: ___/___/___

Firma del Investigador *Día Mes Año*

**Anexo 12. FORMULARIO DE CONSENTIMIENTO INFORMADO ORAL ANTE TESTIGOS**

**Título:** “ENSAYO CLÍNICO PILOTO, FASE II PARA EVALUAR LA ACTIVIDAD ANTIVÍRICA DE LA COMBINACIÓN DE INTERFERÓN PEGILADO MÁS RIBAVIRINA MÁS NITAZOXANIDA EN INDIVIDUOS CON HEPATITIS CRÓNICA DEBIDA A GENOTIPO 4 DEL VHC Y COINFECTADOS POR VIH”, versión final. Revisión 2 (Abril 2011)

**Código EudraCT:** *2010-024336-42.*

**Versión:** Final. Revisión 2 (Abril 2011)

**Promotor:** Dr. Juan Macías Sánchez. Hospital Universitario de Valme

Yo, (nombre del testigo) ____________________________________________ declaro bajo mi

responsabilidad que D./Dña. ____________________________________________

- Ha leído la hoja de información que se le ha entregado.
- Ha podido hacer preguntas sobre el estudio.
- Ha recibido suficiente información sobre el estudio.
- Ha hablado con el doctor_____________________________________________.
- Comprende que su participación es voluntaria.
- Comprende que puede retirarse del estudio:
- Cuando quiera.
- Sin tener que dar explicaciones.
- Sin que esto repercuta en sus cuidados médicos.
- Comprende que su participación en el estudio no conlleva ningún perjuicio para su salud.
- Ha sido informado/a de que sus datos personales serán protegidos, que los resultados de su evaluación personal serán estrictamente confidenciales.
- Ha recibido una copia firmada de este formulario de consentimiento.

Tomando esto en consideración, OTORGA libremente su CONSENTIMIENTO a participar en el estudio y a que sus datos puedan ser utilizados con fines de investigación.

_________________________________________ Fecha: ___/___/___

Firma del Testigo *Día Mes Año*

__________________________________________ Fecha: ___/___/___

Firma del Investigador *Día Mes Año*

# Anexo 13. MEMORIA ECONOMICA

**Título:** “ENSAYO CLÍNICO PILOTO, FASE II PARA EVALUAR LA ACTIVIDAD ANTIVÍRICA DE LA COMBINACIÓN DE INTERFERÓN PEGILADO MÁS RIBAVIRINA MÁS NITAZOXANIDA EN INDIVIDUOS CON HEPATITIS CRÓNICA DEBIDA A GENOTIPO 4 DEL VHC Y COINFECTADOS POR VIH”, versión final. Revisión 1 (Enero 2011)

**Código EudraCT:** *2010-024336-42.*

**Versión:** Final. Revisión 2 (Abril 2011)

**Promotor:** Dr. Juan Macías Sánchez. Hospital Universitario de Valme

| **Gastos de Ejecución** |  |
| --- | --- |
| Gastos de material fungible (fungibles de oficina e informáticos)  Subcontratación (CROs)  Seguro  Adquisición/fabricación del MI  (45 pacientes, 16 meses de tratamiento)  Gastos relacionados con la obtención de autorizaciones administrativas y dictámenes  Gastos de publicación y difusión de resultados (Adquisición de separatas de la publicación, impresión de pósters, inscripción en un congreso nacional y un congreso internacional)  Otros gastos:  Gastos de desplazamiento de los pacientes:  (4 desplazamientos adicionales a los asistenciales; 20€/desplazamiento ida y vuelta) | 1000  74810  3500  101038  Solicitada exención  2000  3600 |
| SUBTOTAL | 185948 |
| **Viajes y Dietas** |  |
| Congresos para la presentación de resultados:  Nacional  Internacional | 500  700 |
| SUBTOTAL | 1200 |
| Costes indirectos y de gestión (2%) | 3742,96 |
| **TOTAL** | **190890,96** |

**Promotor Fecha Firma**

Dr. Juan Macías ____________ _____________

# Anexo 14. BIBLIOGRAFÍA

1. Pineda JA, García-García JA, Aguilar-Guisado M, et al. Clinical progression of hepatitis C virus-related chronic liver disease in human immunodeficiency virus-infected patients undergoing highly active antiretroviral therapy. Hepatology 2007; 46: 622-630.
2. Thein HH, Yi Q, Dore GJ, Krahn MD. Natural history of hepatitis C virus infection in HIV-infected individuals and the impact of HIV in the era of highly active antiretroviral therapy: a meta-analysis. AIDS 2008; 22: 1979-1991.
3. Macías J, Berenguer J, Japón MA, et al. Fast fibrosis progression between repeated liver biopsies in patients coinfected with human immunodeficiency virus/hepatitis C virus. Hepatology 2009; 50: 1056-1063.
4. Merchante N, Girón-González JA, González-Serrano M, et al. Survival and prognostic factors of HIV-infected patients with HCV-related end-stage liver disease. AIDS 2006; 20: 49-57.
5. Berenguer J, Alvarez-Pellicer J, Martín PM, et al. Sustained virological response to interferon plus ribavirin reduces liver-related complications and mortality in patients coinfected with human immunodeficiency virus and hepatitis C virus. Hepatology 2009; 50: 407-413.
6. Torriani FJ, Rodriguez-Torres M, Rockstroh JK et al. Peginterferon alfa-2a plus ribavirin for chronic hepatitis C virus infection in HIV-infected patients. N Engl J Med 2004; 351: 438-450.
7. Carrat F, Bani-Sadr F, Pol S et al. Pegylated interferon alfa-2b vs standard interferon alfa-2b, plus ribavirin, for chronic hepatitis C in HIV-infected patients: a randomized controlled trial. JAMA 2004; 292: 2839-2848.
8. Nunez M, Miralles C, Berdun MA et al. Role of weight-based ribavirin dosing and extended duration of therapy in chronic hepatitis C in HIV-infected patients: the PRESCO trial. AIDS Res Hum Retroviruses 2007; 23: 972-982.
9. Pineda JA, Mira JA, Gil IL et al. Influence of concomitant antiretroviral therapy on the rate of sustained virological response to pegylated interferon plus ribavirin in hepatitis C virus/HIV-coinfected patients. J Antimicrob Chemother 2007; 60: 1347-1354.
10. Berenguer J, González-García J, López-Aldeguer J, et al. Pegylated interferon alpha-2a plus ribavirin versus pegylated interferon alpha-2b plus ribavirin for the treatment of chronic hepatitis C in HIV-infected patients. J Antimicrob Chemother 2009; 63:1256-1263.
11. Mira JA, López-Cortés LF, Barreiro P, et al. Efficacy of pegylated interferon plus ribavirin treatment in HIV/hepatitis C virus co-infected patients receiving abacavir plus lamivudine or tenofovir plus either lamivudine or emtricitabine as nucleoside analogue backbone. J Antimicrob Chemother 2008; 62: 1365-1373.
12. Merchante N, Rivero A, de los Santos-Gil I, et al. Insulin resistance is associated with liver stiffness in HIV/HCV co-infected patients. Gut 2009; 58: 1654-1660.
13. Soriano V, Núñez M, Sánchez-Conde M, et al. Response to interferon-based therapies in HIV-infected patients with chronic hepatitis C due to genotype 4. Antivir Ther 2005; 10: 167-170.
14. Martín-Carbonero L, Puoti M, García-Samaniego J, et al. Response to pegylated interferon plus ribavirin in HIV-infected patients with chronic hepatitis C due to genotype 4. J Viral Hepat 2008;15: 710-715.
15. Korba BE, Montero AB, Farrar K, et al. Nitazoxanide, tizoxanide and other thiazolides are potent inhibitors of hepatitis B virus and hepatitis C virus replication. Antiviral Res 2008; 77: 56-63.
16. Rossignol JF, Elfert A, El-Gohary Y, Keeffe EB. Improved virologic response in chronic hepatitis C genotype 4 treated with nitazoxanide, peginterferon, and ribavirin. Gastroenterology 2009; 136: 856-862.
17. Rossignol JF, Elfert A, Keeffe EB. Treatment of chronic hepatitis C using a 4-week lead-in with nitazoxanide before peginterferon plus nitazoxanide. J Clin Gastroenterol 2010; 44: 504-509.
18. Rossignol JF, Elfert A, El-Gohary Y, Keeffe EB. Randomized controlled trial of nitazoxanide-peginterferon-ribavirin, nitazoxanide-peginterferon and peginterferon-ribavirin in the treatment of patients with chronic hepatitis C genotype 4. 43^rd^ EASL. Milán (Italia), 23-27 de abril de 2008. Abstract 68.
19. Sarrazin C, Zeuzem S. Resistance to direct antiviral agents in patients with hepatitis C virus infection. Gastroenterology 2010; 138:447-462.
20. Foster GR, C. Hezode C, Bronowicki JP, et al. Activity of telaprevir alone or in combination with peginterferon alfa-2a and ribavirin in treatment-naive genotype 2 and 3 hepatitis- C patients: final results of study C209. 45^th^ Annual Meeting of the European Association for the Study of the Liver. Viena , 14-18 de abril de 2010. Abstract 57.
21. Benhamou Y, Moussalli J, Ratziu V, et al. Results of a prove of concept study (C210) of telaprevir monotherapy and in combination with peginterferon alfa-2a and ribavirin in treatment-naive genotype 4 HCV patients. 44^th^ EASL. Copenague, 22-26 de abril de 2009. Abstract 10.
