# Supplementary material for: Low Efficacy of Pegylated Interferon plus Ribavirin plus Nitazoxanide for HCV Genotype 4 and HIV Coinfection
Source: PLoS One. 2015 Dec 7;10(12):e0143492. doi: 10.1371/journal.pone.0143492 (PMC4671604; doi:10.1371/journal.pone.0143492)
Supplement: S2 Protocol — (DOCX) [file pone.0143492.s002.docx]

**PHASE II CLINICAL TRIAL TO EVALUATE THE ANTIVIRAL ACTIVITY OF PEGYLATED INTERFERON PLUS RIBAVIRIN PLUS NITAZOXANIDE IN INDIVIDUALS WITH CHRONIC HEPATITIS DUE TO HCV GENOTYPE 4 AND COINFECTED BY HIV**

*Protocol code: NTZSPA001*

*EudraCT code: 2010-024336-42*

**SPONSOR STUDY COORDINATORS**

Dr. Juan Macías Sánchez Dr. Ramón Morillo Verdugo

UGC de Enfermedades Infecciosas UGC de Farmacia

Hospital Universitario de Valme Hospital Universitario de Valme

Seville  Seville

Dr. Juan Macías Sánchez

UGC de Enfermedades Infecciosas

Hospital Universitario de Valme

Seville

#

# TITLE AND VERSIONOF THE PROTOCOL

PHASE II CLINICAL TRIAL TO EVALUATE THE ANTIVIRAL ACTIVITY OF PEGYLATED INTERFERON PLUS RIBAVIRIN PLUS NITAZOXANIDE IN INDIVIDUALS WITH CHRONIC HEPATITIS DUE TO HCV GENOTYPE 4 AND COINFECTED BY HIV

Título corto: EFFICACY OF PEGYLATED INTERFERON PLUS RIBAVIRIN PLUS NITAZOXANIDE IN HCV GENOTYPE 4 AND HIV COINFECTION

Version: 1^st^, revision 2^nd^: 08/06/2011

# STUDY COORDINATORS

Juan Macías Sánchez

UGC de Enfermedades Infecciosas

Hospital Universitario de Valme

Avda. de Bellavista s/n

41014 – Seville

Telf.: 955015757

Fax: 955015461

Ramón Morillo Verdugo

UGC de Farmacia

Avda. de Bellavista s/n

41014 – Seville

Telf.: 955015467

Fax: 955015461

# SPONSOR

Juan Macías Sánchez

UGC de Enfermedades Infecciosas

Hospital Universitario de Valme

Avda. de Bellavista s/n

41014 – Seville

Telf.: 955015757

Fax: 955015461

# SUMMARY

## Sponsor identification and address

Juan Macías Sánchez

UGC de Enfermedades Infecciosas

Hospital Universitario de Valme

Avda. de Bellavista s/n

41014 – Seville

Telf.: 955015757

Fax: 955015461

## Study title

PHASE II CLINICAL TRIAL TO EVALUATE THE ANTIVIRAL ACTIVITY OF PEGYLATED INTERFERON PLUS RIBAVIRIN PLUS NITAZOXANIDE IN INDIVIDUALS WITH CHRONIC HEPATITIS DUE TO HCV GENOTYPE 4 AND COINFECTED BY HIV

## Protocol code

*Protocol code: NTZSPA001*

*EudraCT code: 2010-024336-42*

## Study coordinator, monitor and address

Juan Macías Sánchez

UGC de Enfermedades Infecciosas

Hospital Universitario de Valme

Avda. de Bellavista s/n

41014 – Seville

Telf.: 955015757

Fax: 955015461

Ramón Morillo Verdugo

UGC de Farmacia

Avda. de Bellavista s/n

41014 – Seville

Telf.: 955015467

Fax: 955015461

## Centers where the study is planned to be carried oput

Nine Spanish hospitals where patients coinfected by HIV/HCV are followed.

## Ethics committee

The study will be submitted for assessment to the Regional Committee Clinical Trial of Andalusia (Comité Autonómico de Ensayos Clínicos de la Comunidad Autónoma de Andalucía) and Madrid.

## Main objective

To assess the SVR rate of treatment with Peg-IFN alfa-2b plus RBV and NTZ in patients coinfected with HIV and HCV genotype 4, never exposed to treatment or failure to prior standard treatment with Peg-IFN plus RBV, and compared with the SVR rate obtained in these patients with Peg-IFN plus RBV in a historical cohort.

## Design

Pilot clinical trial, with a single arm, to evaluate efficacy and safety (phase II).

## Disease or condition under study

Coinfection with HIV and HCV genotype 4.

## Drugs under study

Nitazoxanide 500 mg every 12 hours for 4 weeks followed by nitazoxanide 500 mg every 12 hours plus pegylated interferon alfa-2b 1.5 mcg/kg/week and weight-adjusted ribavirin for 48 weeks.

## Study population and global number of subjects

Patients infected with HIV-1 with chronic hepatitis due to HCV genotype 4 who meet the selection criteria.

Number of patients to be included in the study: 45.

## Calendar

The following timetable is foreseen (in total the study will last 12 months from the inclusion of the last patient's first 18 months of follow-up). The times described in this section may be modified by the terms of the administrative process of commissioning the study:

- Start of study: AEMPS approval pending.

- Period of inclusion: AEMPS approval pending.

- Follow-up period: AEMPS approval pending.

- Data collection: AEMPS approval pending.

- Statistical analysis: AEMPS approval pending.

## Source of funding

The developer, Dr. Juan Macias, guarantees non-interference in the selection process of cases, analysis of information and / or presentation of results, or any other process that may affect the study results.

Funding for this study come from the 2010 Aid to Promote Independent Clinical Research of the Ministry of Health, Social Policy and Equality (file EC10-187). The funding will in any case independent of the study results.

# INDEX

1 TITLE AND VERSIONOF THE PROTOCOL 2

2 STUDY COORDINATORS 2

3 SPONSOR 2

4 SUMMARY 3

4.1 Sponsor identification and address 3

4.2 Study title 3

4.3 Protocol code 3

4.4 Study coordinator, monitor and address 3

4.5 Centers where the study is planned to be carried oput 4

4.6 Ethics committee 4

4.7 Main objective 4

4.8 Design 4

4.9 Disease or condition under study 4

4.10 Drugs under study 4

4.11 Study population and global number of subjects 4

4.12 Calendar 5

4.13 Source of funding 5

INDEX 6

5 WORKING PLAN 8

6 OBJECTIVES 10

6.1 Primary objective 10

6.2 Secondary objectives 10

7 REVIEW OF BIBLIOGRAFY 10

7.1 Background 10

7.2 Study rationale 12

8 METHODS 12

8.1 Design 12

8.2 Study population 13

8.2.1 Selection criteria 13

8.2.2 Exclusion criteria: 13

8.2.3 Discontinuation criteria: 15

8.3 Description of treatment and exposure 15

8.4 Source of information and setting 15

8.5 Study variables 15

8.5.1 Primary variable 15

8.5.2 Secondary variables 16

8.5.3 Variables explicativas o covariables 16

8.6 Sample size justification 16

8.7 Methods to obtain the data 17

8.8 Data management 17

8.9 Data analysis 17

8.9.1 Specific statistical analysis considerations 18

9 STUDY DRUGS 19

9.1 Investigational drug 19

9.2 Study drugs dispensation 19

9.3 Reception, storing and control of study drugs 20

9.4 Traceability 21

9.4.1 Trazabilidad medicación dispensada 21

9.5 Dose adjustment and discontinuation of drugs 21

9.5.1 Nitazoxanide 21

9.5.2 Reference treatment 22

9.5.3 Disallowed concomitant treatments 24

9.5.4 Study termination and post-trial treatment 27

10 Study termination before the schedule date 27

11 Safety 29

11.1 Physical examination 29

11.2 Vital signs 29

11.3 Body height and weight 30

11.4 Laboratory evaluations 30

11.5 Laboratory tests 30

11.6 Pregnancy and fertility 31

11.7 Other tests to evaluate safety 32

12 ADVERSE EVENTS 35

12.1 Intensity 36

12.2 Relationship with the study drugs 36

12.3 Adverse reaction 37

12.4 Notificación de los acontecimientos adversos graves 38

12.5 Pregnancy 40

12.6 Data and safety monitoring board 41

13 ETHICAL ASPECTS/PROTECTION OF THE PARTICPATING SUBJECTS 42

13.1 Risk-benefit assessment 42

13.2 Information sheet and consent form 42

13.3 Confidentiality of data 43

13.4 Responsibilities of the investigator and the Ethics Committee 44

14 PLAN OF DISEMINATION OF RESULTS 45

15 FUNDING 47

16 AMENDMENTS TO THE PROTOCOL 48

16.1 Adherence to protocol 48

17 PRACTICAL CONSIDERATIONS 48

b. Dissemination of results 49

c. Responsibilities promoter 49

d. Researcher's Responsibilities 50

e. Responsibilities of the coordinator researcher 50

18 REFERENCES 50

# WORKING PLAN

Once the approval of the study by the Ethics Committee and the Health Authorities of the Autonomous Communities involved is obtained, the study will start in the participating institutions, after giving information of the study to the managers of the centers. The participating researchers will be solely responsible for the inclusion of patients in the study after confirming that they meet the selection criteria stated therein. Each patient who is invited to participate in the study will be informed about it both verbally and in writing, giving a document entitled "Patient Information Sheet". No patient will be included in the study until he has been duly informed by the investigator and have freely consented to participate in it, either in writing or orally before independent witnesses of the research team. Upon acceptance by the patient, we will proceed to start collecting data by filling an electronic database that will be provided by the developer, for registration of the information available in the patient chart and data provided by himself. The inclusion of patients will end when the intended sample size is reached. Described below planned visits and variables to collect in each:

| **Evaluation** | **Weeks (Visits)** | | | | | | | | | | | | | |
| --- | --- | --- | --- | --- | --- | --- | --- | --- | --- | --- | --- | --- | --- | --- |
|  | **Screening**  **(V1)** | **0 (V2)** | **4 (V3)** | **8 (V4)** | **12 (V5)** | **16 (V6)** | **20 (V7)** | **24 (V8)** | **28 (V9)** | **36 (V10)** | **44 (V11)** | **52 (V12)** | **64 (V13)** | **76 (V14)** |
| Study procedures |  |  |  |  |  |  |  |  |  |  |  |  |  |  |
| Informed consent |  | x |  |  |  |  |  |  |  |  |  |  |  |  |
| Medical history and examination | x | x | x |  | x |  |  | x |  | x |  | x |  | × |
| Vital signs | x | x | x | x | x | x | x | x | x | x | × | x | × | × |
| Intervention |  |  |  |  |  |  |  |  |  |  |  |  |  |  |
| NTZ monotherapy |  | x | x |  |  |  |  |  |  |  |  |  |  |  |
| NTZ+PegIFN+RBV |  |  | x | x | x | x | x | x | x | x | x | x |  |  |
| Tests |  |  |  |  |  |  |  |  |  |  |  |  |  |  |
| Pregnancy Test (urine or serum) | x | x | x | x | x | x | x | x | x | x | x | x | x | x |
| Plasma HIV RNA | x | x |  |  | x |  |  | x |  | x |  | x |  | x |
| CD4+ and CD8+ cell counts | x | x |  |  | x |  |  | x |  | x |  | x |  | × |
| Blood cell counts | x | x | x | x | x | x | x | x | x | x | × | × | × | × |
| Blood tests | x | x | x | x | x | x | x | x | x | x | × | × | × | × |
| Lipids |  | x | x |  | x |  |  | x |  | x |  | x |  | x |
| Urine analysis | x | x | x | x | x | x | x | x | x | x | × | × | × | × |
| Thyroid tests | x | x | x | x | x | x | x | x | x | x | x | x | x | × |
| HOMA |  | x |  |  |  |  |  |  |  |  |  | x |  | x |
| IL28B |  | x |  |  |  |  |  |  |  |  |  |  |  |  |
| Imaging tests and other |  |  |  |  |  |  |  |  |  |  |  |  |  |  |
| Liver fibrosis assessed by transient elastometry |  | x |  |  |  |  |  |  |  |  |  |  |  | x |
| EKG |  | x |  |  | x |  |  | x |  |  |  | x |  | x |
| Efficacy measurements |  |  |  |  |  |  |  |  |  |  |  |  |  |  |
| Plasma HCV RNA | x | x | x | x | x | x | x | x | x | x | x | x | x | × |
| Compliance evaluation |  |  | x | x | x | x | x | x | x | x | × | × |  |  |
| Safety measures |  |  |  |  |  |  |  |  |  |  |  |  |  |  |
| Adverse events |  |  | x | x | x | x | x | x | x | x | × | × |  |  |
| Concomitant drugs | x | x | x | x | x | x | x | x | x | x | × | × | × | × |

# OBJECTIVES

## Primary objective

To evaluate the rate of sustained virological response (SVR) of pegylated interferon alfa­2b (Peg-IFN) plus ribavirin (RBV) plus nitazoxanide (NTZ) in patients coinfected by HIV and HCV genotipe 4 (HCV‐4), never treated before (naïve) and with a treatment failure to a standard therapy with Peg-IFN plus RBV (experienced), and to compare it with the rate of SVR of these patients with Peg-IFN plus RBV in a historical cohort.

## Secondary objectives

- To evaluate the virological activity at weeks 4 and 12 after starting the combination of Peg-IFN plus RBV plus NTZ in HIV/HCV-4-coinfected patients, in naive as well as in experienced patients.
- To analyze the safety of Peg-IFN plus RBV plus NTZ in HIV/HCV-4-coinfected patients, In naive, as well as in experienced patients

# REVIEW OF BIBLIOGRAFY

## Background

The most effective measure to prevent the progression of chronic hepatitis C in HIV co-infected subjects is the treatment against HCV. Coinfected individuals achieve a sustained viral response (SVR) to combination therapy with pegylated interferon (Peg-IFN) plus ribavirin (RBV) have a lower probability of liver decompensation and death from liver disease than those who do not achieve SVR. However, the proportion of patients coinfected with HIV/HCV who achieve an SVR to HCV treatment is low compared with HCV-monoinfected subjects (1-4). In clinical trials, between 27% and 50% of coinfected subjects show SVR (1,2). In real life conditions, in studies of cohorts of HIV/HCV-coinfected patients, SVR was observed globally between 31% and 37% of patients (3,4).

HCV genotype 4 (HCV-4) is one of the least studied, despite causing 20% of the 170 million cases of chronic hepatitis C worldwide. It is most common in the Middle East and Africa, but in the recent 20 years it has spread to areas such as Greece, Italy, France and Spain. In southern Europe it has been linked to injection drug use, so that patients coinfected with HIV and HCV in our area is not a rare genotype. About 15% of HIV/HCV-coinfected patients in Spain are carriers of HCV-4 (5). We have little information on the response to treatment with Peg-IFN plus RBV in subjects coinfected by HIV and HCV-4. In our area, they behave as difficult to treat patients, with rates of SVR to the combination of Peg-IFN plus RBV between 17% and 28% (5).

Nitazoxanide (NTZ) is a drug originally developed as anti-parasite that has shown activity against HCV (6). In a clinical trial in subjects not previously exposed to anti-HCV drugs and infected with HCV-4, the arm that included a pretreatment for 12 weeks with NTZ followed by a course of treatment with Peg-IFN more NTZ plus RBV showed SVR rates significantly higher than the arm that received standard treatment, 79% vs. 50%, respectively (7). Subsequently, it was demonstrated that pretreatment with NTZ monotherapy for 4 weeks is as effective as pretreatment for 12 weeks (8). Experience in subjects infected with HCV-4 that failed, including non-response and relapse, to Peg-IFN plus RBV is limited (18). Patients infected with HCV-4, with lack of SVR to a first treatment with Peg-IFN plus RBV, who received NTZ plus standard treatment had higher SVR rates than those subjects retreated with Peg-IFN plus RBV, 25% vs . 8%, respectively (9).

In the short term, a very large volume of patients coinfected with HIV and HCV without SVR to Peg-IFN plus RBV could develop liver decompensations, a situation in which treatment options are very limited. Moreover, patients currently infected with HCV-4 without SVR to Peg-IFN plus RBV have no realistic opportunities of being retreated with the new molecules directly active against HCV (DAV) that are being developed. The DAV have been evaluated in experimental models and clinical studies designed to inhibit genotype 1 strains. Therefore, DAV directed toward more variable regions of HCV are more likely to be ineffective in genotypes other than 1. These include inhibitors of HCV NS3 protease, the DAV more advanced in their development. Specifically, telaprevir has a virtually absent antiviral activity against genotype 3 and 4. Boceprevir has a very low antiviral activity against genotypes 2 and 3 (19). A similar behavior is expected for inhibitors non-nucleoside NS5B polymerase. For these reasons, the only therapeutic alternative that would allow to retreat on time patients infected by HCV-4 with an initial treatment failure to Peg-IFN plus RBV, especially those coinfected with advanced fibrosis, would be the use of NTZ.

## Study rationale

It is unknown whether the addition of NTZ the current standard treatment may improve the poor SVR rates of subjects coinfected by HIV and HCV-4, and if, additionally, it may increase the efficacy of a retreatment of patients who have not shown SVR with standard therapy. For these reasons, we should plan studies examining the efficacy of Peg-IFN plus RBV plus NTZ in patients coinfected HIV/HCV-4.

# METHODS

## Design

Single arm pilot clinical trial to evaluate safety and efficacy (phase II)

Study arms:

Nitazoxanide 500 mg every 12 hours for 4 weeks followed by nitazoxanide 500 mg every 12 hours plus pegylated interferon alfa-2b 1.5 mcg/kg/week and weight-adjusted ribavirin for 48 weeks.

Study setting:

Infectious Diseases Units in nine centers for the National Health System, 8 Andalusian centers and one center from Madrid.

## Study population

### ***Selection criteria***

- **Inclusion criteria:**

1. HIV infection.
2. Infection with HCV genotype 4.
3. No prior treatment with any interferon or no response to a previous treatment with Peg-IFN plus RBV. The lack of response will include both nonresponders, and those who showed relapse.
4. Stable antiretroviral therapy 24 weeks before starting the study drugs, with undetectable plasma HIV RNA during that period of time.
5. Commitment to use two non-hormonal contraception during the study and up to 24 weeks after treatment.
6. Acceptance to give written informed consent to participate in the trial.
7. (Anexo 1).

### ***Exclusion criteria:***

1. Antiretroviral therapy including didanosine, stavudine, zidovudine and abacavir.
2. Decompensated cirrhosis.
3. Presence of other significant liver diseases, including chronic hepatitis or acute hepatitis B, acute hepatitis hepatitis A, hemochromatosis or deficiency of alpha-1 antitrypsin.
4. Pregnancy and lactation.
5. Men planning pregnancy with their partners during the study and up to 24 weeks after treatment.
6. Active or uncontrolled depression, other psychiatric illness, or disease during the previous year which may, in the investigator's opinion, prevent participation in the study.
7. Previous suicide attempt.
8. Active thyroid disease or poorly controlled with treatment.
9. Previous autoimmune diseases such as inflammatory bowel disease, psoriasis serious, or rheumatoid arthritis, which may be exacerbated by interferon.
10. Chemotherapy or immunomodulatory 24 weeks before starting the study.
11. Serious illness, including cancer or unstable coronary disease, 24 weeks before starting the study.
12. Any chronic disease which, in the opinion of the investigator, may prevent complete the study.
13. Presence of acute or active opportunistic infections 48 weeks before starting the study.
14. Evidence of hepatocellular carcinoma or alpha-fetoprotein levels ≥ 50 ng / ml, unless an imaging technique shows no evidence of liver tumor, all obtained 24 weeks before starting the study.
15. Hemoglobinopathy or other conditions that may facilitate hemolysis.
16. Solid organ or bone marrow transplant.
17. Known hypersensitivity to any of the drugs under study.
18. Active consumption of drugs or alcohol in the opinion of the investigator would interfere with participation in the study. The use of methadone or other opiate replacement therapy is not considered an exclusion criterion.
19. Serious side effects from treatment with Peg-IFN plus RBV in patients with prior failure of such treatment.

### ***Discontinuation criteria:***

1. Impossibility to follow the trial scheduled visits.
2. Evidence of less than 70% adherence of the planned study drug dosing.
3. Patients own decision or decision of the investigator.

## Description of treatment and exposure

All patients included in this trial will receive nitazoxanide 500 mg every 12 hours for 4 weeks followed by nitazoxanide 500 mg every 12 hours plus weight-adjusted ribavirin and pegylated interferon alfa-2b 1.5 mcg / kg / week for 48 weeks. Standard stopping rules will be applied. Patients will be followed until they reach 72 weeks of follow-up since they started the trial medication.

## Source of information and setting

The source of information will be in all cases the medical records and the patient himself. The information will be collected prospectively at the participating hospitals by including the data in the CRD of the study.

The setting is tertiary care, and Services Infectious Diseases and Internal Medicine of 9 hospitals in the autonomous regions of Andalusia and Madrid will participate.

## Study variables

###

### ***Primary variable***

Achievement of SVR, defined as the proportion of patients with HCV RNA ≤10 IU/ml 24 weeks after finishing the programmed length of treatment.

### ***Secondary variables***

1. The frequency of patients with HCV RNA ≤10 IU/ml 12 weeks after finishing the programmed length of treatment.
2. The proportion of patients with HCV RNA ≤10 IU/ml at 4 and 12 weeks after starting PegIFN plus RBV.
3. The proportion of patients with grade 3 or 4 adverse events according to the WHO classification.

### ***Variables explicativas o covariables***

The following variables potentially associated with treatment response to HCV will be included in this study: Gender, age, route of acquisition of HCV infection, body mass index, CD4 cell count, HIV viral load, ART, presence of previous AIDS, insulin resistance determined by HOMA, LDL cholesterol, HCV viral load, liver fibrosis, genotype of interleukin 28B (IL28B) and adherence to treatment. In NR subjects, if they had a null response, ie falling HCV RNA at week 12 was <2 log10, or had relapsed after the end of the first treatment will be considered. For the analysis of adverse effects the presence of general symptoms, psychiatric disorders, skin disorders, visual disturbances, cardiac toxicity, decompensated cirrhosis, signs of mitochondrial toxicity, autoimmune diseases, thyroid disorders, digestive symptoms, weight, hemoglobin levels, leukocytes, platelets, ALT, AST, GGT, alkaline phosphatase, bilirubin and creatinine will be collected.

## Sample size justification

For individuals with no previous interferon treatment, an SVR rate approximately 30% higher than that observed with standard therapy could be achieved, assuming a similar behavior to that of the HCV-monoinfected subjects (Reference 16). Including 49 patients without treatment, an accuracy of 10% would be achieved, with a normal asymptotic bilateral confidence interval of 95%, assuming that the proportion of subjects with SVR is 50% and that approximately 100 candidates will be available in the participating centers. The relapse rate is estimated to be 5%, so that at least 55% (95% confidence interval: 40%-69%) patients should achieve at the end of treatment response. If no increase in SVR to treatment with nitazoxanide plus PegIFN and RBV compared to the results observed in the HEPAVIR cohort is observed the in interim analysis, the trial may be terminated early.

In the case of subjects with no previous SVR, an SVR rate of 25% could be estimated from HCV-monoinfected patients data (Reference 18). Including 15 patients without previous SVR, an accuracy of 20% would be achieved with a normal asymptotic bilateral confidence interval of 95%, assuming that the proportion of individuals with SVR is 20%. The sample size calculation was performed using the software package Ene 3.0 (e-Biometrics, Madrid, Spain)

## Methods to obtain the data

## Data management

In order to ensure the confidentiality of the survey data, only will have access to them the researcher and his team, the sponsor or the person designated by him, the CEIC, the relevant health authorities and those responsible for the analysis the same.

The content of the case report data and documents generated during the study and the database will be protected from uses not permitted by persons outside the research and therefore will be considered strictly confidential and not be disclosed to third parties.

The processing of personal data required in this study is governed by Law 15/1999 of December 13 of Protection of Personal Data.

## Data analysis

Upon completion of the study, after recording data of the last visit of the last patient included in it, we will proceed to close the database, and it will be transferred to the sponsor, responsible for statistical analysis.

The proposed methods of statistical analysis shown below is a summary of the methods to be used on the data collected, to meet the objectives of the study.

Data of the population of patients who meet the selection criteria of the study will be analyzed.

A general descriptive analysis of the variables included in the study will be done. Distributions of absolute and relative frequencies of qualitative variables and measures of central tendency and dispersion (typical average maximum deviation, median, minimum and) of quantitative variables are presented. The confidence intervals will be presented at 95% for the major quantitative outcome variables associated with the main objective and the main secondary endpoints.

Data will not be imputed in the absence of information and will be considered missing. If a particular subgroup of patients is of interest, sub-analysis of these groups in line with the analysis set for the general population may be made.

### ***Specific statistical analysis considerations***

SVR rates obtained by the naive subjects enrolled in the trial will be compared with those observed in subjects with genotype 4 included in the SAEI HEPAVIR cohort, a cohort of coinfected patients whose first treatment against HCV was Peg-IFN plus RBV. For this comparison, only the patients included in the cohort HEPAVIR SAEI between January 2007 and January 2010 will be considered. The SVR rates in patients without response to treatment with Peg-IFN plus RBV enrolled in the trial will be compared with those of patients without response to this combination included in previous studies (Crespo M, et al J Antimicrob Chemother 2008; 62:. 793-796; Labarga et al J Acquir Immune Defic Syndr 2010; 53:. 364-368). Comparing SVR rates will be achieved through the test of Chi-square. SVR rates of subjects enrolled in the trial will be determined by per protocol and by intention to treat analysis. A descriptive analysis of possible partnerships with SVR in both previously untreated subjects as in the NR analysis will be conducted. If deemed appropriate, independent associations through logistic regression models were examined. Statistical analysis was performed using the SPSS 15 (SPSS Inc., Chicago, IL, USA) and STATA SE 9 (StataCorp, College Station, TX, USA).

# STUDY DRUGS

## Investigational drug

The investigational drug, nitazoxanide 500 mg, will be delivered to the centers participating in the study in packs of 60 tablets. The labeling shall be applied individually for each patient. The label will be facilitated by Distefar del Sur with the features requested by the promoter.

The RBV and Peg-IFN alfa-2b will be purchased locally (commercial packaging). The RBV will be delivered in bottles of 168 tablets of 200 mg. Peg-IFN alfa-2b prefilled pens that contain 50, 80, 100, 120 or 150 mg of Peg-IFN alfa-2b in 0.5 ml will be given for sc injection.

## Study drugs dispensation

The person responsible of the trial from each participating center Pharmacy Service will identify the study drug to be dispensed to each patient. Immediately before dispensing the study drugs to the patient, the research staff will document the specific number of the patient in the detachable part of the study label. Then, the detachable part is removed and pasted into the CRD.

The researcher will explain carefully to the patient the instructions for the proper administration of nitazoxanide, Peg-IFN alfa-2b and RBV. Instructions will be also given to complete a journal at home to record daily doses (nitazoxanide and RBV) and weekly doses (Peg-IFN alfa-2b). The instructions for storage of the study drugs wiil appear in the labeling. Until dispensed, drugs will be stored at each participating center, always with the original packaging and in a safe place.

## Reception, storing and control of study drugs

The study drugs will be received at the participating center by the designated person. At all times, the packages will be handled and stored properly and safely. Storage is done in a safe place which can only be accessed by the researcher and collaborators designated by him. Upon receipt, all study drugs should be stored according to the instructions on the label. Supplies any clinical material will be made only according to what indicated in the protocol.

Each center pharmacist will dispense the investigational drug for each patient depending on the progress of treatment. Pharmacist from each participating center will be given a manual with detailed information on how to dispense the medication. Patients will be given a sufficient amount of investigational drug and the material necessary for self-administration in order that they can be given all scheduled doses between visits. In addition to supplying drugs, each patient will be given a manual with detailed information. The labeling of medication will be in Castilian and comply with current regulations. Labeling storage instructions will be included. Patients will be required to return on each visit any unused medication and containers and packaging of medication. In each visit, medication and the needed equipment will be dispensed.

The investigator must keep accurate records of medication received at the participant center and dispensed to patients, using a ledger of medication. The control and monitoring of accounting medication will be made by the field monitor during his visits to the center and end of study. Patients will be requested to return the medication and materials, used or not, the end of the study or in case of premature withdrawal.

Upon completion of the study and, if necessary, during its development, the researcher will return to the promoter any unused medication, as well as containers and packaging of medication has been used, the packages, labels and a copy the ledger of medication.

## Traceability

Traceability of the drugs supplied for the trial will be guaranteed with the delivery note where the description of the product, number of units, lot and expiry date, that will be issued in triplicate bound for Hospital Pharmacy, will appear. Two of the units will be sealed upon delivery by the pharmacy to the conformity of the delivery and subsequent custody by the Distefar del Sur echnical Director for a period of fifteen years as required by law for the drug.

### *Trazabilidad medicación dispensada*

The traceability of the study drugs will be ensured with the "Registry dispensing patients", mandatory in every dispensation, that will include paragraphs (patient identification, identification of drug dispensed amounts, Lot No. dispensed in each dispensing and expiration date, return date and remaining amount). In this way, it will be recorded which patient has been dispensed a given lot and on what date this dispensation was made.

This record will be complementary to that usually carried out by Pharmacy Services for clinical trials drugs according to RD 223/2004.

Patients will be asked to return on each visit any unused medication and containers and packaging of medication used. In each visit, medication and the needed equipment will be dispensed.

## Dose adjustment and discontinuation of drugs

### Nitazoxanide

No individual adjustment of the dose of the investigational drug will be carried out, according to the information given in the package insert of the product currently available.

### Reference treatment

In patients treated with Peg-IFN alfa-2b is often observed fever, depression and decreased certain hematological parameters (mainly leukocytes and platelets). It is common in clinical practice to adjust the dose of Peg-IFN alfa-2b and RBV based on the results of the complete blood count (CBC) and platelet count. A summary of the guidelines for dose modification of the standard treatment is shown in Table 1. If a patient has a platelet count <50,000 / mm3, hemoglobin <8.5 g / dl or a neutrophil count <750 mm3, the investigator must notify the sponsor. The sponsor, in turn, will give appropriate guidance on dose adjustments in case of other toxicity of grade 3 or 4 and the possible discontinuation of treatment in an individual patient.

**Summary of guidelines for dose modification of the reference treatment:**

The following summarizes the recommendations contained in the package insert of Peg-IFN alfa-2b:

Treatment with Peg-IFN alfa-2b is associated with a decrease in leukocyte counts, neutrophils, lymphocytes and platelets, which generally occurs within the first 2 weeks of treatment. When it is necessary to make a change in dose due to moderate to severe adverse events (clinical and/or laboratory), the reduction the initial dose will be 1 mg/kg/week. If a second reduction is needed, this will be 0.5 mg/kg/week. Once adverse reactions have improved, the dose can be increased again. Guidelines for dose modification of Peg-IFN alfa-2b be those set out in the technical specifications of the drug and the consensus document on the management of HCV infection in HIV-infected patients HEPAVIR group of the Andalusian Society of Infectious Diseases (SAEI) (Table 1). The main toxicity associated with ribavirin is hemolytic anemia. If it is necessary to reduce the doses of RBV, reductions will be made in 200 mg steps daily. The guidelines for dose adjustment of RBV come from its package insert and the consensus document on the management of HCV infection in HIV-infected patients by HEPAVIR SAEI group (Table 1).

**Table 1.** Guidelines for the management of hematologic toxicity associated with treatment with Peg-IFN alfa-2b plus RBV.

| Hematological alteration | Level | Recommendation |
| --- | --- | --- |
| Neutropenia |  |  |
|  | >500 cel/ml | Monitoring |
|  | <500 cel/ml | Start G-CSF 300 mg 2 times/week  Peg-IFN dose reduction if G-CSF not effective |
| Anemia |  |  |
|  | >10 g/dl | EPO 40000 UI/week and/or packed red blood cells transfusion if:   - Drop >4 g/dl during first 4 weeks or - Decompensation of previous chronic disease |
|  | 8-10 g/dl | 1. EPO 40.000 UI/week. 2. EPO 60000 UI/week if after 2 weeks with EPO no increase in Hb ≥1 g/dl is seen. 3. RBV reduction by 200 mg/day (evaluation in 1 week) if with EPO no Hb ≥10 g/dl is achieved. |
|  | <8 g/dl | Depending on symptoms:   1. RBV gradual dose redction. 2. Temporary or definitive RBV discontinuation. 3. Red blood cells transfusion. |
| Trombopenia |  |  |
|  | <30000/ml | Peg-IFN dose reduction* |
|  | <20000/ml | Peg-IFN discontinuation* |

* The level of platelets at baseline, the rate of decline and clinical manifestations will be considered.

**Rescue drugs**

No rescue drugs are allowed during the study.

**Other concomitant treatments**

The administration of disallowed concomitant medications may cause the patient to be excluded from the study. Decisions on the exclusion of patients who require concomitant treatment will be taken with the participation of the sponsor of the study and always depending and the circumstances of each case.

Specific drugs against HCV in prior to the administration of the first dose of the study drugs three months are not allowed.

An agreement between researcher and sponsor is required, before inclusion of patients in the study, on the use of drugs not included in Table 2, as long as they will not be expected to influence the results of the study.

All medications, both prescribed and bought over the counter, including vitamin supplements, taken within 28 days prior to baseline or during the trial, must be registered at the drug/non-drug treatments CRD page. Patients should be asked specifically for the use of herbal products and vitamin supplements. Medication for fever or muscle pain (i.e. paracetamol) is allowed during treatment with Peg-IFN, but the dose should not exceed 3 g/day and should be documented.

### Disallowed concomitant treatments

With regard to drugs that, according to information contained in their package insert, are contraindicated when administered with cyclosporine A or interfere with the absorption, distribution, metabolism or excretion of cyclosporin A, the researcher should consult with the sponsor. The authorization of the sponsor before the patient is admitted to the study is required.

The investigator will explain to patients that they must notify the participating center any start of a new medication once the study begins. All relevant drugs and non-drug therapies (including physiotherapy and blood transfusions) which are initiated after the administration of the first dose of study drug must be registered with the drug section/concurrent non-pharmacological treatment of CRD.

The drugs should be shown in the CRD by trade name, dosage, frequency and route of administration. Dates of start and end of treatment and why the patient received the drugs will be also recorded.

**Tabla 2.** Tratamientos concomitantes no permitidos

| **Drus** | **Duration** |
| --- | --- |
| Antivirals  (HIV therapy not included) | From three months before the first dose of study medication until the final visit |
| Immunesuppressors | From three months before the first dose of study medication until the final visit |
| Phytotherapy  (i.e. St John's wort...) | From three months before the first dose of study medication until the final visit |

**Treatment suspension with the study drugs and leaving the study before completion**

Discontinuation of the study drugs means the permanent interruption of nitazoxanide and Peg-IFN alfa-2b and RBV. Treatment with the study drugs should be suspended in an individual patient when the investigator determines that further treatment would entail a significant risk to patient safety.

In the event that a serious adverse event (SAE) takes place, the sponsor will assess the safety and immediately guide the research on treatment discontinuation and premature withdrawal from the study. The following circumstances may require discontinuation of treatment:

• Request for suspension by the sponsor after reviewing safety data on a treatment group.

• Pregnancy

• Appearance of unacceptable toxicity, although decreasing the dose and/or temporarily stopping treatment.

• Use of the following treatments/banned drugs:

- Chronic treatment with systemic corticosteroids (prednisone equivalent of> 10 mg / day for more than two weeks).
- Other investigational drugs already on the market or to treat infection caused by HCV (p. eg., Peg-IFN alfa-2a, IFN / RBV)
- Any other drug in different research study.

• Withdrawal of informed consent.

• Less than 70% of doses of treatment in any of the compliance monitoring visits.

• Any other protocol violation that results in a significant risk to patient safety.

In addition to these standards and requirements for discontinuation of treatment, the researcher can study discontinuation in an individual patient, if, after considering the "pros" and "cons", concludes that further treatment It would be detrimental to the welfare of the patient.

The section on discontinuation of treatment of the CRD must be completed, indicating the date and the main reason why the treatment is stopped.

The patients whose treatment is stopped should not be considered patients who withdraw from the study before its completion. If a patient is removed from the study, the final evaluation of the study should be conducted. In the case of patients who are lost to follow up (ie, those whose status is unclear because they come to study visits, nor have told the research staff their desire to withdraw from the study), the researcher must make everything in its power to contact the patient, recorded in the source documents the steps it has taken (p. eg., dates on which she called the patient by telephone, certified letters sent to your home address, etc.) .

Patients in the study treatment will not be replaced by others recruited to replace suspensions

.

### Study termination and post-trial treatment

The period of enrollment of patients in the study will end when the sample size of the study is completed or if the study is interrupted before the scheduled date. If a patient is in the selection period once the study is over, he will remain eligible to participate in it.

For each patient, the study is considered terminated when the follow-up visit to assess the safety at week 72 is carried out.

If the patient has withdrawn prematurely from the study, participants will have to return to the center for the final study visit. The researcher must provide adequate follow-up medical care to all patients who have withdrawn prematurely from the study or to refer them to other professionals to continue with adequate medical care.

# Study termination before the schedule date

The study may be suspended until the sponsor can conduct a more detailed assessment if so requests on the basis of pre-established safety criteria. In addition, the study may be terminated at any time and for any reason if so decided by the sponsor and communicated for the approval of the relevant ethics committee. If it was necessary to make this decision, patients should be visited as soon as possible and treated in accordance with the provisions of Section “Premature withdrawal of a patient”. The researcher can receive information about other procedures to be performed in order to ensure that patients receive due consideration to protect their interests. The investigator is responsible for reporting to the IRB and/or ethics committee to on completion of the study before the scheduled date.

# Safety

## Physical examination

A complete physical examination will be carried out at Visits 0, 12,24,48,72 weeks. The exploration will include consideration of the patient's general appearance, skin, neck (including thyroid), eye, ear, nose, throat, lungs, heart, abdomen, back, lymph nodes, limbs and blood vessels, and a neurological examination. If indicated on the basis of the data of the anamnesis/history and/or symptoms, also will make a rectal examination, external genitalia, breasts and pelvis.

The abbreviated physical examination will include examination of the general appearance of the patient and vital signs (body temperature, blood pressure and respiratory rate and pulse). Abbreviated physical examination at each visit in which is not provided a complete physical examination will be performed.

The information from physical examinations should be included in the source documentation. The relevant findings that are present before administering the first dose of study drug will be stated in the section on medical/relevant current CRD patient disease history. The relevant findings that are observed after the first dose of the study drugs that meet the criteria of adverse event (AE) will be recorded in paragraph CRD adverse patient events.

## Vital signs

Vital signs will be assessed at each visit. They will include body temperature, blood pressure and respiratory rate and pulse.

Once the patient has been sitting for five minutes with his back against the back of the chair and his feet on the ground, the systolic and diastolic blood pressure will be measured three times by a validated device with a cuff of appropriate size. The measurements with the patient seated be obtained at intervals of 1-2 minutes, and the average of the three measurements shall be recorded. If the sleeve of the device is not sufficient to cover the perimeter of the patient's arm, a sphygmomanometer with an appropriate cuff size will be used.

## Body height and weight

Height will be measured in centimeters (cm) at week 0. Body weight (rounded to 0.1 kilogram [kg] closer) will be measured in normal clothes, but barefoot. Weight measurement will be done at all visits.

## Laboratory evaluations

Laboratories of each center will be used for analysis of all samples collected during the study. In CRD laboratory tests to be made during the study and the time of sample collection appear.

## Laboratory tests

**- Analytical Hematology**

As shown in Table 3 analytical hematology tests will be performed at each visit.

**- Biochemical blood analysis**

As shown in Table 3 and Schedule 1, biochemical blood tests will be performed at each visit.

**-Urine analysis**

As shown in Table 3, urine tests will be done at every visit.

**-Electrocardiogram (EKG)**

A conventional 12-lead EKG at weeks 0, 12, 24, 48 and 72 or when a patient withdraws from the study prior to completion will be obtained. The interpretation of the electrocardiographic tracing should be done by a qualified physician and documented in the section of the CRD EKG. All EKG tracings will bear the number of the study, the initials of the patient, the patient's number and date, and stored at the participating center with the source documents. Only abnormalities that are clinically relevant will be documented. Clinically significant abnormalities will be also recorded through the medical/history/CRD relevant current disease history. Clinically significant findings in the EKG ill be notified the sponsor before including the patient in the study.

## Pregnancy and fertility

All women, regardless of their status with regard to self-reported fertility will undergo a pregnancy test from a blood sample during the screening (Visit 1) and from a urine sample once a month Week 2 through Week 72.

A positive result on a pregnancy test from a urine sample requires discontinuation of study drug until the serum level of hCG-B is determined and the result is negative. If positive, the patient should withdraw from the study.

In all animal species studied, RBV has shown to have important teratogenic effects. Therefore, RBV should not be administered to pregnant or intend to become pregnant within a period of less than six months after completion of treatment with ribavirin. RBV will not be administered to men whose partner is pregnant or planning to become pregnant during the study or a period of less than seven months after treatment with RBV.

All patients of childbearing age must undergo a pregnancy test once a month while they are being treated and for a period of six months after the last dose of RBV. The pregnancy test at the scheduled visits will place after the screening visit in the urine.

Cases of pregnancy should be reported to the sponsor as soon as the participating center staff aware of the fact. For more detailed information on the procedures for reporting pregnancies, see Section (pregnancies). They shall be notified and monitor all pregnancies that occur after administration of study drug on Day 1 through Week 48 in the case of patients who completed 24 weeks of treatment or 24 weeks after the last will RBV dose in the case of patients who have received less than 24 weeks of treatment.

## Other tests to evaluate safety

**-Visual acuity**

The visual acuity test will be performed by physicians responsible for the patients. If changes in visual acuity are observed, the patient will undergo a complete eye examination.

**Depression scale**

Depression will be screened by the abbreviated CES-D scale that appears in Appendix 7 at weeks 0, 12, 24, 48. If a depression case is detected it will be evaluated using the Hamilton Depression Scale (Appendix 7).

**-Monitoring of ophthalmic adverse events**

During the routine evaluation of adverse events by the investigator or qualified personnel patients will be ask about ophthalmic adverse events. These questions will be included in the overall assessment of adverse events, which will be asked by other organs or systems. This is done in this way to prevent bias from patients’ self-referred adverse events.

**-Validity of the measures used to assess safety**

Safety assessments chosen for this study are the usual ones in this indication and for this patient population. Some additional security measures have been included in order to monitor the developments observed in preclinical safety studies.

- **Unscheduled visits to assess safety**

Unscheduled visits to assess security at any time during the study are permitted provided that the researcher deemed clinically justified. If you need to make an unscheduled visit, study staff will collect vital signs, adverse events and concomitant medication (if applicable) and carry out those examinations and evaluations deemed necessary.

# ADVERSE EVENTS

An adverse event (AE) is the emergence or worsening of any signs, symptoms or medical condition unwanted after starting treatment with the investigational drug, even when considering that the event is not related to the investigational drug. By study drug means a drug that is being evaluated and the comparator given during any phase of the study. Medical conditions / diseases that were present before starting treatment with the investigational drug are considered AE only if they worsen after starting treatment with the investigational drug. Abnormal values ​​of laboratory tests or any other positive test results are considered AE only if they cause clinical signs or symptoms considered clinically relevant or require treatment.

The presence of AE should be sought by the patient at each visit questions that suggest not push him or her to give an affirmative answer. AE can also be detected when the patient speaks of them spontaneously during or outside visits, by physical examination, laboratory tests or other assessments.

All AE that are detected between the start of treatment with the investigational drug on Day 1 and the end of the study (ie, 24 weeks after the last dose of the study drugs or when a patient withdraws from the study before completion) are recorded through the CRD adverse events.

Monitor patients who experience a serious adverse event (SAE, see definition in section 12.2) that has not been resolved or stabilized before Week 24. contact the patient at least every 12 weeks to be held that the SAE It is resolved or stabilized. If you can not find out what was the outcome of the SAE (p. Eg., The patient was lost to follow-up, has withdrawn its consent), the researcher must document why they have not been able to find out the outcome of the SAE.

All AE will be recorded in the corresponding section of the CRD including the following information:

## Intensity

The AE will be measured using the modified tables toxicity of the Division of Microbiology and Infectious Diseases (IDDM). The following severity criteria for AE not included in these tables apply toxicity:

• Mild: causes no limitation in daily activities of the patient

• Moderate: causes some limitations in daily activities of the patient

• Severe: causes inability to perform usual activities of the patient, can endanger the patient's life * - can cause death or permanent disability.

* Note: The degree of intensity "may endanger the patient's life" it is not necessarily the same as "life-threatening". The latter means that the AE represents an imminent risk to the patient's life, while the first means only that could endanger the patient's life.

## Relationship with the study drugs

• There is suspicion

• There is no suspect

**Duration**

Date of onset and resolution or still present in the final evaluation.

**Severity**

Note if it is a SAE.

An SAE is a AE that is:

• fatal or may endanger the patient's life

• causes a significant or persistent disability

• is an anomaly or birth defect

• requires inpatient hospitalization or prolongation of hospitalization if the patient was already in hospital, unless:

• hospitalization is to control or carry out a routine procedure unrelated study indicating a deterioration of the disease.

• hospitalization is due to a pre-programmed treatment for an illness or pre-existing condition that has no relation to the indication of the study and has not worsened since the beginning of the treatment with the investigational drug.

• emergency treatment as an outpatient by some event that does not meet any of the criteria for the definition of SAE cited above and does not require hospitalization.

• hospitalization is due to social reasons or causes for the patient or caregiver usual rest, without having produced any deterioration in the general condition of the patient.

• AE is medically relevant (ie., Endangering the patient or may require medical or surgical intervention to prevent one of the situations mentioned above).

Unlike the case with routine safety assessments, the SAE should be continuously monitored and follow special procedures for notification (see section 12.4).

All AE must be treated properly. The actions taken to address the AE will be recorded in paragraph CRD adverse events.

The investigator manual (IM) contains information on known investigational drug side effects. If between an upgrade and over the IM, the study sponsor becomes aware of side effects of investigational drug not known until that moment, researchers communicated through Notification to the investigator. This should be included in the information sheet for informed consent request and, if necessary, will explain to the patient during the study.

## Adverse reaction

An adverse reaction (AR) is any harmful and unintended reaction to a drug investigation, regardless of the dose. In an AR there is a suspicion of a causal relationship between the drug and the adverse event investigation. The causal relationship is suspected by the corresponding researcher basing it on the profile of known adverse reactions and included in the technical specifications of nitazoxanide, Peg-IFN alfa-2b and RBV. Serious adverse reaction (SAR) is one that is fatal or may endanger the patient's life, causing significant or persistent disability is an anomaly or birth defect, requires hospitalization or prolongation of hospitalization if the patient He was already hospitalized. SAR are considered those that are medically relevant, though not meet the above criteria SAR (p. Eg., Endangering the patient or may require medical or surgical intervention to prevent one of the situations mentioned above). They also reported as serious all suspected transmission of an infectious agent through a drug.

An unexpected adverse reaction (UAR) is any reaction, the nature or consequences do not correspond to the reference information for the drug. The unexpected nature of an AR is based on fact not been previously observed and not be anticipatable by the pharmacological properties of the drug.

## Notificación de los acontecimientos adversos graves

To ensure patient safety, all SAE, regardless of whether there is suspicion of a causal relationship to drug research, and all SAR or UAR that appear once the patient has started taking the investigational drug and up to 30 days after finishing the patient's participation in the study will be reported to the sponsor.

The SAE, SAR or UAR appearing after this period of 30 days will be notifed the sponsor only if the investigator has suspected a causal relationship to the investigational drug. Recurrences, complications and progress of the SAE, SAR or UAR will be reported as part of the follow-up provided the initial episode, regardless of when they occur. The notification must be made within 24 hours from the time when the researcher receives tracking information. If you consider that a SAE, SAR or UAR is not at all related to another SAE, SAR or UAR that has already been notified, the investigator must notify as a new SAE, SAR or UAR.

Information on SAE, SAR or UAR will be collected and recorded in the form of serious adverse events. The investigator must evaluate the possible relationship between SAR or SAE and the investigational drug, fill in the form of serious adverse events, sign it and fax it to telephone 955015461. The researcher file includes the phone number and fax of the person responsible for the Safety and Clinical Epidemiology. The original of the form for reporting SAE or SAR and the confirmation faxing should be kept as part of the CRD at the participating center.

The same person tracking information SAE or SAR will send the notification form to SAE or SAR, using a new form that will be noted that this is the track a SAE or SAR already notified and the date of the original form of the SAE or SAR. In the tracking information will specify whether the SAE or SAR is resolved or continues, whether it was or not treated, how it has been treated and the patient continues to participate in the study or was removed.

If the SAE or SAR is not documented in the IM or product insert (new SAE or SAR) and is considered to be related to the investigational drug, the sponsor may request more information urgently on the SAE or SAR order to inform health authorities. The promoter sent to all researchers involved in the study a Notice to the researcher, reporting that has notified a new SAR or UAR. The UAR be recorded and reported by the developer to the competent health authorities and ethics committees in compliance with RD 233/2003 of February 6 and/or existing laws and regulatory standards, specifically:

• Notification of UAR associated with nitazoxanide

The notification shall be made by fax (918 225 076). Suspected adverse reactions are accompanied by the accompanying letter.

• Notification UAR associated with pegylated interferon alfa 2-b ribavirin

The notification shall be made by fax (918 225 336). Suspected adverse reactions are accompanied by the accompanying letter indicated.

• Expedited reporting of UAR the Ethics committee and AA.CC.

It shall be notified to each of the Ethics committee involved in a clinical trial UAR all that had occurred in the participating centers in its area of ​​influence subjects. It will also notify the competent organ of each of the Autonomous Communities where the test is carried RAGI suspicions that occurred in health facilities in their community. In both cases, it will be used for the notification form.

The developer made the notification in electronic format all UAR also occurred to Eudravigilance-CT.

## Pregnancy

To ensure patient safety, all participants of reproductive age (women and men) will be explained that they should avoid pregnancy and thus must use two forms of birth control from the baseline to 6 months after completion the controller or the last dose of RBV (later of the two dates). All pregnancies that occur after the patient has started taking the study drugs and up to six months after the last dose of RBV (depending on which of these two dates is later) should be reported to the sponsor within 24 hours counting from the time that pregnancy is known. Monitoring of pregnancy must be done to determine the outcome of the transaction, including spontaneous abortion, abortion, birth details and the presence or absence of any defect or congenital anomaly or complications in the mother or newborn. Pregnancies that occur after signing the informed consent before the patient enters the study exclusion criteria will be considered.

The pregnancy tracking information is recorded in the CRD and evaluate the possible relationship between the investigational drug and the outcome of pregnancy is included. The SAE or SAR appearing during pregnancy will be recorded in the form of SAE or SAR.

You should also register the outcome of the pregnancy of the pair of male patients involved in the study. In these cases, the mother must consent to obtain such information.

## Data and safety monitoring board

A Data and Safety Monitoring Board (DSMB) will be constituted. It will consist of the principal investigators and the sponsor.

The DSMB is governed by a set of rules to be drafted before the first visit of the first patient. These standards recommendations for adjusting the dose of study drug will be included (if necessary), the criteria for removing a patient, the criteria for suspending a treatment group and the criteria for suspending the study.

DSMB need to immediately evaluate the SAE or SAR during the first 3 months of treatment, in order to provide appropriate recommendations on the amendment / suspension, etc. treatment. During the treatment period including the reference treatment, patients can be treated in accordance with the guidelines set out in the protocol and, once treatment ended, researchers take the final decisions they deem most appropriate for their patients.

In the performance standards of DSMB periodicity and mechanisms of communication between researchers, the DSMB and the sponsor is specified.

# ETHICAL ASPECTS/PROTECTION OF THE PARTICPATING SUBJECTS

This clinical study was designed, will be made and published in full compliance with the provisions of the Guidelines on Good Clinical Practice, as well as the provisions of laws and administrative regulations of Spain (including the European Directive 2001/20/EC). Also it respects the ethical principles of the Declaration of Helsinki.

## Risk-benefit assessment

The patients included in this trial will receive the current standard treatment against HCV, Peg-IFN plus RBV, which its safety and efficacy in HIV/HCV subjects are known. In addition, this standard treatment NTZ, a drug widely used as an antiparasitic, including experience of use in HIV-infected subjects will be added. Nitazoxanide was safe in the context of treatment of parasitic diseases. Furthermore, among HCV-monoinfected patients, the use of more NTZ Peg-IFN and RBV did not significantly increase the adverse effects associated to the combination of Peg-IFN plus RBV. Finally, the addition of NTZ to standard treatment against HCV showed a significant increase in SVR rate of patients infected with HCV-4. Therefore, the risk-benefit of a combination of NTZ's Peg-IFN plus RBV in patients coinfected with HIV/HCV-4 will be favorable.

## Information sheet and consent form

Each subject asked to participate in the study, it will be given a written document entitled "Patient Information Sheet", in which, in detail, the purpose and description of the study, study procedures, refer duration Preview and number of subjects involved, benefits and possible risks arising from their participation therein, confidentiality of personal data and contact details of the physician responsible for the study. This document shall be written in words that allow their contents are fully readable and understandable to the patient.

The investigator should inform the patient about the voluntary nature of their participation and involving no change in either treatment or medical care compared to those who receive not participate. He will answer your doubts and questions and in accordance with current regulations obtain the subject's consent or otherwise, of an impartial witness, in which case he will sign the informed orally before witnesses (Exhibit 8) consent.

The subject participating in the study may at any time revoke your consent to the use of your data in the analysis, without explanation and without necessarily incurred by the liability or prejudice.

## Confidentiality of data

The collection of data will be done by completing an electronic database made specifically for this study. Monitoring data of patients should be included in the base until the end of the study period. Project coordinators will have access to the entire database, but cannot modify data already entered. Only the final responsible of the file and its analysis can correct input errors or assessment in consultation with the responsible physician for each patient.

Technical and organizational measures to ensure the security of personal data and avoid its alteration, loss, or unauthorized access measures taken. No data to identify the patient as a coding system whose real correlation only be known by the investigator responsible for each center will be used will be introduced.

The processing of personal data required in this study is governed by Law 15/1999 of December 13 Protection of Personal Data. In order to ensure the confidentiality of the survey data, only will access to them, the researcher and his team, the sponsor or the person designated by the Clinical Research Ethics Committee, the relevant health authorities and responsible for analyzing them.

The content of the electronic database where information is recorded, is encrypted and protected from uses not permitted by persons outside the research and therefore will be treated as strictly confidential and will not be disclosed to third parties except those specified in paragraph above.

## Responsibilities of the investigator and the Ethics Committee

Before the start of the study, the protocol and the model fact sheet for patient/informed consent form must be reviewed and approved by an ethics committee constituted in accordance with regulations. Before the study begins, the participating centers will send to the sponsor a signed and dated document in which it is shown that the protocol and patient information sheet/informed consent form were approved by the Ethics committee. Also, before the start of the study, the investigator will be asked to sign the signature page of the protocol to confirm acceptance of the study in accordance with the instructions and procedures contained in the protocol and agrees to allow access to all documents relevant to the study monitors and representatives of the Ethics committee and regulatory authorities if required. If regulatory official bodies request an inspection of the institution participating in the study, the investigator shall inform the sponsor immediately.

# PLAN OF DISEMINATION OF RESULTS

All information related to the study are considered confidential and property of the sponsor until its publication, it may not be disclosed to others without prior written consent of the sponsor and may not be used except for this study.

Only the sponsor or his representatives may make extensible physicians and regulatory agencies the information obtained in this study, except if required by order.

The study results will be published in scientific journals and/or disseminated through conference papers.

Policy regarding authorship and publication of results, the standards set by the sponsor shall be observed:

- The publication of the study will be conducted in scientific journals with reference to the Ethics Committees of Clinical Research.

- The order of authors in publications is as follows: first author of the study coordinator researcher appear as co-authors and other researchers in order of appearance according to the number of patients recruited, and taking into account the limited number Authors established by the publisher of the journal to which the manuscript is submitted for publication.

- When making public the development and study results, in any case, shall state the source of funds for its implementation.

- The anonymity of the cases included in the study will be maintained at all times.

- The results or conclusions of this study will be reported as a priority, in scientific journals before being released to the public health no.

# FUNDING

The sponsor, Dr. Juan Macias Sanchez, guarantees non-interference in the selection process of cases, analysis of information and/or presentation of results, or any other process that may affect the study results.

The financing of the study according to the guidelines of this protocol come from the 2010 Aid to Promote Independent Clinical Research of the Ministry of Health, Social Policy and Equality (Ayudas para el Fomento de la Investigación Clínica Independiente del Ministerio de Sanidad, Política Social e Igualdad, file EC10-187). The funding will in any case independent of the study results.

In any case the study sponsor will have a financial report of the study will be available for inspection at the time needed.

# AMENDMENTS TO THE PROTOCOL

All changes made in this Protocol shall be communicated to the Ethics committee. In the case of relevant amendments, those that affect fundamental aspects of the study protocol objectives, methods and ethical aspects, it will be submitted back to Ethics committee assessment that reported favorably on it, and administrative authorization for such amendment is required. For other amendments will be sufficient notice to Ethics committee, justifying why it is not considered relevant.

## Adherence to protocol

Researchers should make every effort to avoid protocol deviations. Under no circumstances the investigator will contact the promoter to apply for permission to make a departure from protocol, since no violation of the same is not allowed. If the investigator believes that a violation of the protocol would improve the development of the study, you should consider that what is proposed is an amendment to the protocol, and may not implement the amendment if not previously approved by the promoter and the corresponding Ethics committee. All relevant protocol violations are recorded and reported in the CRD.

# PRACTICAL CONSIDERATIONS

1. ***Monitoring reports and final***

The final closure of the trial will be conducted once they have completed all the data from the last patient included in the study. After the closure of the database statistical analysis was conducted and a report with the descriptive data, which will be reviewed and approved by the promoter of the study will be presented.

The developer will write two reports half-yearly monitoring and a final report in accordance with current legislation. Likewise, the promoter shall report immediately any significant impact (major security disruption, etc.) that may occur in the course of the study. All such communications will be presented to the competent bodies of the Autonomous Communities and the Competent Authority involved.

A final report between 3 and 6 months after completion of the study to the Competent Authority and the competent bodies of the autonomous regions where it was made shall be submitted.

## b. Dissemination of results

It will be conducted as described in paragraph 14.

## c. Responsibilities promoter

The responsibilities of the sponsor of the study are:

- Sign with the researcher coordinator protocol and any amendment thereto.

- Provide research protocol and the data sheet of the study drugs if applicable

- Forward the protocol to the CEIC.

- Order the sanction of the Administration, as appropriate, and submit documentation.

- Submit progress reports and final, within the time limits and communicate, where appropriate, the interruption and the reasons therefor.

- Provide a copy of the protocol and the documents establishing monitoring procedures established in these guidelines to the responsible entities providing services where health care is going to conduct the study.

- Apply quality control in the production and handling of data to ensure that the data are reliable.

- Identify sources of funding for the study.

- Sign, if applicable, the contract with the competent body.

- Make public the results of the study, if possible, through a scientific journal.

## d. Researcher's Responsibilities

The responsibilities of the investigator at each participating center will be:

- Sign a commitment that is recognized as study investigators and claim to know the protocol and any amendment thereto, and agree with him in all respects.

- Inform research subjects and obtain their consent.

- Collect, record and report data properly responding to updating and appropriate quality audits.

- Report adverse events to the sponsor as established in the protocol.

- Respect the confidentiality of the data of the subjects participating in the study.

- Facilitate promoter audits and inspections by health authorities.

- Know how to respond on the objectives, methodology and basic meaning of the study results to the scientific and professional community.

## e. Responsibilities of the coordinator researcher

The responsibilities of coordinating investigator of the study are those listed above for the researcher plus the following:

- Signing the protocol and any amendment thereto together with the promoter.

- Co-responsibility with the promoter of the development of the monitoring reports and final.

- Helping to disseminate the results of the study, in cooperation with the sponsor.

# REFERENCES

1. Torriani FJ, Rodriguez-­‐Torres M, Rockstroh JK et al. Peginterferon alfa-2a plus ribavirin for chronic hepatitis C virus infection in HIV-infected patients. N Engl J Med 2004; 351: 438-450.

2. Carrat F, Bani­Sadr F, Pol S et al. Pegylated interferon alfa-2b vs standard interferon alfa-­‐2b, plus ribavirin, for chronic hepatitis C in HIV‐infected patients: a randomized controlled trial. JAMA 2004; 292: 2839­2848.

3. Nunez M, Miralles C, Berdun MA et al. Role of weight­based ribavirin dosing and extended duration of therapy in chronic hepatitis C in HIV-infected patients: the PRESCO trial. AIDS Res Hum Retroviruses 2007; 23: 972-­‐982.

4. Mira JA, López-Cortés LF, Barreiro P, et al. Efficacy of pegylated interferon plus ribavirin treatment in HIV/hepatitis C virus co-infected patients receiving abacavir plus lamivudine or tenofovir plus either lamivudine or emtricitabine as nucleoside analogue backbone. J Antimicrob Chemother 2008; 62: 1365-1373.

5. Martín-Carbonero L, Puoti M, García-­‐Samaniego J, et al. Response to pegylated interferon plus ribavirin in HIV-­‐infected patients with chronic hepatitis C due to genotype 4. J Viral Hepat 2008;15: 710-715.

6. Korba BE, Montero AB, Farrar K, et al. Nitazoxanide, tizoxanide and other thiazolides are potent inhibitors of hepatitis B virus and hepatitis C virus replication. Antiviral Res 2008; 77: 56-63.

7. Rossignol JF, Elfert A, El-­‐Gohary Y, Keeffe EB. Improved virologic response in chronic hepatitis C genotype 4 treated with nitazoxanide, peginterferon, and ribavirin. Gastroenterology 2009; 136: 856-862.

8. Rossignol JF, Elfert A, Keeffe EB. Treatment of chronic hepatitis C using a 4-week lead-­‐in with nitazoxanide before peginterferon plus nitazoxanide. J Clin Gastroenterol 2009 (en prensa, publicado online).

9. Rossignol JF, Elfert A, El-Gohary Y, Keeffe EB. Randomized controlled trial of nitazoxanide-peginterferon-ribavirin, nitazoxanide-peginterferon and peginterferon-ribavirin in the treatment of patients with chronic hepatitis C genotype 4. 43rd EASL. Milán (Italia), 23-27 de abril de 2008. Abstract 68.
